# Systematic review of fNIRS studies reveals inconsistent chromophore data reporting practices

**DOI:** 10.1117/1.NPh.9.4.040601

**Published:** 2022-12-23

**Authors:** Kaleb T. Kinder, Hollis L. R. Heim, Jessica Parker, Kara Lowery, Alexis McCraw, Rachel N. Eddings, Jessica Defenderfer, Jacqueline Sullivan, Aaron T. Buss

**Affiliations:** a University of Tennessee, Department of Psychology, Knoxville, Tennessee, United States; b University of Tennessee Health Science Center, Department of Audiology and Speech Pathology, Knoxville, Tennessee, United States

**Keywords:** functional near-infrared spectroscopy, neuroimaging, data reporting practices, neurovascular coupling, hemodynamic response

## Abstract

**Significance:**

Functional near-infrared spectroscopy (fNIRS) is unique among neuroimaging techniques in its ability to estimate changes in both oxyhemoglobin (HbO) and deoxyhemoglobin (HbR). However, fNIRS research has applied various data reporting practices based on these chromophores as measures of neural activation.

**Aim:**

To quantify the variability of fNIRS chromophore data reporting practices and to explore recent data reporting trends in the literature.

**Approach:**

We reviewed 660 fNIRS papers from 2015, 2018, and 2021 to extract information on fNIRS chromophore data reporting practices.

**Results:**

Our review revealed five general practices for reporting fNIRS chromophores: (1) HbO only, (2) HbR only, (3) HbO and HbR, (4) correlation-based signal improvement, and (5) either the total (HbT) or difference (HbDiff) in concentration between chromophores. The field was primarily divided between reporting HbO only and reporting HbO and HbR. However, reporting one chromophore (HbO) was consistently observed as the most popular data reporting practice for each year reviewed.

**Conclusions:**

Our results highlight the high heterogeneity of chromophore data reporting in fNIRS research. We discuss its potential implications for study comparison efforts and interpretation of results. Most importantly, our review demonstrates the need for a standard chromophore reporting practice to improve scientific transparency and, ultimately, to better understand how neural events relate to cognitive phenomena.

## 1 Introduction

Functional near-infrared spectroscopy (fNIRS) is a neuroimaging technique that has rapidly risen in popularity over recent decades^1^^–^^3^ due to several advantages it presents to researchers for examining brain function (see Ref. 4 for an overview). For instance, fNIRS is safe and portable, suiting laboratory and ecologically valid contexts while offering resistance to motion artifacts. Consequently, fNIRS has provided opportunities to work with diverse populations and settings^5^^,^^6^ in ways alternative neuroimaging modalities are limited or incompatible.

**Fig. 1:**
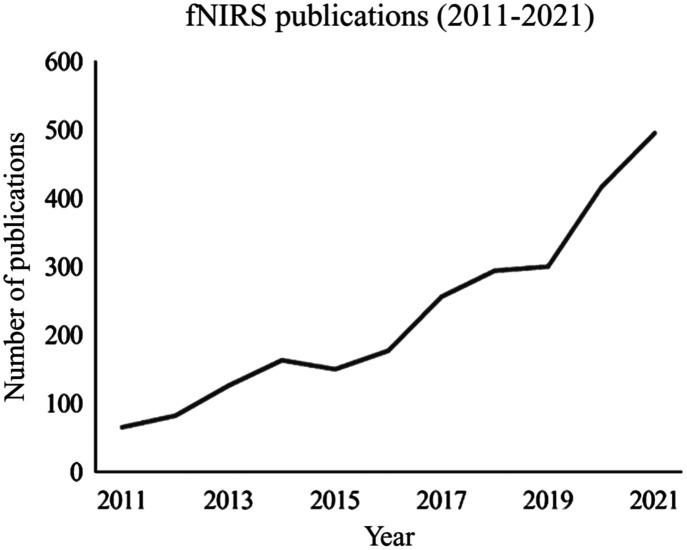
PubMed search results for the search query “fNIRS” from 2011 to 2021.

Although fNIRS stands as a promising neuroimaging tool, there is a need for more standardized steps related to its protocols, particularly considering its utilization is rapidly growing (Fig. 1). A recent collaborative paper proposed a consensus of guidelines for conducting and presenting an fNIRS project to enhance the interpretation and replicability of its methods and findings.^7^ Similar lines of work have demonstrated how differences in data preprocessing and data analysis procedures can lead to inconsistent results.^8^^–^^10^ An additional growing concern is that there may be data reporting inconsistencies among researchers regarding the signals that fNIRS monitors as a proxy of neural activation.^11^^,^^12^ Differences in how fNIRS researchers operationalize “neural activation” could pose significant challenges for study comparisons and meta-analysis approaches. However, the extent to which chromophore data reporting practices vary across the fNIRS research field has yet to be systematically examined. In this paper, we focus on the issue of chromophore reporting in the fNIRS literature.

A distinct advantage of fNIRS compared to other neuroimaging modalities is its ability to estimate changes in the hemodynamic response by measuring both chromophores of oxyhemoglobin (HbO) and deoxyhemoglobin (HbR). fNIRS accomplishes this by emitting different wavelengths (e.g., 690 and 830 nm) of near-infrared light into the cortex of the brain (penetration depth of ∼2  cm) that are differentially absorbed by HbO and HbR.^13^ fNIRS indirectly measures neural activation by targeting these chromophores following a cognitive event as part of the brain’s metabolic process.^14^ This process, termed neurovascular coupling, unfolds over several seconds, peaking at ∼5  s postneuronal activity.^15^ Excess HbO is sent to the tissue surrounding the activated cells during this period, resulting in an increase in HbO concentration and a concurrent but weaker decrease in HbR concentration.^16^ Therefore, a significant change in neural activation can be interpreted by a negative correlation between HbO (increasing) and HbR (decreasing). However, various ways of using HbO and HbR as dependent measurements of brain activation have been used in the fNIRS literature.

There has been a recent push for the fNIRS research community to move toward more standardized practices to improve the interpretation and transparency of results (e.g., see Refs. 7 and 10). Given the nature of neurovascular coupling, it is informative to report data about both chromophores in studies of neurocognitive function. There are two aims of this paper: (1) to quantify the variability of chromophore data reporting practices in the fNIRS literature and (2) to explore recent trends in fNIRS chromophore reporting. Our strategy was to systematically review fNIRS articles published in 3-year increments between 2015 and 2021 to extract information on chromophore reporting. Based on the heterogeneity of practices in the field, we then provide a recommendation for fNIRS chromophore reporting that is grounded in the neurovascular coupling process.

## 2 Literature Review Methods

### 2.1 Search Strategy

**Fig. 2:**
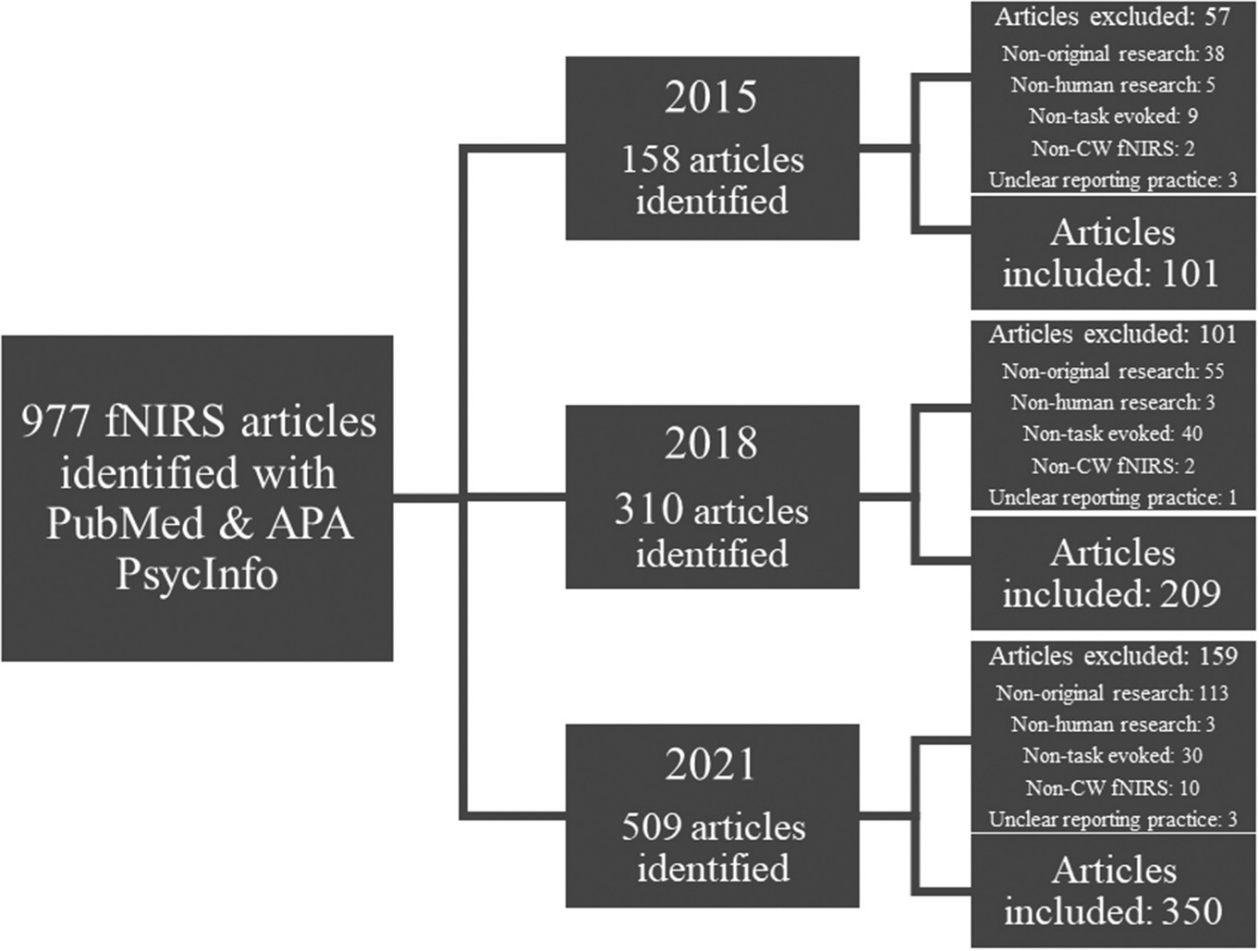
Flowchart detailing the search strategy and selection criteria.

We performed a literature review of fNIRS articles published in the field of cognitive neuroscience to quantify the prevalence of different chromophore data reporting practices. We used the PubMed and APA PsycInfo databases to manually search for 2015, 2018, and 2021 articles (excluding duplicates) using the specific keyword “fNIRS.” An overview of the article search and selection process is illustrated in Fig. 2.

### 2.2 Inclusion Criteria

Our initial search identified 977 articles in 2015 (n=158), 2018 (n=310), and 2021 (n=509). The title, abstract, and full text of each identified article was accessed and screened to determine its eligibility for review. A total of 660 fNIRS articles from 2015 (n=101), 2018 (n=209), and 2021 (n=350; see the Supplementary Material for a list of 2015, 2018, and 2021 references) met our inclusion criteria for further review. Articles were selected for review based on the following criteria.

1. Articles published in 2015, 2018, and 2021.
2. Original research articles.
3. Articles that included task-evoked changes in activation (resting-state studies were excluded).
4. Articles that used continuous-wave fNIRS.
5. Articles in human cognitive neuroscience research.
6. Articles that performed a clear chromophore data reporting practice.

### 2.3 Extracted Information

To examine recent chromophore data reporting practices, we extracted the following information from each fNIRS article that met our inclusion criteria.

1. The chromophore or combination of chromophores reported to interpret changes in neural activation.
2. The justification (if any) provided for the data reporting practice if only one chromophore was reported.

If there was ambiguity regarding an article’s eligibility criteria or chromophore reporting practice, the researchers (K.T. K., H. L. R. H., J. P., K. L., J. S., and A. M.) discussed its contents until a group consensus was reached. We considered a chromophore or combination of chromophores (e.g., HbT) as “reported” if its concentration value was directly stated in the results or if a visualization was provided in the main text or Supplementary Material. Extraction of chromophore preprocessing steps and statistical analysis approaches was not performed. The articles for the 2015, 2018, and 2021 literature searches were retrieved on September 13, 2022, November 25, 2019, and March 3, 2022, respectively.

## 3 Literature Review Results

Our review revealed various practices for reporting fNIRS chromophores that we categorized into five groups: (1) HbO only, (2) HbR only, (3) HbO and HbR, (4) correlation-based signal improvement (CBSI),^17^ and (5) either the total (HbT) or difference (HbDiff) in concentration between chromophores. We categorized articles into each data reporting approach based on whether individual information was provided for each chromophore. For example, if an article reported HbO, HbR, and HbT, we categorized it as reporting HbO and HbR, as information for each chromophore was made available. As another example, if an article reported HbO and HbDiff, it was categorized as reporting HbO only, because individual information was only explicitly provided for HbO. Although HbDiff includes contributions of each chromophore (HbO–HbR), reporting HbDiff does not directly detail each chromophore’s concentration value unless that information is otherwise provided.

### 3.1 2015 fNIRS Chromophore Data Reporting Practices

**Fig. 3:**
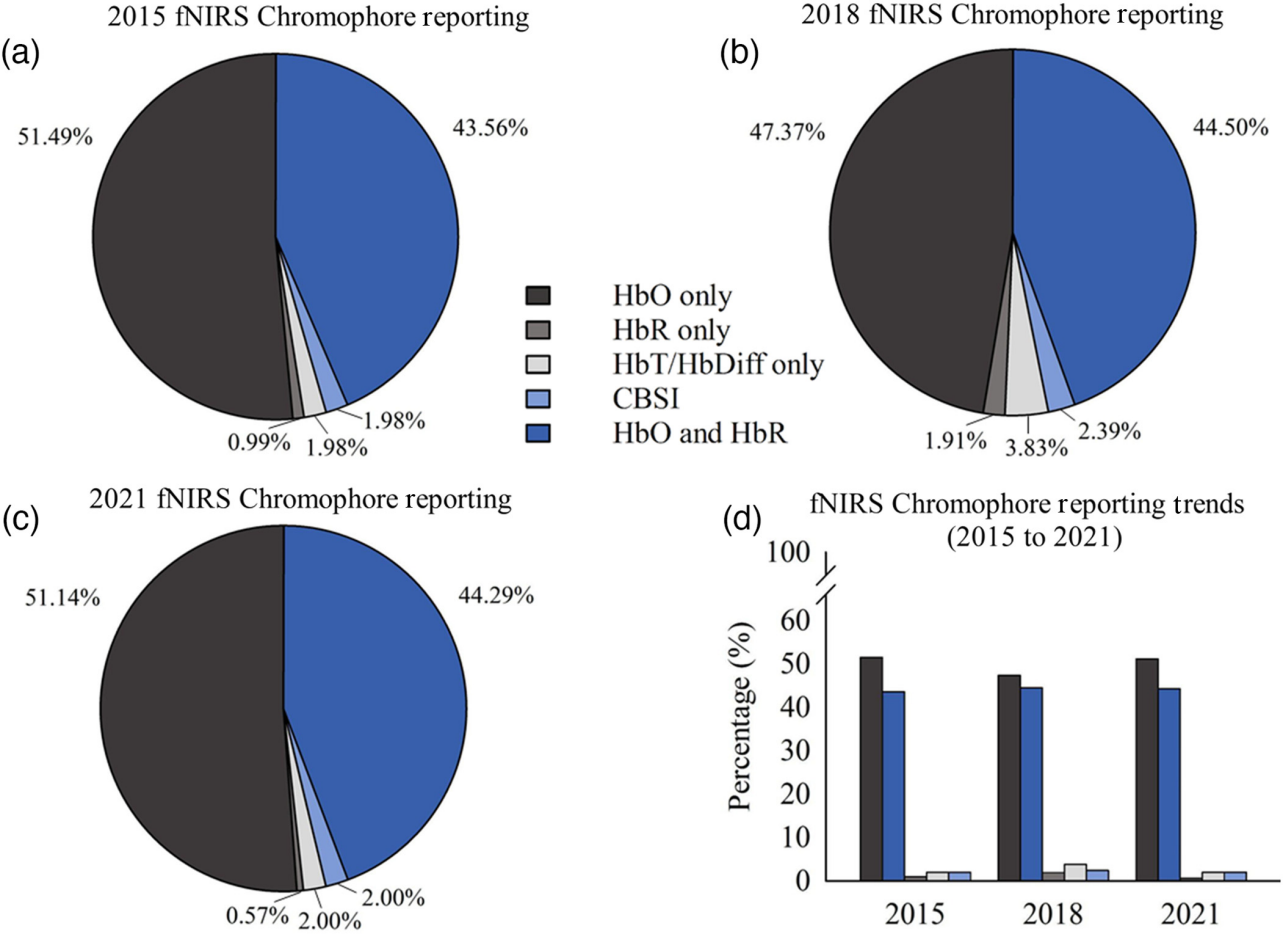
fNIRS chromophore data reporting practices in 2015, 2018, and 2021: (a) 2015, (b) 2018, and (c) 2021 fNIRS chromophore reporting and (d) fNIRS chromophore reporting trends from 2015 to 2021.

The majority of studies in 2015 reported only one chromophore, HbO (51.49%). 43.56% of articles reported both HbO and HbR chromophores. Data reporting practices that used transformed chromophore data, HbDiff or HbT only (1.98%), CBSI (1.98%), and HbR only (0.99%), were far less popular. These results are presented in Fig. 3(a).

### 3.2 2018 fNIRS Chromophore Data Reporting Practices

The two most common fNIRS chromophore reporting practices for 2018 were reporting HbO only (47.37%) and reporting HbO and HbR (44.50%). This result was consistent with our 2015 review findings. Only 3.83% of articles reported HbDiff or HbT alone, 2.39% reported CBSI, and 1.91% only reported HbR. A breakdown of chromophore reporting practices from 2018 can be seen in Fig. 3(b).

### 3.3 2021 fNIRS Chromophore Data Reporting Practices

The majority of the fNIRS field in 2021 again reported HbO only (51.14%), followed by reporting HbO and HbR (44.29%). A low number of papers reported HbDiff or HbT only (2.00%), CBSI (2.00%), or HbR only (0.57%). A breakdown of chromophore reporting practices from 2021 can be seen in Fig. 3(c).

### 3.4 fNIRS Chromophore Data Reporting Trends (2015 to 2021)

From 2015 to 2021, fNIRS data reporting practices were heavily divided between reporting one chromophore (HbO) or both HbO and HbR chromophores. However, reporting HbO only was consistently the most popular practice for each year reviewed. This result indicates that most articles omitted HbR information from publication. Clearly, there is a divide in the fNIRS community regarding which practice is better suited for characterizing neural activation. The trends in fNIRS chromophore reporting practices from 2015 to 2021 can be seen in Fig. 3(d).

### 3.5 Justifications Provided for only Reporting HbO (2015, 2018, and 2021)

Given the most popular practice was to report only one chromophore (HbO), we next examined the justifications for applying this approach. We coded the justifications provided for 2015, 2018, and 2021 articles that reported only one chromophore. Due to the low number of articles that reported HbR only (N=7), we decided to focus on the justifications provided for only reporting HbO (N=330). If multiple justifications were provided for only reporting HbO, we simply coded the justification listed first.

**Fig. 4:**
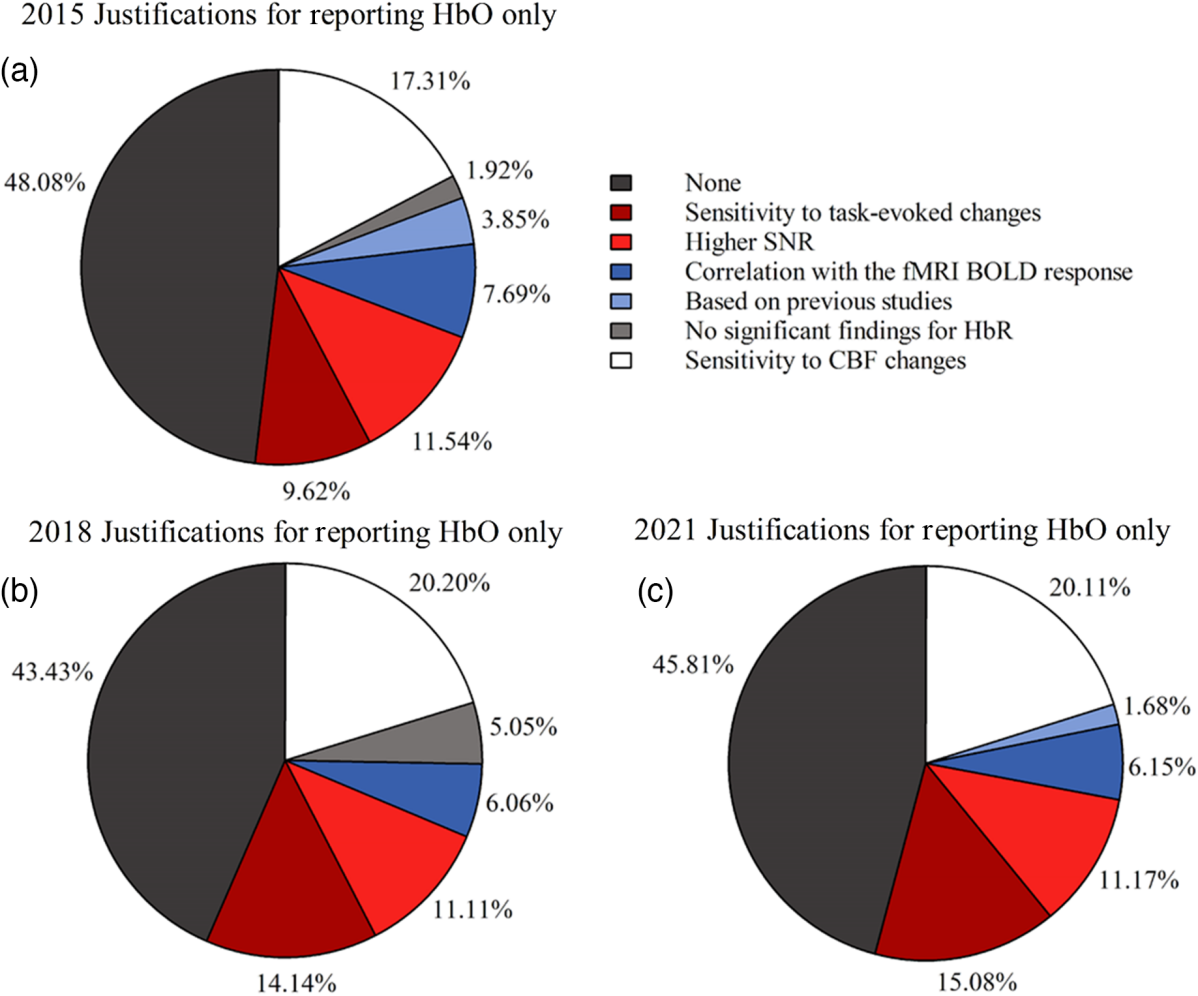
Justifications provided for reporting HbO only in 2015, 2018, and 2021: (a) 2015, (b) 2018, and (c) 2021 reporting HbO only justifications.

We categorized each 2015 article that reported HbO only [n=52; Fig. 4(a)] into six groups of justifications: (1) greater sensitivity to cerebral blood flow (CBF) changes compared to HbR (17.31%), (2) higher signal-to-noise ratio (SNR) than HbR (11.54%), (3) greater sensitivity to task-evoked changes than HbR (9.62%), (4) stronger correlation with the fMRI BOLD response than HbR (7.69%), (5) followed the practice of previous studies (3.85%), and (6) no significant findings found for HbR (1.92%). The remaining 48.08% of articles did not provide a justification for choosing to only report HbO.

Our 2018 review revealed several justifications for only reporting HbO [n=99; Fig. 4(b)] that we categorized into five groups: (1) greater sensitivity to CBF changes than HbR (20.20%), (2) greater sensitivity to task-evoked changes than HbR (14.14%), (3) higher SNR than HbR (11.11%), (4) stronger correlation with the fMRI BOLD response than HbR (6.06%), and (5) no significant findings found for HbR (5.05%). However, for 43.43% of articles, no justification was provided for only reporting HbO.

Our 2021 review also revealed various justifications for only reporting HbO [n=179; Fig. 4(c)]. We categorized these justifications into five groups: (1) Greater sensitivity to CBF changes than HbR (20.11%), (2) greater sensitivity to task-evoked changes than HbR (15.08%), (3) higher SNR than HbR (11.17%), (4) stronger correlation with the fMRI BOLD response than HbR (6.15%), and (5) followed the practice of previous studies (1.68%). Again, in 2021, a large portion of articles did not justify only reporting HbO (45.81%).

Within the most popular fNIRS chromophore reporting practice of HbO only, there was considerable variability in the reasons why this approach was chosen. Overall, the justifications provided in 2015, 2018, and 2021 for reporting HbO only were generally consistent [Figs. 4(a)–4(c)]. However, nearly half of these articles (45.8%) did not justify why HbR data were excluded from publication and for choosing to only present HbO data.

## 4 Discussion

The use of fNIRS to investigate cognitive phenomena is rapidly expanding, but a consensus regarding its best practices is still emerging. In this systematic review, we quantified the variability of fNIRS chromophore data reporting practices and explored recent data reporting trends in the field. We reviewed 660 articles published in 2015, 2018, and 2021, which applied different chromophore measurements as indirect markers of neural activation. Overall, we found high heterogeneity in the fNIRS chromophore data reporting practices used to interpret neural activation and draw conclusions. The most common practice was to report only one chromophore, HbO. However, there was strong disagreement in the fNIRS field over which practice should be implemented, split between two groups: only reporting HbO and reporting both HbO and HbR. Other chromophore data reporting practices (HbR only, CBSI, and HbT/HbDiff only) were rarely performed in comparison.

The major divide between reporting one or both chromophores in fNIRS research raises the important question of how neural activation should be operationalized in the field and whether the variability in current practices may produce different interpretations of results. In our review, the justifications (if any) provided for only reporting HbO as an indirect neural activation marker were diverse. Indeed, reporting HbO alone may be compelling for several reasons, including its sensitivity to CBF changes,^18^^–^^20^ sensitivity to detecting task-evoked changes,^21^^,^^22^ and its stronger correlation with the fMRI BOLD response than HbR (but see Ref. 23).^24^ However, these justifications do not warrant the omission of HbR data. Rather, we consider the exclusion of HbR data as a critical missed opportunity to enrich our interpretation of the hemodynamic response and to establish evidence for neural activation. In the following section, we discuss potential issues related to reporting only one chromophore and provide general recommendations for reporting fNIRS chromophore data in publications.

### 4.1 Considerations and General Recommendations for fNIRS Chromophore Data Reporting

Operationalizing neural activation in the fNIRS literature is based on neurovascular coupling between local neural activity and subsequent changes in the hemodynamic response. Following the expected hemodynamic response, neural activation is indicated by an increase in HbO, accompanied by a weaker decrease in HbR. In other words, establishing a negative correlation between HbO and HbR should provide strong evidence that observed blood flow changes result from neural activation. We argue that the primary issue with reporting one chromophore is the assumption that there is a negative correlation between chromophores (i.e., activation) when significant differences are found between conditions. Crucially, it has been demonstrated that this assumption can be violated.^11^ For example, let us say that condition 1 ($C_{1}$) and condition 2 ($C_{2}$) are used to assess some effect on brain function and that there is a significant difference in HbO concentration between $C_{1}$ and $C_{2}$. Based on the practice of only reporting HbO, the conclusion might be that there is greater activation in $C_{1}$ compared to $C_{2}$. Now let us consider a scenario in which reporting both chromophores provide an alternative interpretation. Although HbO is significantly higher in $C_{1}$ than in $C_{2}$, there is no significant difference between HbO and HbR within $C_{1}$ or $C_{2}$. Therefore, there is no evidence to suggest a meaningful change in neural activity within or between conditions when both chromophores are considered. Recent research reveals several instances in which significant effects are found when reporting one chromophore but not when reporting both chromophores.^11^^,^^12^ These studies demonstrate how different interpretations of results can occur depending on which chromophore reporting practice is performed.

In addition to establishing a negative correlation to better identify activation, reporting both chromophores may facilitate the detection of non-neuronal influences on the hemodynamic response. For example, motion artifacts,^17^ cross-talk between chromophores,^25^ and spatially localized extracerebral changes (e.g., recordings over the anterior temporal region^12^^,^^26^) have instead been associated with positive correlations between HbO and HbR. These findings further highlight how reporting one chromophore can lead to misinterpretation of results, such as a positive correlation between chromophores going undetected. Concerns regarding this practice are heightened, considering our finding that reporting HbO only has consistently been the most popular chromophore reporting practice in the fNIRS community from 2015 to 2021.

Our general recommendation for reporting fNIRS data is based on the principles of neurovascular coupling and its expected hemodynamic response. We recommend that both HbO and HbR data should be reported to establish that they are, in fact, negatively correlated in conditions where activation is being inferred. As a minimum requirement, we propose reporting data for both chromophores, whether it be their statistical outcomes or relevant visual information (e.g., time-series plotted for each chromophore; see Ref. 7 for a guideline). Although the issue of preprocessing and statistical approaches for analyzing chromophores was beyond the scope of this paper (see Ref. 10 for a review), we recommend reporting information for each chromophore regardless of these decisions for transparency,^27^ allowing the reader to better understand how the results map onto the expected hemodynamic response. At a larger scale, reaching a chromophore data reporting consensus should improve results interpretations and study comparison efforts in the fNIRS research field.

## 5 Conclusion

fNIRS research has advanced our understanding of brain function in a relatively short period of time. Yet, there are growing concerns regarding inconsistent chromophore data reporting practices to identify neural activation. Our systematic review of recent fNIRS studies confirmed that various approaches are being performed that differentially operationalize neural activation. Strikingly, approximately half of the fNIRS field only reported one chromophore (HbO), many without providing a justification. This practice raises several concerns, because presenting information on only one chromophore limits the ability to interpret how neural activation can be identified with fNIRS. In light of these findings, there is a clear need for a more standardized chromophore data reporting practice in the fNIRS community. To this goal, we provide a general recommendation to report both chromophores, as this practice is grounded in the neurovascular coupling process.

## Code, Data, and Materials Availability

Lists of the 2015, 2018, and 2021 fNIRS articles reviewed to obtain our results can be found in the Supplementary Material.

## References

[1] Boas D. A. et al., “Twenty years of functional near-infrared spectroscopy: introduction for the special issue,” NeuroImage 85, 1–5 (2014). NEIMEF 1053-8119 10.1016/j.neuroimage.2013.11.033 24321364

[r2] Villringer A. et al., “Near infrared spectroscopy (NIRS): a new tool to study hemodynamic changes during activation of brain function in human adults,” Neurosci. Lett. 154(1–2), 101–104 (1993). NELED5 0304-3940 10.1016/0304-3940(93)90181-J 8361619

[3] Villringer A. Chance B. “Non-invasive optical spectroscopy and imaging of human brain function,” Trends Neurosci. 20(10), 435–442 (1997). TNSCDR 0166-2236 10.1016/S0166-2236(97)01132-6 9347608

[4] Pinti P. et al., “The present and future use of functional near‐infrared spectroscopy (fNIRS) for cognitive neuroscience,” Ann. N. Y. Acad. Sci. 1464(1), 5–29 (2020). ANYAA9 0077-8923 10.1111/nyas.13948 30085354 PMC6367070

[5] Lloyd-Fox S. Blasi A. Elwell C. E., “Illuminating the developing brain: the past, present and future of functional near infrared spectroscopy,” Neurosci. Biobehav. Rev. 34(3), 269–284. 10.1016/j.neubiorev.2009.07.008 19632270

[6] Vanderwert R. E. Nelson C. A., “The use of near-infrared spectroscopy in the study of typical and atypical development,” NeuroImage 85, 264–271 (2014). NEIMEF 1053-8119 10.1016/j.neuroimage.2013.10.009 24128733 PMC3910372

[7] Yücel M. A. et al., “Best practices for fNIRS publications,” Neurophotonics 8(1), 012101 (2021). 10.1117/1.NPh.8.1.012101 33442557 PMC7793571

[8] Gemignani J. Gervain J., “Comparing different pre-processing routines for infant fNIRS data,” Dev. Cognit. Neurosci. 48, 100943 (2021). 10.1016/j.dcn.2021.100943 33735718 PMC7985709

[r9] Pfeifer M. D. Scholkmann F. Labruyère R., “Signal processing in functional near-infrared spectroscopy (fNIRS): methodological differences lead to different statistical results,” Front. Hum. Neurosci. 11, 641 (2018). 10.3389/fnhum.2017.00641 29358912 PMC5766679

[10] Pinti P. et al., “Current status and issues regarding pre-processing of fNIRS neuroimaging data: an investigation of diverse signal filtering methods within a general linear model framework,” Front. Hum. Neurosci. 12, 505 (2019). 10.3389/fnhum.2018.00505 30687038 PMC6336925

[11] Hocke P. et al., “Automated processing of fNIRS data—a visual guide to the pitfalls and consequences,” Algorithms 11(5), 67 (2018).1748-7188 10.3390/a11050067 30906511 PMC6428450

[12] Zimeo Morais G. A. et al., “Non-neuronal evoked and spontaneous hemodynamic changes in the anterior temporal region of the human head may lead to misinterpretations of functional near-infrared spectroscopy signals,” Neurophotonics 5(1), 011002 (2018). 10.1117/1.NPh.5.1.011002 28840166 PMC5566266

[13] Bonner R. F. et al., “Model for photon migration in turbid biological media,” J. Opt. Soc. Am. A 4(3), 423 (1987). JOAOD6 0740-3232 10.1364/JOSAA.4.000423 3572576

[14] Attwell D. Iadecola C., “The neural basis of functional brain imaging signals,” Trends Neurosci. 25(12), 621–625 (2002). TNSCDR 0166-2236 10.1016/S0166-2236(02)02264-6 12446129

[15] Friston K. J. et al., “Nonlinear event-related responses in fMRI,” Magn. Reson. Med. 39(1), 41–52 (1998). MRMEEN 0740-3194 10.1002/mrm.1910390109 9438436

[16] Heeger D. J. Ress D., “What does fMRI tell us about neuronal activity?” Nat. Rev. Neurosci. 3(2), 142–151 (2002). NRNAAN 1471-003X 10.1038/nrn730 11836522

[17] Cui X. Bray S. Reiss A. L., “Functional near-infrared spectroscopy (NIRS) signal improvement based on negative correlation between oxygenated and deoxygenated hemoglobin dynamics,” NeuroImage 49(4), 3039–3046 (2010). NEIMEF 1053-8119 10.1016/j.neuroimage.2009.11.050 19945536 PMC2818571

[18] Hoshi Y. Kobayashi N. Tamura M., “Interpretation of near-infrared spectroscopy signals: a study with a newly developed perfused rat brain model,” J. Appl. Physiol. 90(5), 1657–1662 (2001). 10.1152/jappl.2001.90.5.1657 11299252

[r19] Hoshi Y., “Functional near-infrared spectroscopy: current status and future prospects,” J. Biomed. Opt. 12(6), 062106 (2007). JBOPFO 1083-3668 10.1117/1.2804911 18163809

[20] Strangman G. et al., “A quantitative comparison of simultaneous BOLD fMRI and NIRS recordings during functional brain activation,” NeuroImage 17(2), 719–731 (2002). NEIMEF 1053-8119 10.1006/nimg.2002.1227 12377147

[21] Suzuki M. et al., “Prefrontal and premotor cortices are involved in adapting walking and running speed on the treadmill: an optical imaging study,” NeuroImage 23(3), 1020–1026 (2004). NEIMEF 1053-8119 10.1016/j.neuroimage.2004.07.002 15528102

[22] Plichta M. M. et al., “Event-related functional near-infrared spectroscopy (fNIRS): are the measurements reliable?” NeuroImage 31(1), 116–124 (2006). NEIMEF 1053-8119 10.1016/j.neuroimage.2005.12.008 16446104

[23] Huppert T. J. et al., “A temporal comparison of BOLD, ASL, and NIRS hemodynamic responses to motor stimuli in adult humans,” NeuroImage 29(2), 368–382 (2006). NEIMEF 1053-8119 10.1016/j.neuroimage.2005.08.065 16303317 PMC2692693

[24] Cui X. et al., “A quantitative comparison of NIRS and fMRI across multiple cognitive tasks,” NeuroImage 54(4), 2808–2821 (2011). NEIMEF 1053-8119 10.1016/j.neuroimage.2010.10.069 21047559 PMC3021967

[25] Boas D. A. Dale A. M. Franceschini M. A., “Diffuse optical imaging of brain activation: approaches to optimizing image sensitivity, resolution, and accuracy,” NeuroImage 23, S275–S288 (2004). NEIMEF 1053-8119 10.1016/j.neuroimage.2004.07.011 15501097

[26] Volkening N. et al., “Characterizing the influence of muscle activity in fNIRS brain activation measurements,” IFAC-PapersOnLine 49(11), 84–88 (2016). 10.1016/j.ifacol.2016.08.013

[27] Open Science Collaboration, “Estimating the reproducibility of psychological science,” Science 349(6251), aac4716 (2015). SCIEAS 0036-8075 10.1126/science.aac4716 26315443

