# Supplementary material for: Systematic review of fNIRS studies reveals inconsistent chromophore data reporting practices
Source: Neurophotonics. 2022 Dec 23;9(4):040601. doi: 10.1117/1.NPh.9.4.040601 (PMC9780687; doi:10.1117/1.NPh.9.4.040601)
Supplement: Supplementary file 1 [file NPh_009_040601_SD001.pdf]

Systematic Review of fNIRS Studies Reveals Inconsistent Chromophore Data Reporting Practices

Supplementary Material

List of Reviewed fNIRS Studies (2015)

Balconi, M., & Cortesi, L. (2015). Brain activity (fNIRS) in control state differs from the execution and observation of object-related and object-unrelated actions. *Journal of Motor Behavior*, *48*(4), 289–296. https://doi.org/10.1080/00222895.2015.1092936

Balconi, M., Grippa, E., & Vanutelli, M. E. (2015). What hemodynamic (fNIRS), electrophysiological (EEG) and autonomic integrated measures can tell us about emotional processing. *Brain and Cognition*, *95*, 67–76. https://doi.org/10.1016/j.bandc.2015.02.001

Balconi, M., Vanutelli, M. E., Bartolo, A., & Cortesi, L. (2015). Transitive and intransitive gesture execution and observation compared to resting state: The hemodynamic measures (fNIRS). *Cognitive Processing*, *16*(S1), 125–129. https://doi.org/10.1007/s10339-015-0729-2

Bhutta, M. R., Hong, M. J., Kim, Y.-H., & Hong, K.-S. (2015). Single-trial lie detection using a combined fNIRS-polygraph system. *Frontiers in Psychology*, *6*, 709. https://doi.org/10.3389/fpsyg.2015.00709

Bigliassi, M., Barreto-Silva, V., Altimari, L. R., Vandoni, M., Codrons, E., & Buzzachera, C. F. (2015). How motivational and calm music may affect the prefrontal cortex area and emotional responses: A functional near-infrared spectroscopy (fNIRS) study. *Perceptual and Motor Skills*, *120*(1), 202–218. https://doi.org/10.2466/27.24.PMS.120v12x5

Bigliassi, M., León-Domínguez, U., & Altimari, L. R. (2015). How does the prefrontal cortex "listen" to classical and techno music? A functional near-infrared spectroscopy (fNIRS) study. *Psychology & Neuroscience*, *8*(2), 246–256. https://doi.org/10.1037/h0101064

Bolling, D. Z., Pelphrey, K. A., & Kaiser, M. D. (2015). Social inclusion enhances biological motion processing: A functional near-infrared spectroscopy study. *Brain Topography*, *28*(1), 184–185. https://doi.org/10.1007/s10548-014-0419-x

Bowman, L. C., Kovelman, I., Hu, X., & Wellman, H. M. (2015). Children's belief- and desire-reasoning in the temporoparietal junction: Evidence for specialization from functional near-infrared spectroscopy. *Frontiers in Human Neuroscience*, *9*, 560. https://doi.org/10.3389/fnhum.2015.00560

Boyer, M., Cummings, M. L., Spence, L. B., & Solovey, E. T. (2015). Investigating mental workload changes in a long duration supervisory control task. *Interacting with Computers*, *27*(5), 512–520. https://doi.org/10.1093/iwc/iwv012

Brucker, B., Ehlis, A.-C., Häußinger, F. B., Fallgatter, A. J., & Gerjets, P. (2015). Watching corresponding gestures facilitates learning with animations by activating human mirror-neurons: An fNIRS study. *Learning and Instruction*, *36*, 27–37. https://doi.org/10.1016/j.learninstruc.2014.11.003

Brunetti, M., Morkisch, N., Fritzsch, C., Mehnert, J., Steinbrink, J., Niedeggen, M., & Dohle, C. (2015). Potential determinants of efficacy of mirror therapy in stroke patients – A pilot study. *Restorative Neurology and Neuroscience*, *33*(4), 421–434. https://doi.org/10.3233/RNN-140421

Cao, J., Khan, B., Hervey, N., Tian, F., Delgado, M. R., Clegg, N. J., Smith, L., Roberts, H., Tulchin-Francis, K., Shierk, A., Shagman, L., MacFarlane, D., Liu, H., & Alexandrakis,

G. (2015). Evaluation of cortical plasticity in children with cerebral palsy undergoing constraint-induced movement therapy based on functional near-infrared spectroscopy. *Journal of Biomedical Optics*, *20*(4), 046009. https://doi.org/10.1117/1.JBO.20.4.046009

Chen, L.-C., Sandmann, P., Thorne, J. D., Bleichner, M. G., & Debener, S. (2016). Cross-modal functional reorganization of visual and auditory cortex in adult cochlear implant users identified with fNIRS. *Neural Plasticity*, *2015*, 1–13. https://doi.org/10.1155/2016/4382656

Chen, L.-C., Sandmann, P., Thorne, J. D., Herrmann, C. S., & Debener, S. (2015). Association of concurrent fNIRS and EEG signatures in response to auditory and visual stimuli. *Brain Topography*, *28*(5), 710–725. https://doi.org/10.1007/s10548-015-0424-8

Cheng, X., Li, X., & Hu, Y. (2015). Synchronous brain activity during cooperative exchange depends on gender of partner: A fNIRS-based hyperscanning study: Synchronous Brain Activities. *Human Brain Mapping*, *36*(6), 2039–2048. https://doi.org/10.1002/hbm.22754

Cui, X., Baker, J. M., Liu, N., & Reiss, A. L. (2015). Sensitivity of fNIRS measurement to head motion: An applied use of smartphones in the lab. *Journal of Neuroscience Methods*, *245*, 37–43. https://doi.org/10.1016/j.jneumeth.2015.02.006

Dalmis, M. U., & Akin, A. (2015). Similarity analysis of functional connectivity with functional near-infrared spectroscopy. *Journal of Biomedical Optics*, *20*(8), 086012. https://doi.org/10.1117/1.JBO.20.8.086012

Deepeshwar, S., Vinchurkar, S. A., Visweswaraiah, N. K., & Nagendra, H. R. (2015). Hemodynamic responses on prefrontal cortex related to meditation and attentional task. *Frontiers in Systems Neuroscience*, *8*, 252. https://doi.org/10.3389/fnsys.2014.00252

Dewey, R. S., & Hartley, D. E. H. (2015). Cortical cross-modal plasticity following deafness measured using functional near-infrared spectroscopy. *Hearing Research*, *325*, 55–63. https://doi.org/10.1016/j.heares.2015.03.007

Duan, L., Dai, R.-N., Xiao, X., Sun, P.-P., Li, Z., & Zhu, C.-Z. (2015). Cluster imaging of multi-brain networks (CIMBN): A general framework for hyperscanning and modeling a group of interacting brains. *Frontiers in Neuroscience*, *9*, 267. https://doi.org/10.3389/fnins.2015.00267

Durantin, G., Dehais, F., & Delorme, A. (2015). Characterization of mind wandering using fNIRS. *Frontiers in Systems Neuroscience*, *9*, 45. https://doi.org/10.3389/fnsys.2015.00045

Durantin, G., Scannella, S., Gateau, T., Delorme, A., & Dehais, F. (2015). Processing functional near infrared spectroscopy signal with a kalman filter to assess working memory during simulated flight. *Frontiers in Human Neuroscience*, *9*, 707. https://doi.org/10.3389/fnhum.2015.00707

Edwards, L. A., Wagner, J. B., Simon, C. E., & Hyde, D. C. (2015). Functional brain organization for number processing in pre-verbal infants. *Developmental Science*, *19*(5), 757–769. https://doi.org/10.1111/desc.12333

Egashira, K., Matsuo, K., Nakashima, M., Watanuki, T., Harada, K., Nakano, M., Matsubara, T., Takahashi, K., & Watanabe, Y. (2015). Blunted brain activation in patients with schizophrenia in response to emotional cognitive inhibition: A functional near-infrared spectroscopy study. *Schizophrenia Research*, *162*(1–3), 196–204. https://doi.org/10.1016/j.schres.2014.12.038

Ehlis, A.-C., Haeussinger, F. B., Gastel, A., Fallgatter, A. J., & Plewnia, C. (2015). Task-dependent and polarity-specific effects of prefrontal transcranial direct current stimulation on cortical activation during word fluency. *NeuroImage*, *140*, 134–140. https://doi.org/10.1016/j.neuroimage.2015.12.047

Emberson, L. L., Richards, J. E., & Aslin, R. N. (2015). Top-down modulation in the infant brain: Learning-induced expectations rapidly affect the sensory cortex at 6 months. *Proceedings of the National Academy of Sciences*, *112*(31), 9585–9590. https://doi.org/10.1073/pnas.1510343112

Ferreri, L., Bigand, E., Bard, P., & Bugaiska, A. (2015). The influence of music on prefrontal cortex during episodic encoding and retrieval of verbal information: A multichannel fNIRS study. *Behavioural Neurology*, *2015*, 1–11. https://doi.org/10.1155/2015/707625

Gateau, T., Durantin, G., Lancelot, F., Scannella, S., & Dehais, F. (2015). Real-time state estimation in a flight simulator using fNIRS. *PLOS ONE*, *10*(3), e0121279. https://doi.org/10.1371/journal.pone.0121279

Grant, H., Bhambhani, Y., & Singhal, A. (2015). Hemodynamic changes in the prefrontal cortex during working memory in essential hypertension. *Journal of the American Society of Hypertension*, *9*(8), 628–639. https://doi.org/10.1016/j.jash.2015.06.007

Guhn, A., Domschke, K., Müller, L. D., Dresler, T., Eff, F., Kopf, J., Deckert, J., Reif, A., & Herrmann, M. J. (2015). Neuropeptide S receptor gene variation and neural correlates of cognitive emotion regulation. *Social Cognitive and Affective Neuroscience*, *10*(12), 1730–1737. https://doi.org/10.1093/scan/nsv061

Harmat, L., de Manzano, Ö., Theorell, T., Högman, L., Fischer, H., & Ullén, F. (2015). Physiological correlates of the flow experience during computer game playing.

International Journal of Psychophysiology, *97*(1), 1–7. https://doi.org/10.1016/j.ijpsycho.2015.05.001

Heinzel, S., Metzger, F. G., Ehlis, A.-C., Korell, R., Alboji, A., Haeussinger, F. B., Wurster, I., Brockmann, K., Suenkel, U., Eschweiler, G. W., Maetzler, W., Berg, D., & Fallgatter, A. J. (2015). Age and vascular burden determinants of cortical hemodynamics underlying verbal fluency. *PLOS ONE*, *10*(9), e0138863. https://doi.org/10.1371/journal.pone.0138863

Helmich, I., Holle, H., Rein, R., & Lausberg, H. (2015). Brain oxygenation patterns during the execution of tool use demonstration, tool use pantomime, and body-part-as-object tool use. *International Journal of Psychophysiology*, *96*(1), 1–7. https://doi.org/10.1016/j.ijpsycho.2015.03.001

Himichi, T., Fujita, H., & Nomura, M. (2015). Negative emotions impact lateral prefrontal cortex activation during theory of mind: An fNIRS study. *Social Neuroscience*, *10*(6), 605–615. https://doi.org/10.1080/17470919.2015.1017112

Himichi, T., & Nomura, M. (2015). Modulation of empathy in the left ventrolateral prefrontal cortex facilitates altruistic behavior: An fNIRS study. *Journal of Integrative Neuroscience*, *14*(2), 207–222. https://doi.org/10.1142/S0219635215500120

Holper, L., Aleksandrowicz, A., Müller, M., Ajdacic-Gross, V., Haker, H., Fallgatter, A. J., Hagenmuller, F., Rössler, W., & Kawohl, W. (2015). Brain correlates of verbal fluency in subthreshold psychosis assessed by functional near-infrared spectroscopy. *Schizophrenia Research*, *168*(1–2), 23–29. https://doi.org/10.1016/j.schres.2015.07.043

Holper, L., Scholkmann, F., & Seifritz, E. (2015). Time–frequency dynamics of the sum of intra- and extracerebral hemodynamic functional connectivity during resting-state and

respiratory challenges assessed by multimodal functional near-infrared spectroscopy. *NeuroImage*, *120*, 481–492. https://doi.org/10.1016/j.neuroimage.2015.07.021

Holtzer, R., Mahoney, J. R., Izzetoglu, M., Wang, C., England, S., & Verghese, J. (2015). Online fronto-cortical control of simple and attention-demanding locomotion in humans. *NeuroImage*, *112*, 152–159. https://doi.org/10.1016/j.neuroimage.2015.03.002

Holtzer, R., Verghese, J., Allali, G., Izzetoglu, M., Wang, C., & Mahoney, J. R. (2015). Neurological gait abnormalities moderate the functional brain signature of the posture first hypothesis. *Brain Topography*, *29*(2), 334–343. https://doi.org/10.1007/s10548-015-0465-z

Hong, K.-S., Naseer, N., & Kim, Y.-H. (2015). Classification of prefrontal and motor cortex signals for three-class fNIRS–BCI. *Neuroscience Letters*, *587*, 87–92. https://doi.org/10.1016/j.neulet.2014.12.029

Huang, F., Hirano, D., Shi, Y., & Taniguchi, T. (2015). Comparison of cortical activation in an upper limb added-purpose task versus a single-purpose task: A near-infrared spectroscopy study. *Journal of Physical Therapy Science*, *27*(12), 3891–3894. https://doi.org/10.1589/jpts.27.3891

Huang, J., Wang, F., Ding, Y., Niu, H., Tian, F., Liu, H., & Song, Y. (2015). Predicting N2pc from anticipatory HbO activity during sustained visuospatial attention: A concurrent fNIRS–ERP study. *NeuroImage*, *113*, 225–234. https://doi.org/10.1016/j.neuroimage.2015.03.044

Huhn, A. S., Meyer, R. E., Harris, J. D., Ayaz, H., Deneke, E., Stankoski, D. M., & Bunce, S. C. (2015). Evidence of anhedonia and differential reward processing in prefrontal cortex

among post-withdrawal patients with prescription opiate dependence. *Brain Research Bulletin*, *123*, 102–109. https://doi.org/10.1016/j.brainresbull.2015.12.004

Hyde, D. C., Aparicio Betancourt, M., & Simon, C. E. (2015). Human temporal-parietal junction spontaneously tracks others' beliefs: A functional near-infrared spectroscopy study. *Human Brain Mapping*, *36*(12), 4831–4846. https://doi.org/10.1002/hbm.22953

Inamoto, K., Sakuma, S., Ariji, Y., Higuchi, N., Izumi, M., & Nakata, K. (2015). Measurement of cerebral blood volume dynamics during volitional swallowing using functional near-infrared spectroscopy: An exploratory study. *Neuroscience Letters*, *588*, 67–71. https://doi.org/10.1016/j.neulet.2014.12.034

Jiang, J., Chen, C., Dai, B., Shi, G., Ding, G., Liu, L., & Lu, C. (2015). Leader emergence through interpersonal neural synchronization. *Proceedings of the National Academy of Sciences*, *112*(14), 4274–4279. https://doi.org/10.1073/pnas.1422930112

Jones, K. T., Gözenman, F., & Berryhill, M. E. (2015). The strategy and motivational influences on the beneficial effect of neurostimulation: A tDCS and fNIRS study. *NeuroImage*, *105*, 238–247. https://doi.org/10.1016/j.neuroimage.2014.11.012

Kreplin, U., & Fairclough, S. H. (2015). Effects of self-directed and other-directed introspection and emotional valence on activation of the rostral prefrontal cortex during aesthetic experience. *Neuropsychologia*, *71*, 38–45. https://doi.org/10.1016/j.neuropsychologia.2015.03.013

Kroczek, A. M., Haeussinger, F. B., Fallgatter, A. J., Batra, A., & Ehlis, A.-C. (2015). Prefrontal functional connectivity measured with near-infrared spectroscopy during smoking cue exposure: Cue exposure hemodynamics. *Addiction Biology*, *22*(2), 513–522. https://doi.org/10.1111/adb.12344

Laguë-Beauvais, M., Fraser, S. A., Desjardins-Crépeau, L., Castonguay, N., Desjardins, M., Lesage, F., & Bherer, L. (2015). Shedding light on the effect of priority instructions during dual-task performance in younger and older adults: A fNIRS study. *Brain and Cognition*, *98*, 1–14. https://doi.org/10.1016/j.bandc.2015.05.001

León-Domínguez, U., Martín-Rodríguez, J. F., & León-Carrión, J. (2015). Executive n-back tasks for the neuropsychological assessment of working memory. *Behavioural Brain Research*, *292*, 167–173. https://doi.org/10.1016/j.bbr.2015.06.002

Li, F., Zhu, H., Gao, Q., Xu, G., Li, X., Hu, Z., & He, S. (2015). Using functional near-infrared spectroscopy (fNIRS) to detect the prefrontal cortical responses to deception under different motivations. *Biomedical Optics Express*, *6*(9), 3503. https://doi.org/10.1364/BOE.6.003503

Li, L., Zeng, L., Lin, Z.-J., Cazzell, M., & Liu, H. (2015). Tutorial on use of intraclass correlation coefficients for assessing intertest reliability and its application in functional near-infrared spectroscopy–based brain imaging. *Journal of Biomedical Optics*, *20*(5), 050801. https://doi.org/10.1117/1.JBO.20.5.050801

Lloyd-Fox, S., Széplaki-Köllőd, B., Yin, J., & Csibra, G. (2015). Are you talking to me? Neural activations in 6-month-old infants in response to being addressed during natural interactions. *Cortex*, *70*, 35–48. https://doi.org/10.1016/j.cortex.2015.02.005

Lloyd-Fox, S., Wu, R., Richards, J. E., Elwell, C. E., & Johnson, M. H. (2015). Cortical activation to action perception is associated with action production abilities in young infants. *Cerebral Cortex*, *25*(2), 289–297. https://doi.org/10.1093/cercor/bht207

Mahoney, J. R., Holtzer, R., Izzetoglu, M., Zemon, V., Verghese, J., & Allali, G. (2015). The role of prefrontal cortex during postural control in Parkinsonian syndromes a functional

near-infrared spectroscopy study. *Brain Research*, *1633*, 126–138. https://doi.org/10.1016/j.brainres.2015.10.053

Maidan, I., Bernad-Elazari, H., Gazit, E., Giladi, N., Hausdorff, J. M., & Mirelman, A. (2015). Changes in oxygenated hemoglobin link freezing of gait to frontal activation in patients with Parkinson disease: An fNIRS study of transient motor-cognitive failures. *Journal of Neurology*, *262*(4), 899–908. https://doi.org/10.1007/s00415-015-7650-6

Marx, A.-M., Ehlis, A.-C., Furdea, A., Holtmann, M., Banaschewski, T., Brandeis, D., Rothenberger, A., Gevensleben, H., Freitag, C. M., Fuchsenberger, Y., Fallgatter, A. J., & Strehl, U. (2015). Near-infrared spectroscopy (NIRS) neurofeedback as a treatment for children with attention deficit hyperactivity disorder (ADHD)—A pilot study. *Frontiers in Human Neuroscience*, *8*, 1038. https://doi.org/10.3389/fnhum.2014.01038

Matsuo, K., Ban, R., Hama, Y., & Yuzuriha, S. (2015). Eyelid opening with trigeminal proprioceptive activation regulates a brainstem arousal mechanism. *PLOS ONE*, *10*(8), e0134659. https://doi.org/10.1371/journal.pone.0134659

McKendrick, R., Parasuraman, R., & Ayaz, H. (2015). Wearable functional near infrared spectroscopy (fNIRS) and transcranial direct current stimulation (tDCS): Expanding vistas for neurocognitive augmentation. *Frontiers in Systems Neuroscience*, *9*, 27. https://doi.org/10.3389/fnsys.2015.00027

Mehta, R. K. (2015). Stunted PFC activity during neuromuscular control under stress with obesity. *European Journal of Applied Physiology*, *116*(2), 319–326. https://doi.org/10.1007/s00421-015-3283-9

Mehta, R., Shortz, A., & Benden, M. (2015). Standing up for learning: A pilot investigation on the neurocognitive benefits of stand-biased school desks. *International Journal of*

Environmental Research and Public Health, *13*(1), 59. https://doi.org/10.3390/ijerph13010059

Metzger, F. G., Ehlis, A.-C., Haeussinger, F. B., Fallgatter, A. J., & Hagen, K. (2015). Effects of cholinesterase inhibitor on brain activation in Alzheimer's patients measured with functional near-infrared spectroscopy. *Psychopharmacology*, *232*(23), 4383–4391. https://doi.org/10.1007/s00213-015-4066-z

Miura, S., Kobayashi, Y., Kawamura, K., Nakashima, Y., & Fujie, M. G. (2015). Brain activation in parietal area during manipulation with a surgical robot simulator. *International Journal of Computer Assisted Radiology and Surgery*, *10*(6), 783–790. https://doi.org/10.1007/s11548-015-1178-1

Monden, Y., Dan, I., Nagashima, M., Dan, H., Uga, M., Ikeda, T., Tsuzuki, D., Kyutoku, Y., Gunji, Y., Hirano, D., Taniguchi, T., Shimoizumi, H., Watanabe, E., & Yamagata, T. (2015). Individual classification of ADHD children by right prefrontal hemodynamic responses during a go/no-go task as assessed by fNIRS. *NeuroImage: Clinical*, *9*, 1–12. https://doi.org/10.1016/j.nicl.2015.06.011

Nishiyori, R., Bisconti, S., & Ulrich, B. (2015). Motor cortex activity during functional motor skills: An fNIRS study. *Brain Topography*, *29*(1), 42–55. https://doi.org/10.1007/s10548-015-0443-5

Oka, N., Yoshino, K., Yamamoto, K., Takahashi, H., Li, S., Sugimachi, T., Nakano, K., Suda, Y., & Kato, T. (2015). Greater activity in the frontal cortex on left curves: A vector-based fNIRS Study of left and right curve driving. *PLOS ONE*, *10*(5), e0127594. https://doi.org/10.1371/journal.pone.0127594

Ono, Y., Noah, J. A., Zhang, X., Nomoto, Y., Suzuki, T., Shimada, S., Tachibana, A., Bronner, S., & Hirsch, J. (2015). Motor learning and modulation of prefrontal cortex: An fNIRS assessment. *Journal of Neural Engineering*, *12*(6), 066004. https://doi.org/10.1088/1741-2560/12/6/066004

Osaka, N., Minamoto, T., Yaoi, K., Azuma, M., Shimada, Y. M., & Osaka, M. (2015). How two brains make one synchronized mind in the inferior frontal cortex: FNIRS-based hyperscanning during cooperative singing. *Frontiers in Psychology*, *6*. https://doi.org/10.3389/fpsyg.2015.01811

Otsuka, T., Yamasaki, R., Shimazaki, T., Yoshino, F., Sasaguri, K., & Kawata, T. (2015). Effects of mandibular retrusive deviation on prefrontal cortex activation: A functional near-infrared spectroscopy study. *BioMed Research International*, *2015*, 1–6. https://doi.org/10.1155/2015/373769

Perlman, S. B., Huppert, T. J., & Luna, B. (2015). Functional near-infrared spectroscopy evidence for development of prefrontal engagement in working memory in early through middle childhood. *Cerebral Cortex*, *26*(6), 2790–2799. https://doi.org/10.1093/cercor/bhv139

Pinti, P., Cardone, D., & Merla, A. (2015). Simultaneous fNIRS and thermal infrared imaging during cognitive task reveal autonomic correlates of prefrontal cortex activity. *Scientific Reports*, *5*(1), 17471. https://doi.org/10.1038/srep17471

Qiu, L., Zhang, X., & Li, J. (2015). Optical imaging of the prefrontal activity in joint attention experience. *Biomedical Optics Express*, *6*(9), 3437–3448. https://doi.org/10.1364/BOE.6.003437

Ravicz, M. M., Perdue, K. L., Westerlund, A., Vanderwert, R. E., & Nelson, C. A. (2015). Infants' neural responses to facial emotion in the prefrontal cortex are correlated with temperament: A functional near-infrared spectroscopy study. *Frontiers in Psychology*, *6*, 922. https://doi.org/10.3389/fpsyg.2015.00922

Roberts, C. A., & Montgomery, C. (2015). FNIRS suggests increased effort during executive access in ecstasy polydrug users. *Psychopharmacology*, *232*(9), 1571–1582. https://doi.org/10.1007/s00213-014-3795-8

Roberts, C. A., Wetherell, M. A., Fisk, J. E., & Montgomery, C. (2015). Differences in prefrontal blood oxygenation during an acute multitasking stressor in ecstasy polydrug users. *Psychological Medicine*, *45*(2), 395–406. https://doi.org/10.1017/S0033291714001500

Roberts, C., & Montgomery, C. (2015). Cortical oxygenation suggests increased effort during cognitive inhibition in ecstasy polydrug users. *Journal of Psychopharmacology*, *29*(11), 1170–1181. https://doi.org/10.1177/0269881115598412

Sangani, S., Lamontagne, A., & Fung, J. (2015). Cortical mechanisms underlying sensorimotor enhancement promoted by walking with haptic inputs in a virtual environment. *Progress in Brain Research*, *218*, 313–330. https://doi.org/10.1016/bs.pbr.2014.12.003

Schneider, S., Wagels, L., Haeussinger, F. B., Fallgatter, A. J., Ehlis, A.-C., & Rapp, A. M. (2015). Haemodynamic and electrophysiological markers of pragmatic language comprehension in schizophrenia. *The World Journal of Biological Psychiatry*, *16*(6), 398–410. https://doi.org/10.3109/15622975.2015.1019359

Shidoh, S., Akiyama, T., Horiguchi, T., Ohira, T., & Yoshida, K. (2015). The process of change in hemodynamics after revascularization in the ischemic brain. *NeuroReport*, *26*(11), 629–633. https://doi.org/10.1097/WNR.0000000000000400

Shortz, A. E., Pickens, A., Zheng, Q., & Mehta, R. K. (2015). The effect of cognitive fatigue on prefrontal cortex correlates of neuromuscular fatigue in older women. *Journal of NeuroEngineering and Rehabilitation*, *12*(1), 115. https://doi.org/10.1186/s12984-015-0108-3

Stojanovic-Radic, J., Wylie, G., Voelbel, G., Chiaravalloti, N., & DeLuca, J. (2015). Neuroimaging and cognition using functional near infrared spectroscopy (fNIRS) in multiple sclerosis. *Brain Imaging and Behavior*, *9*(2), 302–311. https://doi.org/10.1007/s11682-014-9307-y

Sugawara, K., Onishi, H., Tsubaki, A., Takai, H., Tokunaga, Y., & Tamaki, H. (2015). Regional changes in cerebral oxygenation during repeated passive movement measured by functional near-infrared spectroscopy. *Frontiers in Human Neuroscience*, *9*, 641. https://doi.org/10.3389/fnhum.2015.00641

Sugiura, L., Ojima, S., Matsuba-Kurita, H., Dan, I., Tsuzuki, D., Katura, T., & Hagiwara, H. (2015). Effects of sex and proficiency in second language processing as revealed by a large-scale fNIRS study of school-aged children: Proficiency and Sex Affect L2 Processing. *Human Brain Mapping*, *36*(10), 3890–3911. https://doi.org/10.1002/hbm.22885

Tang, H., Mai, X., Wang, S., Zhu, C., Krueger, F., & Liu, C. (2015). Interpersonal brain synchronization in the right temporo-parietal junction during face-to-face economic exchange. *Social Cognitive and Affective Neuroscience*, *11*(1), 23–32. https://doi.org/10.1093/scan/nsv092

Üçeyler, N., Zeller, J., Kewenig, S., Kittel-Schneider, S., Fallgatter, A. J., & Sommer, C. (2015). Increased cortical activation upon painful stimulation in fibromyalgia syndrome. *BMC Neurology*, *15*(1), 210. https://doi.org/10.1186/s12883-015-0472-4

Urakawa, S., Takamoto, K., Ishikawa, A., Ono, T., & Nishijo, H. (2015). Selective medial prefrontal cortex responses during live mutual gaze interactions in human infants: An fNIRS study. *Brain Topography*, *28*(5), 691–701. https://doi.org/10.1007/s10548-014-0414-2

Urban, K. J., Barlow, K. M., Jimenez, J. J., Goodyear, B. G., & Dunn, J. F. (2015). Functional near-infrared spectroscopy reveals reduced interhemispheric cortical communication after pediatric concussion. *Journal of Neurotrauma*, *32*(11), 833–840. https://doi.org/10.1089/neu.2014.3577

Vanutelli, M. E., & Balconi, M. (2015). Perceiving emotions in human–human and human–animal interactions: Hemodynamic prefrontal activity (fNIRS) and empathic concern. *Neuroscience Letters*, *605*, 1–6. https://doi.org/10.1016/j.neulet.2015.07.020

Vanutelli, M. E., Cortesi, L., Molteni, E., & Balconi, M. (2015). fNIRS measure of transitive and intransitive gesture execution, observation and imagination in ecological setting: A pilot study. *IEEE*, 3484–3487. https://doi.org/10.1109/EMBC.2015.7319143

Venclove, S., Daktariunas, A., & Ruksenas, O. (2015). Functional near-infrared spectroscopy: A continuous wave type based system for human frontal lobe studies. *EXCLI Journal*, *14*, 1145. https://doi.org/10.17179/EXCLI2015-614

Visani, E., Canafoglia, L., Gilioli, I., Sebastiano, D. R., Contarino, V. E., Duran, D., Panzica, F., Cubeddu, R., Contini, D., Zucchelli, L., Spinelli, L., Caffini, M., Molteni, E., Bianchi, A. M., Cerutti, S., Franceschetti, S., & Torricelli, A. (2015). Hemodynamic and EEG time-

courses during unilateral hand movement in patients with cortical myoclonus. An EEG-fMRI and EEG-TD-fNIRS study. *Brain Topography*, *28*(6), 915–925. https://doi.org/10.1007/s10548-014-0402-6

Watanuki, T., Matsuo, K., Egashira, K., Nakashima, M., Harada, K., Nakano, M., Matsubara, T., Takahashi, K., & Watanabe, Y. (2015). Precentral and inferior prefrontal hypoactivation during facial emotion recognition in patients with schizophrenia: A functional near-infrared spectroscopy study. *Schizophrenia Research*, *170*(1), 109–114. https://doi.org/10.1016/j.schres.2015.11.012

Wiggins, I. M., & Hartley, D. E. H. (2015). A synchrony-dependent influence of sounds on activity in visual cortex measured using functional near-infrared spectroscopy (fNIRS). *PLOS ONE*, *10*(3), e0122862. https://doi.org/10.1371/journal.pone.0122862

Wilcox, T., & Biondi, M. (2015). Functional activation in the ventral object processing pathway during the first year. *Frontiers in Systems Neuroscience*, *9*, 180. https://doi.org/10.3389/fnsys.2015.00180

Yamada, T., Umeyama, S., & Ohashi, M. (2015). Removal of motion artifacts originating from optode fluctuations during functional near-infrared spectroscopy measurements. *Biomedical Optics Express*, *6*(12), 4632–4649. https://doi.org/10.1364/BOE.6.004632

Yan, J., Wei, Y., Wang, Y., Xu, G., Li, Z., & Li, X. (2015). Use of functional near-infrared spectroscopy to evaluate the effects of anodal transcranial direct current stimulation on brain connectivity in motor-related cortex. *Journal of Biomedical Optics*, *20*(4), 046007. https://doi.org/10.1117/1.JBO.20.4.046007

Yin, X., Xu, B., Jiang, C., Fu, Y., Wang, Z., Li, H., & Shi, G. (2015). A hybrid BCI based on EEG and fNIRS signals improves the performance of decoding motor imagery of both

force and speed of hand clenching. *Journal of Neural Engineering*, *12*(3), 036004. https://doi.org/10.1088/1741-2560/12/3/036004

Zhao, K., Yan, W.-J., Chen, Y.-H., & Fu, X. (2015). Temporal orienting of attention: An fNIRS study on the illusion of “a watched pot never boils.” *PsyCh Journal*, *4*(2), 47–54. https://doi.org/10.1002/pchj.67

Zhu, H., Li, J., Fan, Y., Li, X., Huang, D., & He, S. (2015). Atypical prefrontal cortical responses to joint/non-joint attention in children with autism spectrum disorder (ASD): A functional near-infrared spectroscopy study. *Biomedical Optics Express*, *6*(3), 690–701. https://doi.org/10.1364/BOE.6.000690

Zinszer, B. D., Chen, P., Wu, H., Shu, H., & Li, P. (2015). Second language experience modulates neural specialization for first language lexical tones. *Journal of Neurolinguistics*, *33*, 50–66. https://doi.org/10.1016/j.jneuroling.2014.09.005

List of Reviewed fNIRS Studies (2018)

Abdullayev, A., Baskak, B., Sedes Baskak, N., Kir, Y., Kale, E., Devrimci Ozguven, H., Baran, Z., & Yenihayat, I. (2018). Prefrontal cortex activity during facial affect processing in schizophrenia: Association with clinical symptoms and social cognitive functions. *Turkish Journal of Psychiatry*, *29*(4), 229–237. https://doi.org/10.5080/u22741

Aitchison, R. T., Ward, L., Kennedy, G. J., Shu, X., Mansfield, D. C., & Shahani, U. (2018). Measuring visual cortical oxygenation in diabetes using functional near-infrared spectroscopy. *Acta Diabetologica*, *55*(11), 1181–1189. https://doi.org/10.1007/s00592-018-1200-5

Altvater-Mackensen, N., & Grossmann, T. (2018). Modality-independent recruitment of inferior frontal cortex during speech processing in human infants. *Developmental Cognitive Neuroscience*, *34*, 130–138. https://doi.org/10.1016/j.dcn.2018.10.002

Al-Yahya, E., Mahmoud, W., Meester, D., Esser, P., & Dawes, H. (2019). Neural substrates of cognitive motor interference during walking; peripheral and central mechanisms. *Frontiers in Human Neuroscience*, *12*, 536. https://doi.org/10.3389/fnhum.2018.00536

Arimitsu, T., Minagawa, Y., Yagihashi, T., O. Uchida, M., Matsuzaki, A., Ikeda, K., & Takahashi, T. (2018). The cerebral hemodynamic response to phonetic changes of speech in preterm and term infants: The impact of postmenstrual age. *NeuroImage: Clinical*, *19*, 599–606. https://doi.org/10.1016/j.nicl.2018.05.005

Arredondo, M. M., Hu, X.-S., Seifert, E., Satterfield, T., & Kovelman, I. (2019). Bilingual exposure enhances left IFG specialization for language in children. *Bilingualism: Language and Cognition*, *22*(04), 783–801. https://doi.org/10.1017/S1366728918000512

Artemenko, C., Coldea, A., Soltanlou, M., Dresler, T., Nuerk, H., & Ehlis, A. (2018). The neural circuits of number and letter copying: An fNIRS study. *Experimental Brain Research*, *236*(4), 1129–1138. https://doi.org/10.1007/s00221-018-5204-8

Artemenko, C., Soltanlou, M., Dresler, T., Ehlis, A., & Nuerk, H. (2018). The neural correlates of arithmetic difficulty depend on mathematical ability: Evidence from combined fNIRS and ERP. *Brain Structure and Function*, *223*(6), 2561–2574. https://doi.org/10.1007/s00429-018-1618-0

Artemenko, C., Soltanlou, M., Ehlis, A.-C., Nuerk, H.-C., & Dresler, T. (2018). The neural correlates of mental arithmetic in adolescents: A longitudinal fNIRS study. *Behavioral and Brain Functions*, *14*(1), 1–13. https://doi.org/10.1186/s12993-018-0137-8

Arun, K., Smitha, K., Rajesh, P., & Kesavadas, C. (2018). Functional near-infrared spectroscopy is in moderate accordance with functional MRI in determining lateralisation of frontal language areas. *The Neuroradiology Journal*, *31*(2), 133–141. https://doi.org/10.1177/1971400917739083

Baker, J. M., Bruno, J. L., Gundran, A., Hosseini, S. M. H., & Reiss, A. L. (2018). FNIRS measurement of cortical activation and functional connectivity during a visuospatial working memory task. *PloS One*, *13*(8), e0201486. https://doi.org/10.1371/journal.pone.0201486

Balada, F., Lucas, I., Blanch, Á., Blanco, E., & Aluja, A. (2019). Neuroticism is associated with reduced oxygenation levels in the lateral prefrontal cortex following exposure to unpleasant images. *Physiology & Behavior*, *199*, 66–72. https://doi.org/10.1016/j.physbeh.2018.11.002

Balconi, M., Cotelli, M., Rossi, R., Rillosi, L., Beneduce, R., Tura, G. B., Brambilla, M., Venturella, I., & Vanutelli, M. E. (2018). Emotion regulation in schizophrenia: A comparison between implicit (EEG and fNIRS) and explicit (valence) measures: Preliminary observations. *Asian Journal of Psychiatry*, *34*, 12–13. https://doi.org/10.1016/j.ajp.2018.03.018

Balconi, M., Frezza, A., & Vanutelli, M. E. (2018). Emotion Regulation in Schizophrenia: A Pilot Clinical Intervention as Assessed by EEG and Optical Imaging (Functional Near-Infrared Spectroscopy). *Frontiers in Human Neuroscience*, *12*, 395. https://doi.org/10.3389/fnhum.2018.00395

Balconi, M., Gatti, L., & Vanutelli, M. E. (2018). When cooperation goes wrong: Brain and behavioural correlates of ineffective joint strategies in dyads. *International Journal of Neuroscience*, *128*(2), 155–166. https://doi.org/10.1080/00207454.2017.1379519

Balconi, M., Siri, C., Meucci, N., Pezzoli, G., & Angioletti, L. (2018). Personality traits and cortical activity affect gambling behavior in parkinson's disease. *Journal of Parkinson's Disease*, *8*(2), 341–352. https://doi.org/10.3233/JPD-171290

Balconi, M., Vanutelli, M. E., & Gatti, L. (2018). Functional brain connectivity when cooperation fails. *Brain and Cognition*, *123*, 65–73. https://doi.org/10.1016/j.bandc.2018.02.009

Bandara, D., Velipasalar, S., Bratt, S., & Hirshfield, L. (2018). Building predictive models of emotion with functional near-infrared spectroscopy. *International Journal of Human-Computer Studies*, *110*, 75–85. https://doi.org/10.1016/j.ijhcs.2017.10.001

Bauernfeind, G., C. Wriessnegger, S., Haumann, S., & Lenarz, T. (2018). Cortical activation patterns to spatially presented pure tone stimuli with different intensities measured by

functional near-infrared spectroscopy. *Human Brain Mapping*, *39*(7), 2710–2724. https://doi.org/10.1002/hbm.24034

Berger, A., Henrik Pixa, N., Steinberg, F., & Doppelmayr, M. (2018). Brain oscillatory and hemodynamic activity in a bimanual coordination task following transcranial alternating current stimulation (tACS): A combined EEG-fNIRS study. *Frontiers in Behavioral Neuroscience*, *12*, 67. https://doi.org/10.3389/fnbeh.2018.00067

Boldin, A. M., Geiger, R., & Emberson, L. L. (2018). The emergence of top-down, sensory prediction during learning in infancy: A comparison of full-term and preterm infants. *Developmental Psychobiology*, *60*(5), 544–556. https://doi.org/10.1002/dev.21624

Borragán, G., Gilson, M., Guerrero-Mosquera, C., Di Ricci, E., Slama, H., & Peigneux, P. (2018). Transcranial direct current stimulation does not counteract cognitive fatigue, but induces sleepiness and an inter-hemispheric shift in brain oxygenation. *Frontiers in Psychology*, *9*, 2351. https://doi.org/10.3389/fpsyg.2018.02351

Braukmann, R., Lloyd-Fox, S., Blasi, A., Johnson, M. H., Bekkering, H., Buitelaar, J. K., & Hunnius, S. (2018). Diminished socially selective neural processing in 5-month-old infants at high familial risk of autism. *European Journal of Neuroscience*, *47*(6), 720–728. https://doi.org/10.1111/ejn.13751

Brockington, G., Balardin, J. B., Zimeo Morais, G. A., Malheiros, A., Lent, R., Moura, L. M., & Sato, J. R. (2018). From the laboratory to the classroom: The potential of functional near-infrared spectroscopy in educational neuroscience. *Frontiers in Psychology*, *9*, 1840. https://doi.org/10.3389/fpsyg.2018.01840

Bruno, J. L., Baker, J. M., Gundran, A., Harbott, L. K., Stuart, Z., Piccirilli, A. M., Hosseini, S. M. H., Gerdes, J. C., & Reiss, A. L. (2018). Mind over motor mapping: Driver response

to changing vehicle dynamics. *Human Brain Mapping*, *39*(10), 3915–3927. https://doi.org/10.1002/hbm.24220

Bulgarelli, C., Blasi, A., Arridge, S., Powell, S., de Klerk, C. C. J. M., Southgate, V., Brigadoi, S., Penny, W., Tak, S., & Hamilton, A. (2018). Dynamic causal modelling on infant fNIRS data: A validation study on a simultaneously recorded fNIRS-fMRI dataset. *NeuroImage*, *175*, 413–424. https://doi.org/10.1016/j.neuroimage.2018.04.022

Burns, S. M., Barnes, L. N., Katzman, P. L., Ames, D. L., Falk, E. B., & Lieberman, M. D. (2018). A functional near infrared spectroscopy (fNIRS) replication of the sunscreen persuasion paradigm. *Social Cognitive and Affective Neuroscience*, *13*(6), 628–636. https://doi.org/10.1093/scan/nsy030

Caçola, P., Getchell, N., Srinivasan, D., Alexandrakis, G., & Liu, H. (2018). Cortical activity in fine-motor tasks in children with Developmental Coordination Disorder: A preliminary fNIRS study. *International Journal of Developmental Neuroscience*, *65*(1), 83–90. https://doi.org/10.1016/j.ijdevneu.2017.11.001

Çakir, M. P., Çakar, T., Girisken, Y., & Yurdakul, D. (2018). An investigation of the neural correlates of purchase behavior through fNIRS. *European Journal of Marketing*, *52*(1/2), 224–243. https://doi.org/10.1108/EJM-12-2016-0864

Carrieri, M., Lancia, S., Bocchi, A., Ferrari, M., Piccardi, L., & Quaresima, V. (2018). Does ventrolateral prefrontal cortex help in searching for the lost key? Evidence from an fNIRS study. *Brain Imaging and Behavior*, *12*(3), 785–797. https://doi.org/10.1007/s11682-017-9734-7

Chen, Y., Yu, Y., Niu, R., & Liu, Y. (2018). Selective effects of postural control on spatial vs. nonspatial working memory: A functional near-infrared spectral imaging study. *Frontiers in Human Neuroscience*, *12*, 243. https://doi.org/10.3389/fnhum.2018.00243

Chiarelli, A. M., Croce, P., Merla, A., & Zappasodi, F. (2018). Deep learning for hybrid EEG-fNIRS brain–computer interface: Application to motor imagery classification. *Journal of Neural Engineering*, *15*(3), 036028. https://doi.org/10.1088/1741-2552/aaaf82

Chuang, C.-H., Cao, Z., King, J.-T., Wu, B.-S., Wang, Y.-K., & Lin, C.-T. (2018). Brain electrodynamic and hemodynamic signatures against fatigue during driving. *Frontiers in Neuroscience*, *12*, 181. https://doi.org/10.3389/fnins.2018.00181

Cinciute, S., Daktariunas, A., & Ruksenas, O. (2018). Hemodynamic effects of sex and handedness on the Wisconsin Card Sorting Test: The contradiction between neuroimaging and behavioural results. *PeerJ*, *6*, e5890. https://doi.org/10.7717/peerj.5890

Corp, D. T., Youssef, G. J., Clark, R. A., Gomes-Osman, J., Yücel, M. A., Oldham, S. J., Aldraiwiesh, S., Rice, J., Pascual-Leone, A., & Rogers, M. A. (2018). Reduced motor cortex inhibition and a ‘cognitive-first’ prioritisation strategy for older adults during dual-tasking. *Experimental Gerontology*, *113*, 95–105. https://doi.org/10.1016/j.exger.2018.09.018

Crivelli, D., Sabogal Rueda, M. D., & Balconi, M. (2018). Linguistic and motor representations of everyday complex actions: An fNIRS investigation. *Brain Structure and Function*, *223*(6), 2989–2997. https://doi.org/10.1007/s00429-018-1646-9

Dai, R., Liu, R., Liu, T., Zhang, Z., Xiao, X., Sun, P., Yu, X., Wang, D., & Zhu, C. (2018). Holistic cognitive and neural processes: A fNIRS-hyperscanning study on interpersonal

sensorimotor synchronization. *Social Cognitive and Affective Neuroscience*, *13*(11), 1141–1154. https://doi.org/10.1093/scan/nsy090

Dashtestani, H., Zaragoza, R., Kermanian, R., Knutson, K. M., Halem, M., Casey, A., Shahni Karamzadeh, N., Anderson, A. A., Boccara, A. C., & Gandjbakhche, A. (2018). The role of prefrontal cortex in a moral judgment task using functional near-infrared spectroscopy. *Brain and Behavior*, *8*(11), e01116. https://doi.org/10.1002/brb3.1116

Dashtestani, H., Zaragoza, R., Pirsiavash, H., Knutson, K. M., Kermanian, R., Cui, J., Harrison, J. D., Halem, M., & Gandjbakhche, A. (2019). Canonical correlation analysis of brain prefrontal activity measured by functional near infra-red spectroscopy (fNIRS) during a moral judgment task. *Behavioural Brain Research*, *359*, 73–80. https://doi.org/10.1016/j.bbr.2018.10.022

de Klerk, C. C. J. M., Hamilton, A. F. de C., & Southgate, V. (2018). Eye contact modulates facial mimicry in 4-month-old infants: An EMG and fNIRS study. *Cortex*, *106*, 93–103. https://doi.org/10.1016/j.cortex.2018.05.002

de Oliveira, S. R., de Paula Machado, A. C. C., de Paula, J. J., de Moraes, P. H. P., Nahin, M. J. S., Magalhães, L. de C., Novi, S. L., Mesquita, R. C., de Miranda, D. M., & Bouzada, M. C. F. (2018). Association between hemodynamic activity and motor performance in six-month-old full-term and preterm infants: A functional near-infrared spectroscopy study. *Neurophotonics*, *5*(1), 011016. https://doi.org/10.1117/1.NPh.5.1.011016

Delgado Reyes, L. M., Bohache, K., Wijeakumar, S., & Spencer, J. P. (2018). Evaluating motion processing algorithms for use with functional near-infrared spectroscopy data from young children. *Neurophotonics*, *5*(2), 025008. https://doi.org/10.1117/1.NPh.5.2.025008

Dong, D., Wong, L. K. F., & Luo, Z. (2019). Assess BA10 activity in slide-based and immersive virtual reality prospective memory task using functional near-infrared spectroscopy (fNIRS). *Applied Neuropsychology: Adult*, *26*(5), 465–471. https://doi.org/10.1080/23279095.2018.1443104

Dong, S., & Jeong, J. (2018). Process-specific analysis in episodic memory retrieval using fast optical signals and hemodynamic signals in the right prefrontal cortex. *Journal of Neural Engineering*, *15*(1), 015001. https://doi.org/10.1088/1741-2552/aa91b5

Dong, S., & Jeong, J. (2019). Improvement in recovery of hemodynamic responses by extended kalman filter with non-linear state-space model and short separation measurement. *IEEE Transactions on Biomedical Engineering*, *66*(8), 2152–2162. https://doi.org/10.1109/TBME.2018.2884169

Dravida, S., Noah, J. A., Zhang, X., & Hirsch, J. (2018). Comparison of oxyhemoglobin and deoxyhemoglobin signal reliability with and without global mean removal for digit manipulation motor tasks. *Neurophotonics*, *5*(1), 011006. https://doi.org/10.1117/1.NPh.5.1.011006

Duan, L., Zhao, Z., Lin, Y., Wu, X., Luo, Y., & Xu, P. (2018). Wavelet-based method for removing global physiological noise in functional near-infrared spectroscopy. *Biomedical Optics Express*, *9*(8), 3805. https://doi.org/10.1364/BOE.9.003805

Eken, A., Gökçay, D., Yılmaz, C., Baskak, B., Baltacı, A., & Kara, M. (2018). Association of fine motor loss and allodynia in fibromyalgia: An fNIRS study. *Journal of Motor Behavior*, *50*(6), 664–676. https://doi.org/10.1080/00222895.2017.1400947

Emberson, L. L., Boldin, A. M., Robertson, C. E., Cannon, G., & Aslin, R. N. (2019). Expectation affects neural repetition suppression in infancy. *Developmental Cognitive Neuroscience*, *37*, 100597. https://doi.org/10.1016/j.dcn.2018.11.001

Eskicioglu, E., Taslica, S., Narin, B., Guducu, C., Oniz, A., & Ozgoren, M. (2019). Brain asymmetry in directing attention during dichotic listening test: An fNIRS study. *Laterality: Asymmetries of Body, Brain and Cognition*, *24*(4), 377–392. https://doi.org/10.1080/1357650X.2018.1527847

Fairclough, S. H., Burns, C., & Kreplin, U. (2018). FNIRS activity in the prefrontal cortex and motivational intensity: Impact of working memory load, financial reward, and correlation-based signal improvement. *Neurophotonics*, *5*(03), 035001. https://doi.org/10.1117/1.NPh.5.3.035001

Fishburn, F. A., Hlutkowsky, C. O., Bemis, L. M., Huppert, T. J., Wakschlag, L. S., & Perlman, S. B. (2019). Irritability uniquely predicts prefrontal cortex activation during preschool inhibitory control among all temperament domains: A LASSO approach. *NeuroImage*, *184*, 68–77. https://doi.org/10.1016/j.neuroimage.2018.09.023

Fishburn, F. A., Murty, V. P., Hlutkowsky, C. O., MacGillivray, C. E., Bemis, L. M., Murphy, M. E., Huppert, T. J., & Perlman, S. B. (2018). Putting our heads together: Interpersonal neural synchronization as a biological mechanism for shared intentionality. *Social Cognitive and Affective Neuroscience*, *13*(8), 841–849. https://doi.org/10.1093/scan/nsy060

Foy, H. J., & Chapman, P. (2018). Mental workload is reflected in driver behaviour, physiology, eye movements and prefrontal cortex activation. *Applied Ergonomics*, *73*, 90–99. https://doi.org/10.1016/j.apergo.2018.06.006

Frie, J., Bartocci, M., Lagercrantz, H., & Kuhn, P. (2018). Cortical responses to alien odors in newborns: An fNIRS study. *Cerebral Cortex*, *28*(9), 3229–3240. https://doi.org/10.1093/cercor/bhx194

Gagrani, M., Faiq, M. A., Sidhu, T., Dada, R., Yadav, R. K., Sihota, R., Kochhar, K. P., Verma, R., & Dada, T. (2018). Meditation enhances brain oxygenation, upregulates BDNF and improves quality of life in patients with primary open angle glaucoma: A randomized controlled trial. *Restorative Neurology and Neuroscience*, *36*(6), 741–753. https://doi.org/10.3233/RNN-180857

Gateau, T., Ayaz, H., & Dehais, F. (2018). In silico vs. over the clouds: On-the-Fly mental state estimation of aircraft pilots, using a functional near infrared spectroscopy based passive-BCI. *Frontiers in Human Neuroscience*, *12*, 187. https://doi.org/10.3389/fnhum.2018.00187

Gemignani, J., Bayet, L., Kabdebon, C., Blankertz, B., Pugh, K. R., & Aslin, R. N. (2018). Classifying the mental representation of word meaning in children with Multivariate Pattern Analysis of fNIRS. *IEEE*, 295–298. https://doi.org/10.1109/EMBC.2018.8512209

George, C. J., Verghese, J., Izzetoglu, M., Wang, C., & Holtzer, R. (2019). The effect of polypharmacy on prefrontal cortex activation during single and dual task walking in community dwelling older adults. *Pharmacological Research*, *139*, 113–119. https://doi.org/10.1016/j.phrs.2018.11.007

Giles, G. E., Cantelon, J. A., Eddy, M. D., Brunyé, T. T., Urry, H. L., Taylor, H. A., Mahoney, C. R., & Kanarek, R. B. (2018). Cognitive reappraisal reduces perceived exertion during endurance exercise. *Motivation and Emotion*, *42*(4), 482–496. https://doi.org/10.1007/s11031-018-9697-z

Giles, G. E., Eddy, M. D., Brunyé, T. T., Urry, H. L., Graber, H. L., Barbour, R. L., Mahoney, C. R., Taylor, H. A., & Kanarek, R. B. (2018). Endurance exercise enhances emotional valence and emotion regulation. *Frontiers in Human Neuroscience*, *12*, 398. https://doi.org/10.3389/fnhum.2018.00398

Grabell, A. S., Li, Y., Barker, J. W., Wakschlag, L. S., Huppert, T. J., & Perlman, S. B. (2018). Evidence of non-linear associations between frustration-related prefrontal cortex activation and the normal:abnormal spectrum of irritability in young children. *Journal of Abnormal Child Psychology*, *46*(1), 137–147. https://doi.org/10.1007/s10802-017-0286-5

Groba, A., De Houwer, A., Mehnert, J., Rossi, S., & Obrig, H. (2018). Bilingual and monolingual children process pragmatic cues differently when learning novel adjectives. *Bilingualism: Language and Cognition*, *21*(2), 384–402. https://doi.org/10.1017/S1366728917000232

Groff, B. R., Antonellis, P., Schmid, K. K., Knarr, B. A., & Stergiou, N. (2019). Stride-time variability is related to sensorimotor cortical activation during forward and backward walking. *Neuroscience Letters*, *692*, 150–158. https://doi.org/10.1016/j.neulet.2018.10.022

Grossmann, T., Missana, M., & Krol, K. M. (2018). The neurodevelopmental precursors of altruistic behavior in infancy. *PLOS Biology*, *16*(9), e2005281. https://doi.org/10.1371/journal.pbio.2005281

Gu, Y., Miao, S., Han, J., Liang, Z., Ouyang, G., Yang, J., & Li, X. (2018). Identifying ADHD children using hemodynamic responses during a working memory task measured by functional near-infrared spectroscopy. *Journal of Neural Engineering*, *15*(3), 035005. https://doi.org/10.1088/1741-2552/aa9ee9

Hakimi, N., & Kamaledin Setarehdan, S. (2018). Stress assessment by means of heart rate derived from functional near-infrared spectroscopy. *Journal of Biomedical Optics*, *23*(11), 115001. https://doi.org/10.1117/1.JBO.23.11.115001

Hakuno, Y., Pirazzoli, L., Blasi, A., Johnson, M. H., & Lloyd-Fox, S. (2018). Optical imaging during toddlerhood: Brain responses during naturalistic social interactions. *Neurophotonics*, *5*(1), 011020. https://doi.org/10.1117/1.NPh.5.1.011020

Halliday, D. W. R., Hundza, S. R., Garcia-Barrera, M. A., Klimstra, M., Commandeur, D., Lukyn, T. V., Stawski, R. S., & MacDonald, S. W. S. (2018). Comparing executive function, evoked hemodynamic response, and gait as predictors of variations in mobility for older adults. *Journal of Clinical and Experimental Neuropsychology*, *40*(2), 151–160. https://doi.org/10.1080/13803395.2017.1325453

Halliday, D. W. R., Mulligan, B. P., Garrett, D. D., Schmidt, S., Hundza, S. R., Garcia-Barrera, M. A., Stawski, R. S., & MacDonald, S. W. S. (2018). Mean and variability in functional brain activations differentially predict executive function in older adults: An investigation employing functional near-infrared spectroscopy. *Neurophotonics*, *5*(1), 011013. https://doi.org/10.1117/1.NPh.5.1.011013

Han, C.-H., Hwang, H.-J., Lim, J.-H., & Im, C.-H. (2018). Assessment of user voluntary engagement during neurorehabilitation using functional near-infrared spectroscopy: A preliminary study. *Journal of NeuroEngineering and Rehabilitation*, *15*(1), 1–10. https://doi.org/10.1186/s12984-018-0365-z

Harris, D. M., Rantalainen, T., Muthalib, M., Johnson, L., Duckham, R. L., Smith, S. T., Daly, R. M., & Teo, W.-P. (2018). Concurrent exergaming and transcranial direct current stimulation to improve balance in people with Parkinson’s disease: Study protocol for a

randomised controlled trial. *Trials*, *19*(1), 387. https://doi.org/10.1186/s13063-018-2773-6

Harrison, S. J., Hough, M., Schmid, K., Groff, B. R., & Stergiou, N. (2018). When coordinating finger tapping to a variable beat the variability scaling structure of the movement and the cortical BOLD signal are both entrained to the auditory stimuli. *Neuroscience*, *392*, 203–218. https://doi.org/10.1016/j.neuroscience.2018.06.025

Hawkins, K. A., Fox, E. J., Daly, J. J., Rose, D. K., Christou, E. A., McGuirk, T. E., Otzel, D. M., Butera, K. A., Chatterjee, S. A., & Clark, D. J. (2018). Prefrontal over-activation during walking in people with mobility deficits: Interpretation and functional implications. *Human Movement Science*, *59*, 46–55. https://doi.org/10.1016/j.humov.2018.03.010

Hirata, K., Egashira, K., Harada, K., Nakashima, M., Hirotsu, M., Isomura, S., Watanuki, T., Matsubara, T., Kaku, Y., Kaneyuki, H., Watanabe, Y., & Matsuo, K. (2018). Differences in frontotemporal dysfunction during social and non-social cognition tasks between patients with autism spectrum disorder and schizophrenia. *Scientific Reports*, *8*(1), 1–10. https://doi.org/10.1038/s41598-018-21379-w

Hirsch, J., Adam Noah, J., Zhang, X., Dravida, S., & Ono, Y. (2018). A cross-brain neural mechanism for human-to-human verbal communication. *Social Cognitive and Affective Neuroscience*, *13*(9), 907–920. https://doi.org/10.1093/scan/nsy070

Hiwa, S., Katayama, T., & Hiroyasu, T. (2018). Functional near-infrared spectroscopy study of the neural correlates between auditory environments and intellectual work performance. *Brain and Behavior*, *8*(10), e01104. https://doi.org/10.1002/brb3.1104

Hocke, L. M., Duszynski, C. C., Debert, C. T., Dleikan, D., & Dunn, J. F. (2018). Reduced functional connectivity in adults with persistent post-concussion symptoms: A functional near-infrared spectroscopy study. *Journal of Neurotrauma*, *35*(11), 1224–1232. https://doi.org/10.1089/neu.2017.5365

Hocke, L., Oni, I., Duszynski, C., Corrigan, A., Frederick, B., & Dunn, J. (2018). Automated processing of fNIRS data—A visual guide to the pitfalls and consequences. *Algorithms*, *11*(5), 67. https://doi.org/10.3390/a11050067

Holper, L., & Mann, J. J. (2018). Test–retest reliability of brain mitochondrial cytochrome-c-oxidase assessed by functional near-infrared spectroscopy. *Journal of Biomedical Optics*, *23*(05), 1. https://doi.org/10.1117/1.JBO.23.5.056006

Holtzer, R., George, C. J., Izzetoglu, M., & Wang, C. (2018). The effect of diabetes on prefrontal cortex activation patterns during active walking in older adults. *Brain and Cognition*, *125*, 14–22. https://doi.org/10.1016/j.bandc.2018.03.002

Hoppes, C. W., Sparto, P. J., Whitney, S. L., Furman, J. M., & Huppert, T. J. (2018). Functional near-infrared spectroscopy during optic flow with and without fixation. *PLOS ONE*, *13*(3), e0193710. https://doi.org/10.1371/journal.pone.0193710

Hosgoren Alici, Y., Devrimci Ozguven, H., Kale, E., Yenihayat, I., & Baskak, B. (2018). Prefrontal activity measured by functional near infrared spectroscopy during divergent and convergent thinking in bipolar disorder". *Archives of Neuropsychiatry*, *56*(2), 86. https://doi.org/10.29399/npa.23203

Hosseini, R., Walsh, B., Tian, F., & Wang, S. (2018). An fNIRS-based feature learning and classification framework to distinguish hemodynamic patterns in children who stutter.

IEEE Transactions on Neural Systems and Rehabilitation Engineering, *26*(6), 1254–1263. https://doi.org/10.1109/TNSRE.2018.2829083

Hu, Z., Zhang, J., Couto, T. A., Xu, S., Luan, P., & Yuan, Z. (2018). Optical mapping of brain activation and connectivity in occipitotemporal cortex during chinese character recognition. *Brain Topography*, *31*(6), 1014–1028. https://doi.org/10.1007/s10548-018-0650-y

Huhn, A. S., Sweeney, M. M., Brooner, R. K., Kidorf, M. S., Tompkins, D. A., Ayaz, H., & Dunn, K. E. (2019). Prefrontal cortex response to drug cues, craving, and current depressive symptoms are associated with treatment outcomes in methadone-maintained patients. *Neuropsychopharmacology*, *44*(4), 826–833. https://doi.org/10.1038/s41386-018-0252-0

Ihme, K., Unni, A., Zhang, M., Rieger, J. W., & Jipp, M. (2018). Recognizing frustration of drivers from face video recordings and brain activation measurements with functional near-infrared spectroscopy. *Frontiers in Human Neuroscience*, *12*, 327. https://doi.org/10.3389/fnhum.2018.00327

Ikeda, T., Tokuda, T., Monden, Y., Hirai, M., Mizushima, S. G., Nagashima, M., Kyutoku, Y., Taniguchi, T., Shimoizumi, H., Dan, I., & Yamagata, T. (2018). Hypoactivation of the right prefrontal cortex underlying motor-related inhibitory deficits in children with Autism Spectrum Disorder: A functional near-infrared spectroscopy study: Hypoactivation of PFC in ASD. *Japanese Psychological Research*, *60*(4), 251–264. https://doi.org/10.1111/jpr.12204

Jasińska, K. K., & Guei, S. (2018). Neuroimaging field methods using functional near infrared spectroscopy (NIRS) neuroimaging to study global child development: Rural sub-saharan africa. *Journal of Visualized Experiments*, *132*, 57165. https://doi.org/10.3791/57165

Jeong, E., Ryu, H., Jo, G., & Kim, J. (2018). Cognitive load changes during music listening and its implication in earcon design in public environments: An fNIRS study. *International Journal of Environmental Research and Public Health*, *15*(10), 2075. https://doi.org/10.3390/ijerph15102075

Jeong, E., Ryu, H., Shin, J.-H., Kwon, G. H., Jo, G., & Lee, J.-Y. (2018). High oxygen exchange to music indicates auditory distractibility in acquired brain injury: An fNIRS study with a vector-based phase analysis. *Scientific Reports*, *8*(1), 16737. https://doi.org/10.1038/s41598-018-35172-2

Jia, H., Li, Y., & Yu, D. (2018). Normalized spatial complexity analysis of neural signals. *Scientific Reports*, *8*(1), 1–10. https://doi.org/10.1038/s41598-018-26329-0

Jin, H., Li, C., & Xu, J. (2018). Pilot study on gait classification using fNIRS signals. *Computational Intelligence and Neuroscience*, *2018*, 1–9. https://doi.org/10.1155/2018/7403471

Kalia, V., Vishwanath, K., Knauft, K., Vellen, B. V. D., Luebbe, A., & Williams, A. (2018). Acute stress attenuates cognitive flexibility in males only: An fNIRS examination. *Frontiers in Psychology*, *9*, 2084. https://doi.org/10.3389/fpsyg.2018.02084

Kamarunas, E., Mulheren, R., Palmore, K., & Ludlow, C. (2018). Timing of cortical activation during spontaneous swallowing. *Experimental Brain Research*, *236*(2), 475–484. https://doi.org/10.1007/s00221-017-5139-5

Kamran, M. A., Naeem Mannan, M. M., & Jeong, M.-Y. (2018). Initial-dip existence and estimation in relation to DPF and data drift. *Frontiers in Neuroinformatics*, *12*, 96. https://doi.org/10.3389/fninf.2018.00096

Kassab, A., Le Lan, J., Tremblay, J., Vannasing, P., Dehbozorgi, M., Pouliot, P., Gallagher, A., Lesage, F., Sawan, M., & Nguyen, D. K. (2018). Multichannel wearable fNIRS-EEG system for long-term clinical monitoring: Multichannel wearable fNIRS-EEG system. *Human Brain Mapping*, *39*(1), 7–23. https://doi.org/10.1002/hbm.23849

Katzorke, A., Zeller, J. B. M., Müller, L. D., Lauer, M., Polak, T., Deckert, J., & Herrmann, M. J. (2018). Decreased hemodynamic response in inferior frontotemporal regions in elderly with mild cognitive impairment. *Psychiatry Research: Neuroimaging*, *274*, 11–18. https://doi.org/10.1016/j.pscychresns.2018.02.003

Khan, M. J., Ghafoor, U., & Hong, K.-S. (2018). Early detection of hemodynamic responses using EEG: A hybrid EEG-fNIRS study. *Frontiers in Human Neuroscience*, *12*, 479. https://doi.org/10.3389/fnhum.2018.00479

Khan, R. A., Naseer, N., Qureshi, N. K., Noori, F. M., Nazeer, H., & Khan, M. U. (2018). FNIRS-based neurorobotic interface for gait rehabilitation. *Journal of NeuroEngineering and Rehabilitation*, *15*(1), 1–17. https://doi.org/10.1186/s12984-018-0346-2

Krampe, C., Gier, N. R., & Kenning, P. (2018). The application of mobile fNIRS in marketing research—Detecting the "first-choice-brand" effect. *Frontiers in Human Neuroscience, 12*, 433. https://doi.org/10.3389/fnhum.2018.00433

Krampe, C., Strelow, E., Haas, A., & Kenning, P. (2018). The application of mobile fNIRS to "shopper neuroscience" – first insights from a merchandising communication study.

European Journal of Marketing, *52*(1/2), 244–259. https://doi.org/10.1108/EJM-12-2016-0727

Kujach, S., Byun, K., Hyodo, K., Suwabe, K., Fukuie, T., Laskowski, R., Dan, I., & Soya, H. (2018). A transferable high-intensity intermittent exercise improves executive performance in association with dorsolateral prefrontal activation in young adults. *NeuroImage*, *169*, 117–125. https://doi.org/10.1016/j.neuroimage.2017.12.003

Kurz, E.-M., Wood, G., Kober, S. E., Schippinger, W., Pichler, G., Müller-Putz, G., & Bauernfeind, G. (2018). Towards using fNIRS recordings of mental arithmetic for the detection of residual cognitive activity in patients with disorders of consciousness (DOC). *Brain and Cognition*, *125*, 78–87. https://doi.org/10.1016/j.bandc.2018.06.002

Lancia, S., Cofini, V., Carrieri, M., Ferrari, M., & Quaresima, V. (2018). Are ventrolateral and dorsolateral prefrontal cortices involved in the computerized Corsi block-tapping test execution? An fNIRS study. *Neurophotonics*, *5*(1), 011019. https://doi.org/10.1117/1.NPh.5.1.011019

Landowska, A., Roberts, D., Eachus, P., & Barrett, A. (2018). Within- and between-session prefrontal cortex response to virtual reality exposure therapy for acrophobia. *Frontiers in Human Neuroscience*, *12*, 362. https://doi.org/10.3389/fnhum.2018.00362

Langhanns, C., & Müller, H. (2018). Empirical support for 'hastening-through-re-automatization' by contrasting two motor-cognitive dual tasks. *Frontiers in Psychology*, *9*, 714. https://doi.org/10.3389/fpsyg.2018.00714

Lawrence, R. J., Wiggins, I. M., Anderson, C. A., Davies-Thompson, J., & Hartley, D. E. H. (2018). Cortical correlates of speech intelligibility measured using functional near-

infrared spectroscopy (fNIRS). *Hearing Research*, *370*, 53–64. https://doi.org/10.1016/j.heares.2018.09.005

Lee, S. H., Jin, S. H., & An, J. (2018). Distinction of directional coupling in sensorimotor networks between active and passive finger movements using fNIRS. *Biomedical Optics Express*, *9*(6), 2859–2870. https://doi.org/10.1364/BOE.9.002859

Li, F., Zhu, H., Xu, J., Gao, Q., Guo, H., Wu, S., Li, X., & He, S. (2018). Lie detection using fNIRS monitoring of inhibition-related brain regions discriminates infrequent but not frequent liars. *Frontiers in Human Neuroscience*, *12*, 71. https://doi.org/10.3389/fnhum.2018.00071

Li, H., Subrahmanyam, K., Bai, X., Xie, X., & Liu, T. (2018). Viewing fantastical events versus touching fantastical events: Short-term effects on children's inhibitory control. *Child Development*, *89*(1), 48–57. https://doi.org/10.1111/cdev.12820

Li, Q., Becker, B., Wernicke, J., Chen, Y., Zhang, Y., Li, R., Le, J., Kou, J., Zhao, W., & Kendrick, K. M. (2019). Foot massage evokes oxytocin release and activation of orbitofrontal cortex and superior temporal sulcus. *Psychoneuroendocrinology*, *101*, 193–203. https://doi.org/10.1016/j.psyneuen.2018.11.016

Li, R., Rui, G., Chen, W., Li, S., Schulz, P. E., & Zhang, Y. (2018). Early detection of alzheimer's disease using non-invasive near-infrared spectroscopy. *Frontiers in Aging Neuroscience*, *10*, 366. https://doi.org/10.3389/fnagi.2018.00366

Li, T., Lin, Y., Gao, Y., & Zhong, F. (2018). Longtime driving induced cerebral hemodynamic elevation and behavior degradation as assessed by functional near-infrared spectroscopy and a voluntary attention test. *Journal of Biophotonics*, *11*(12), e201800160. https://doi.org/10.1002/jbio.201800160

Li, Y., Chen, R., Zhang, S., Turel, O., Bechara, A., Feng, T., Chen, H., & He, Q. (2019). Hemispheric mPFC asymmetry in decision making under ambiguity and risk: An fNIRS study. *Behavioural Brain Research*, *359*, 657–663. https://doi.org/10.1016/j.bbr.2018.09.021

Li, Y., Zhang, L., Long, K., Gong, H., & Lei, H. (2018). Real-time monitoring prefrontal activities during online video game playing by functional near-infrared spectroscopy. *Journal of Biophotonics*, *11*(9), e201700308. https://doi.org/10.1002/jbio.201700308

Lin, X., Lei, V. L. C., & Li, D. (2018). Which is more costly in Chinese to English simultaneous interpreting, “pairing” or “transphrasing”? Evidence from an fNIRS neuroimaging study. *Neurophotonics*, *5*(2), 025010. https://doi.org/10.1117/1.NPh.5.2.025010

Lin, X., Sai, L., & Yuan, Z. (2018). Detecting concealed information with fused electroencephalography and functional near-infrared spectroscopy. *Neuroscience*, *386*, 284–294. https://doi.org/10.1016/j.neuroscience.2018.06.049

Liu, X., Kim, C.-S., & Hong, K.-S. (2018). An fNIRS-based investigation of visual merchandising displays for fashion stores. *PLOS ONE*, *13*(12), e0208843. https://doi.org/10.1371/journal.pone.0208843

Liu, Y., & Ayaz, H. (2018). Speech recognition via fNIRS based brain signals. *Frontiers in Neuroscience*, *12*, 695. https://doi.org/10.3389/fnins.2018.00695

Lloyd-Fox, S., Blasi, A., Pasco, G., Gliga, T., Jones, E. J. H., Murphy, D. G. M., Elwell, C. E., Charman, T., Johnson, M. H., & the BASIS Team. (2018). Cortical responses before 6 months of life associate with later autism. *European Journal of Neuroscience*, *47*(6), 736–749. https://doi.org/10.1111/ejn.13757

Mahmoudzadeh, M., Dehaene-Lambertz, G., Kongolo, G., Fournier, M., Goudjil, S., & Wallois, F. (2018). Consequence of intraventricular hemorrhage on neurovascular coupling evoked by speech syllables in preterm neonates. *Developmental Cognitive Neuroscience*, *30*, 60–69. https://doi.org/10.1016/j.dcn.2018.01.001

Maidan, I., Nieuwhof, F., Bernad-Elazari, H., Bloem, B. R., Giladi, N., Hausdorff, J. M., Claassen, J. A. H. R., & Mirelman, A. (2018). Evidence for differential effects of 2 Forms of exercise on prefrontal plasticity during walking in parkinson's disease. *Neurorehabilitation and Neural Repair*, *32*(3), 200–208. https://doi.org/10.1177/1545968318763750

Maidan, I., Shustak, S., Sharon, T., Bernad-Elazari, H., Geffen, N., Giladi, N., Hausdorff, J. M., & Mirelman, A. (2018). Prefrontal cortex activation during obstacle negotiation: What's the effect size and timing? *Brain and Cognition*, *122*, 45–51. https://doi.org/10.1016/j.bandc.2018.02.006

Maier, M. J., Rosenbaum, D., Haeussinger, F. B., Brüne, M., Enzi, B., Plewnia, C., Fallgatter, A. J., & Ehlis, A.-C. (2018). Forgiveness and cognitive control – Provoking revenge via theta-burst-stimulation of the DLPFC. *NeuroImage*, *183*, 769–775. https://doi.org/10.1016/j.neuroimage.2018.08.065

Maior, H. A., Wilson, M. L., & Sharples, S. (2018). Workload Alerts—Using physiological measures of mental workload to provide feedback during tasks. *ACM Transactions on Computer-Human Interaction*, *25*(2), 1–30. https://doi.org/10.1145/3173380

Meyerding, S. G. H., & Risius, A. (2018). Reading minds: Mobile functional near-infrared spectroscopy as a new neuroimaging method for economic and marketing research—A

feasibility study. *Journal of Neuroscience, Psychology, and Economics*, *11*(4), 197–212. https://doi.org/10.1037/npe0000090

Miguel, H. O., Gonçalves, Ó. F., Cruz, S., & Sampaio, A. (2019). Infant brain response to affective and discriminative touch: A longitudinal study using fNIRS. *Social Neuroscience*, *14*(5), 571–582. https://doi.org/10.1080/17470919.2018.1536000

Miller, J. G., Vrtička, P., Cui, X., Shrestha, S., Hosseini, S. M. H., Baker, J. M., & Reiss, A. L. (2019). Inter-brain synchrony in mother-child dyads during cooperation: An fNIRS hyperscanning study. *Neuropsychologia*, *124*, 117–124. https://doi.org/10.1016/j.neuropsychologia.2018.12.021

Minematsu, Y., Ueji, K., & Yamamoto, T. (2018). Activity of frontal pole cortex reflecting hedonic tone of food and drink: FNIRS study in humans. *Scientific Reports*, *8*(1), 16197. https://doi.org/10.1038/s41598-018-34690-3

Morita, I., Sakuma, S., Shimomura, J., Hayashi, N., & Toda, S. (2018). Brain activity in response to the touch of a hand on the center of the back. *PLOS ONE*, *13*(10), e0206451. https://doi.org/10.1371/journal.pone.0206451

Muthalib, M., Ferrari, M., Quaresima, V., Kerr, G., & Perrey, S. (2018). Functional near-infrared spectroscopy to probe sensorimotor region activation during electrical stimulation-evoked movement. *Clinical Physiology and Functional Imaging*, *38*(5), 816–822. https://doi.org/10.1111/cpf.12485

Narita, N., Kamiya, K., Makiyama, Y., Iwaki, S., Komiyama, O., Ishii, T., & Wake, H. (2019). Prefrontal modulation during chewing performance in occlusal dysesthesia patients: A functional near-infrared spectroscopy study. *Clinical Oral Investigations*, *23*(3), 1181–1196. https://doi.org/10.1007/s00784-018-2534-7

Nemani, A., Kruger, U., Cooper, C. A., Schwaitzberg, S. D., Intes, X., & De, S. (2019). Objective assessment of surgical skill transfer using non-invasive brain imaging. *Surgical Endoscopy*, *33*(8), 2485–2494. https://doi.org/10.1007/s00464-018-6535-z

Nguyen, T., Babawale, O., Kim, T., Jo, H. J., Liu, H., & Kim, J. G. (2018). Exploring brain functional connectivity in rest and sleep states: A fNIRS study. *Scientific Reports*, *8*(1), 16144. https://doi.org/10.1038/s41598-018-33439-2

Niioka, K., Uga, M., Nagata, T., Tokuda, T., Dan, I., & Ochi, K. (2018). Cerebral hemodynamic response during concealment of information about a mock crime: Application of a general linear model with an adaptive hemodynamic response function: Cerebral hemodynamic response during concealment of information. *Japanese Psychological Research*, *60*(4), 311–326. https://doi.org/10.1111/jpr.12194

Ochi, G., Yamada, Y., Hyodo, K., Suwabe, K., Fukuie, T., Byun, K., Dan, I., & Soya, H. (2018). Neural basis for reduced executive performance with hypoxic exercise. *NeuroImage*, *171*, 75–83. https://doi.org/10.1016/j.neuroimage.2017.12.091

Oh, S., Song, M., & Kim, J. (2018). Validating attentive locomotion training using interactive treadmill: An fNIRS study. *Journal of NeuroEngineering and Rehabilitation*, *15*(1), 122. https://doi.org/10.1186/s12984-018-0472-x

Onuma, T., Maruyama, H., & Sakai, N. (2018). Enhancement of saltiness perception by monosodium glutamate taste and soy sauce odor: A near-infrared spectroscopy study. *Chemical Senses*, *43*(3), 151–167. https://doi.org/10.1093/chemse/bjx084

Pan, Y., Novembre, G., Song, B., Li, X., & Hu, Y. (2018). Interpersonal synchronization of inferior frontal cortices tracks social interactive learning of a song. *NeuroImage*, *183*, 280–290. https://doi.org/10.1016/j.neuroimage.2018.08.005

Peng, K., Yücel, M. A., Aasted, C. M., Steele, S. C., Boas, D. A., Borsook, D., & Becerra, L. (2018). Using prerecorded hemodynamic response functions in detecting prefrontal pain response: A functional near-infrared spectroscopy study. *Neurophotonics, 5*(1), 011018. https://doi.org/10.1117/1.NPh.5.1.011018

Peng, K., Yücel, M. A., Steele, S. C., Bittner, E. A., Aasted, C. M., Hoeft, M. A., Lee, A., George, E. E., Boas, D. A., Becerra, L., & Borsook, D. (2018). Morphine attenuates fNIRS signal associated with painful stimuli in the medial frontopolar cortex (medial BA 10). *Frontiers in Human Neuroscience*, *12*, 394. https://doi.org/10.3389/fnhum.2018.00394

Perpetuini, D., Bucco, R., Zito, M., & Merla, A. (2018). Study of memory deficit in Alzheimer's disease by means of complexity analysis of fNIRS signal. *Neurophotonics*, *5*(1), 011010. https://doi.org/10.1117/1.NPh.5.1.011010

Pfeifer, M., Scholkmann, F., & Labruyère, R. (2018). Signal processing in functional near-infrared spectroscopy (fNIRS): Methodological differences lead to different statistical results. *Frontiers in Human Neuroscience*, *11*(641). https://doi.org/10.3389/fnhum.2017.00641

Powell, L. J., Deen, B., & Saxe, R. (2018). Using individual functional channels of interest to study cortical development with fNIRS. *Developmental Science*, *21*(4), e12595. https://doi.org/10.1111/desc.12595

Reindl, V., Gerloff, C., Scharke, W., & Konrad, K. (2018). Brain-to-brain synchrony in parent-child dyads and the relationship with emotion regulation revealed by fNIRS-based hyperscanning. *NeuroImage*, *178*, 493–502. https://doi.org/10.1016/j.neuroimage.2018.05.060

Ren, H., Wang, M.-Y., He, Y., Du, Z., Zhang, J., Zhang, J., Li, D., & Yuan, Z. (2019). A novel phase analysis method for examining fNIRS neuroimaging data associated with Chinese/English sight translation. *Behavioural Brain Research*, *361*, 151–158. https://doi.org/10.1016/j.bbr.2018.12.032

Rhee, J., & Mehta, R. K. (2018). Functional connectivity during handgrip motor fatigue in older adults is obesity and sex-specific. *Frontiers in Human Neuroscience*, *12*, 455. https://doi.org/10.3389/fnhum.2018.00455

Richter, H. O., Forsman, M., Elcadi, G. H., Brautaset, R., Marsh, J. E., & Zetterberg, C. (2018). Prefrontal cortex oxygenation evoked by convergence load under conflicting stimulus-to-accommodation and stimulus-to-vergence eye-movements measured by NIRS. *Frontiers in Human Neuroscience*, *12*, 298. https://doi.org/10.3389/fnhum.2018.00298

Rojiani, R., Zhang, X., Noah, A., & Hirsch, J. (2018). Communication of emotion via drumming: Dual-brain imaging with functional near-infrared spectroscopy. *Social Cognitive and Affective Neuroscience*, *13*(10), 1047–1057. https://doi.org/10.1093/scan/nsy076

Rosenbaum, D., Blum, L., Schweizer, P., Fallgatter, A. J., Herrmann, M. J., Ehlis, A.-C., & Metzger, F. G. (2018). Comparison of speed versus complexity effects on the hemodynamic response of the trail making test in block designs. *Neurophotonics*, *5*(4), 045007. https://doi.org/10.1117/1.NPh.5.4.045007

Rosenbaum, D., Hilsendegen, P., Thomas, M., Haeussinger, F. B., Metzger, F. G., Nuerk, H.-C., Fallgatter, A. J., Nieratschker, V., & Ehlis, A.-C. (2018). Cortical hemodynamic changes during the Trier Social Stress Test: An fNIRS study. *NeuroImage*, *171*, 107–115. https://doi.org/10.1016/j.neuroimage.2017.12.061

Rosenbaum, D., Maier, M. J., Hudak, J., Metzger, F. G., Wells, A., Fallgatter, A. J., & Ehlis, A.-C. (2018). Neurophysiological correlates of the attention training technique: A component study. *NeuroImage: Clinical*, *19*, 1018–1024. https://doi.org/10.1016/j.nicl.2018.06.021

Rosenbaum, D., Thomas, M., Hilsendegen, P., Metzger, F. G., Haeussinger, F. B., Nuerk, H.-C., Fallgatter, A. J., Nieratschker, V., & Ehlis, A.-C. (2018). Stress-related dysfunction of the right inferior frontal cortex in high ruminators: An fNIRS study. *NeuroImage: Clinical*, *18*, 510–517. https://doi.org/10.1016/j.nicl.2018.02.022

Rowland, S. C., Hartley, D. E. H., & Wiggins, I. M. (2018). Listening in Naturalistic Scenes: What Can Functional Near-Infrared Spectroscopy and Intersubject Correlation Analysis Tell Us About the Underlying Brain Activity? *Trends in Hearing*, *22*, 233121651880411. https://doi.org/10.1177/2331216518804116

Saita, K., Morishita, T., Arima, H., Hyakutake, K., Ogata, T., Yagi, K., Shiota, E., & Inoue, T. (2018). Biofeedback effect of hybrid assistive limb in stroke rehabilitation: A proof of concept study using functional near infrared spectroscopy. *PLOS ONE*, *13*(1), e0191361. https://doi.org/10.1371/journal.pone.0191361

Sakurada, T., Hirai, M., & Watanabe, E. (2019). Individual optimal attentional strategy during implicit motor learning boosts frontoparietal neural processing efficiency: A functional near-infrared spectroscopy study. *Brain and Behavior*, *9*(1), e01183. https://doi.org/10.1002/brb3.1183

Saleh, S., Sandroff, B. M., Owoeye, O., Vitiello, T., Hoxha, A., Yue, G., & DeLuca, J. (2018). Supra-spinal modulation of walking in healthy individuals and persons with multiple

sclerosis: A fNIRS mobile imaging study. *IEEE*, 3156–3159. https://doi.org/10.1109/EMBC.2018.8513052

Saleh, S., Sandroff, B. M., Vitiello, T., Owoeye, O., Hoxha, A., Hake, P., Goverover, Y., Wylie, G., Yue, G., & DeLuca, J. (2018). The role of premotor areas in dual tasking in healthy controls and persons with multiple sclerosis: An fNIRS imaging study. *Frontiers in Behavioral Neuroscience*, *12*, 296. https://doi.org/10.3389/fnbeh.2018.00296

Scheunemann, J., Unni, A., Ihme, K., Jipp, M., & Rieger, J. W. (2019). Demonstrating brain-level interactions between visuospatial attentional demands and working memory load while driving using functional near-infrared spectroscopy. *Frontiers in Human Neuroscience*, *12*, 542. https://doi.org/10.3389/fnhum.2018.00542

Sereshkeh, A. R., Yousefi, R., Wong, A. T., & Chau, T. (2018). Online classification of imagined speech using functional near-infrared spectroscopy signals. *Journal of Neural Engineering*, *16*(1), 016005. https://doi.org/10.1088/1741-2552/aae4b9

Soltanlou, M., Artemenko, C., Ehlis, A.-C., Huber, S., Fallgatter, A. J., Dresler, T., & Nuerk, H.-C. (2018). Reduction but no shift in brain activation after arithmetic learning in children: A simultaneous fNIRS-EEG study. *Scientific Reports*, *8*(1), 1–15. https://doi.org/10.1038/s41598-018-20007-x

Stuart, S., Alcock, L., Rochester, L., Vitorio, R., & Pantall, A. (2019). Monitoring multiple cortical regions during walking in young and older adults: Dual-task response and comparison challenges. *International Journal of Psychophysiology*, *135*, 63–72. https://doi.org/10.1016/j.ijpsycho.2018.11.006

Sugiura, L., Hata, M., Matsuba-Kurita, H., Uga, M., Tsuzuki, D., Dan, I., Hagiwara, H., & Homae, F. (2018). Explicit performance in girls and implicit processing in boys: A

simultaneous fNIRS–ERP study on second language syntactic learning in young adolescents. *Frontiers in Human Neuroscience*, *12*, 62. https://doi.org/10.3389/fnhum.2018.00062

Sukal-Moulton, T., de Campos, A. C., Alter, K. E., Huppert, T. J., & Damiano, D. L. (2018). Relationship between sensorimotor cortical activation as assessed by functional near infrared spectroscopy and lower extremity motor coordination in bilateral cerebral palsy. *NeuroImage: Clinical*, *20*, 275–285. https://doi.org/10.1016/j.nicl.2018.07.023

Sulpizio, S., Doi, H., Bornstein, M. H., Cui, J., Esposito, G., & Shinohara, K. (2018). FNIRS reveals enhanced brain activation to female (versus male) infant directed speech (relative to adult directed speech) in young human infants. *Infant Behavior and Development*, *52*, 89–96. https://doi.org/10.1016/j.infbeh.2018.05.009

Sun, P.-P., Tan, F.-L., Zhang, Z., Jiang, Y.-H., Zhao, Y., & Zhu, C.-Z. (2018). Feasibility of functional near-infrared spectroscopy (fNIRS) to investigate the mirror neuron system: An experimental study in a real-life situation. *Frontiers in Human Neuroscience*, *12*, 86. https://doi.org/10.3389/fnhum.2018.00086

Taga, G., Watanabe, H., & Homae, F. (2018a). Spatial variation in the hemoglobin phase of oxygenation and deoxygenation in the developing cortex of infants. *Neurophotonics*, *5*(1), 011017. https://doi.org/10.1117/1.NPh.5.1.011017

Taga, G., Watanabe, H., & Homae, F. (2018b). Developmental changes in cortical sensory processing during wakefulness and sleep. *NeuroImage*, *178*, 519–530. https://doi.org/10.1016/j.neuroimage.2018.05.075

Tang, T. B., & Chan, Y. L. (2018). Functional connectivity analysis on mild alzheimer's disease, mild cognitive impairment and normal aging using fNIRS. *IEEE*, 17–20. https://doi.org/10.1109/EMBC.2018.8512186

Tempest, G. D., & Reiss, A. L. (2019). The utility of functional near-infrared spectroscopy for measuring cortical activity during cycling exercise. *Medicine & Science in Sports & Exercise*, *51*(5), 979–987. https://doi.org/10.1249/MSS.0000000000001875

Teo, W.-P., Goodwill, A. M., Hendy, A. M., Muthalib, M., & Macpherson, H. (2018). Sensory manipulation results in increased dorsolateral prefrontal cortex activation during static postural balance in sedentary older adults: An fNIRS study. *Brain and Behavior*, *8*(10), e01109. https://doi.org/10.1002/brb3.1109

Thumm, P. C., Maidan, I., Brozgol, M., Shustak, S., Gazit, E., Shema Shiratzki, S., Bernad-Elazari, H., Beck, Y., Giladi, N., Hausdorff, J. M., & Mirelman, A. (2018). Treadmill walking reduces pre-frontal activation in patients with Parkinson's disease. *Gait & Posture*, *62*, 384–387. https://doi.org/10.1016/j.gaitpost.2018.03.041

Trakoolwilaiwan, T., Behboodi, B., Lee, J., Kim, K., & Choi, J.-W. (2018). Convolutional neural network for high-accuracy functional near-infrared spectroscopy in a brain–computer interface: Three-class classification of rest, right-, and left-hand motor execution. *Neurophotonics*, *5*(1), 011008. https://doi.org/10.1117/1.NPh.5.1.011008

Trambaiolli, L. R., Biazoli, C. E., Cravo, A. M., Falk, T. H., & Sato, J. R. (2018). Functional near-infrared spectroscopy-based affective neurofeedback: Feedback effect, illiteracy phenomena, and whole-connectivity profiles. *Neurophotonics*, *5*(3), 035009. https://doi.org/10.1117/1.NPh.5.3.035009

Trambaiolli, L. R., Biazoli, C. E., Cravo, A. M., & Sato, J. R. (2018). Predicting affective valence using cortical hemodynamic signals. *Scientific Reports*, *8*(1), 5406. https://doi.org/10.1038/s41598-018-23747-y

Tseng, Y.-L., Lu, C.-F., Wu, S.-M., Shimada, S., Huang, T., & Lu, G.-Y. (2018). A functional near-infrared spectroscopy study of state anxiety and auditory working memory load. *Frontiers in Human Neuroscience*, *12*, 313. https://doi.org/10.3389/fnhum.2018.00313

Ung, W. C., Meriaudeau, F., & Tang, T. B. (2018). Optimizing mental workload by functional near-infrared spectroscopy based dynamic difficulty adjustment. *IEEE*, 1522–1525. https://doi.org/10.1109/EMBC.2018.8512501

van der Kant, A., Biro, S., Levelt, C., & Huijbregts, S. (2018). Negative affect is related to reduced differential neural responses to social and non-social stimuli in 5-to-8-month-old infants: A functional near-infrared spectroscopy-study. *Developmental Cognitive Neuroscience*, *30*, 23–30. https://doi.org/10.1016/j.dcn.2017.12.003

Vassena, E., Gerrits, R., Demanet, J., Verguts, T., & Siugzdaite, R. (2019). Anticipation of a mentally effortful task recruits Dorsolateral Prefrontal Cortex: An fNIRS validation study. *Neuropsychologia*, *123*, 106–115. https://doi.org/10.1016/j.neuropsychologia.2018.04.033

Vasta, R., Cutini, S., Cerasa, A., Gramigna, V., Olivadese, G., Arabia, G., & Quattrone, A. (2018). Physiological aging influence on brain hemodynamic activity during task-switching: A fNIRS study. *Frontiers in Aging Neuroscience*, *9*, 433. https://doi.org/10.3389/fnagi.2017.00433

Verdière, K. J., Roy, R. N., & Dehais, F. (2018). Detecting pilot's engagement using fNIRS connectivity features in an automated vs. Manual landing scenario. *Frontiers in Human Neuroscience*, *12*, 6. https://doi.org/10.3389/fnhum.2018.00006

Vergotte, G., Perrey, S., Muthuraman, M., Janaqi, S., & Torre, K. (2018). Concurrent changes of brain functional connectivity and motor variability when adapting to task constraints. *Frontiers in Physiology*, *9*, 909. https://doi.org/10.3389/fphys.2018.00909

Vitorio, R., Stuart, S., Gobbi, L. T. B., Rochester, L., Alcock, L., & Pantall, A. (2018). Reduced gait variability and enhanced brain activity in older adults with auditory cues: A functional near-infrared spectroscopy study. *Neurorehabilitation and Neural Repair*, *32*(11), 976–987. https://doi.org/10.1177/1545968318805159

Wan, N., Hancock, A. S., Moon, T. K., & Gillam, R. B. (2018). A functional near-infrared spectroscopic investigation of speech production during reading. *Human Brain Mapping*, *39*(3), 1428–1437. https://doi.org/10.1002/hbm.23932

Wang, F., Mao, M., Duan, L., Huang, Y., Li, Z., & Zhu, C. (2018). Intersession instability in fNIRS-based emotion recognition. *IEEE Transactions on Neural Systems and Rehabilitation Engineering*, *26*(7), 1324–1333. https://doi.org/10.1109/TNSRE.2018.2842464

Wang, M.-Y., Lu, F.-M., Hu, Z., Zhang, J., & Yuan, Z. (2018). Optical mapping of prefrontal brain connectivity and activation during emotion anticipation. *Behavioural Brain Research*, *350*, 122–128. https://doi.org/10.1016/j.bbr.2018.04.051

Wang, Z., Chen, L., Yi, W., Gu, B., Liu, S., An, X., Xu, M., Qi, H., He, F., Wan, B., & Ming, D. (2018). Enhancement of cortical activation for motor imagery during BCI-FES training. *IEEE*, 2527–2530. https://doi.org/10.1109/EMBC.2018.8512749

Ward, L. M., Morison, G., Simmers, A. J., & Shahani, U. (2018). Age-related changes in global motion coherence: Conflicting haemodynamic and perceptual responses. *Scientific Reports*, *8*(1), 1–11. https://doi.org/10.1038/s41598-018-27803-5

Weder, S., Zhou, X., Shoushtarian, M., Innes-Brown, H., & McKay, C. (2018). Cortical processing related to intensity of a modulated noise stimulus—A functional near-infrared study. *Journal of the Association for Research in Otolaryngology*, *19*(3), 273–286. https://doi.org/10.1007/s10162-018-0661-0

Weng, W.-C., Chen, J.-C., Lee, C.-Y., Lin, C.-W., Lee, W.-T., Shieh, J.-Y., Wang, C.-C., & Chuang, C.-C. (2018). Cross-section and feasibility study on the non-invasive evaluation of muscle hemodynamic responses in Duchenne muscular dystrophy by using a near-infrared diffuse optical technique. *Biomedical Optics Express*, *9*(10), 4767–4780. https://doi.org/10.1364/BOE.9.004767

Werchan, D. M., Baumgartner, H. A., Lewkowicz, D. J., & Amso, D. (2018). The origins of cortical multisensory dynamics: Evidence from human infants. *Developmental Cognitive Neuroscience*, *34*, 75–81. https://doi.org/10.1016/j.dcn.2018.07.002

Witmer, J., Aeschlimann, E., Metz, A., Troche, S., & Rammsayer, T. (2018). The validity of functional near-infrared spectroscopy recordings of visuospatial working memory processes in humans. *Brain Sciences*, *8*(4), 62. https://doi.org/10.3390/brainsci8040062

Witmer, J. S., Aeschlimann, E. A., Metz, A. J., Troche, S. J., & Rammsayer, T. H. (2018). Functional near-infrared spectroscopy recordings of visuospatial working memory processes. Part II: A replication study in children on sensitivity and mental-ability-induced differences in functional activation. *Brain Sciences*, *8*(8), 152. https://doi.org/10.3390/brainsci8080152

Wolff, W., Bieleke, M., Hirsch, A., Wienbruch, C., Gollwitzer, P. M., & Schüler, J. (2018). Increase in prefrontal cortex oxygenation during static muscular endurance performance is modulated by self-regulation strategies. *Scientific Reports*, *8*(1), 1–10. https://doi.org/10.1038/s41598-018-34009-2

Wriessnegger, S. C., Bauernfeind, G., Kurz, E.-M., Raggam, P., & Müller-Putz, G. R. (2018). Imagine squeezing a cactus: Cortical activation during affective motor imagery measured by functional near-infrared spectroscopy. *Brain and Cognition*, *126*, 13–22. https://doi.org/10.1016/j.bandc.2018.07.006

Wu, S., Li, J., Gao, L., Chen, C., & He, S. (2018). Suppressing systemic interference in fNIRS monitoring of the hemodynamic cortical response to motor execution and imagery. *Frontiers in Human Neuroscience*, *12*, 85. https://doi.org/10.3389/fnhum.2018.00085

Wu, Z., Mazzola, C. A., Catania, L., Owoeye, O., Yaramothu, C., Alvarez, T., Gao, Y., & Li, X. (2018). Altered cortical activation and connectivity patterns for visual attention processing in young adults post-traumatic brain injury: A functional near infrared spectroscopy study. *CNS Neuroscience & Therapeutics*, *24*(6), 539–548. https://doi.org/10.1111/cns.12811

Xie, J., Yang, H., Xia, X., & Yu, S. (2018). The Influence of Medical Professional Knowledge on Empathy for Pain: Evidence From fNIRS. *Frontiers in Psychology*, *9*, 1089. https://doi.org/10.3389/fpsyg.2018.01089

Xue, H., Lu, K., & Hao, N. (2018). Cooperation makes two less-creative individuals turn into a highly-creative pair. *NeuroImage*, *172*, 527–537. https://doi.org/10.1016/j.neuroimage.2018.02.007

Zafar, A., & Hong, K.-S. (2018). Neuronal activation detection using vector phase analysis with dual threshold circles: A functional near-infrared spectroscopy study. *International Journal of Neural Systems*, *28*(10), 1850031. https://doi.org/10.1142/S0129065718500314

Zhang, D., Zhou, Y., & Yuan, J. (2018). Speech Prosodies of Different Emotional Categories Activate Different Brain Regions in Adult Cortex: An fNIRS Study. *Scientific Reports*, *8*(1), 218. https://doi.org/10.1038/s41598-017-18683-2

Zhang, M., Ding, K., Jia, H., & Yu, D. (2018). Brain-to-brain synchronization of the expectation of cooperation behavior: A fNIRS hyperscanning study. *IEEE*, 546–549. https://doi.org/10.1109/EMBC.2018.8512315

Zhang, Y., Meng, T., Hou, Y., Pan, Y., & Hu, Y. (2018). Interpersonal brain synchronization associated with working alliance during psychological counseling. *Psychiatry Research: Neuroimaging*, *282*, 103–109. https://doi.org/10.1016/j.pscychresns.2018.09.007

Zhang, Z., Wang, Y., Zhang, Q., Zhao, W., Chen, X., Zhai, J., Chen, M., Du, B., Deng, X., Ji, F., Wang, C., Xiang, Y., Li, D., Wu, H., Dong, Q., Chen, C., & Li, J. (2019). The effects of CACNA1C gene polymorphism on prefrontal cortex in both schizophrenia patients and healthy controls. *Schizophrenia Research*, *204*, 193–200. https://doi.org/10.1016/j.schres.2018.09.007

Zhao, H., Li, X., Karolis, V., Feng, Y., Niu, H., & Butterworth, B. (2019). Arithmetic learning modifies the functional connectivity of the fronto-parietal network. *Cortex*, *111*, 51–62. https://doi.org/10.1016/j.cortex.2018.07.016

Zhou, X., Seghouane, A.-K., Shah, A., Innes-Brown, H., Cross, W., Litovsky, R., & McKay, C. M. (2018). Cortical speech processing in postlingually deaf adult cochlear implant users,

as revealed by functional near-infrared spectroscopy. *Trends in Hearing*, *22*, 1–18. https://doi.org/10.1177/2331216518786850

Zimeo Morais, G. A., Scholkmann, F., Balardin, J. B., Furucho, R. A., de Paula, R. C. V., Biazoli, C. E., & Sato, J. R. (2018). Non-neuronal evoked and spontaneous hemodynamic changes in the anterior temporal region of the human head may lead to misinterpretations of functional near-infrared spectroscopy signals. *Neurophotonics*, *5*(1), 011002. https://doi.org/10.1117/1.NPh.5.1.011002

Zinszer, B. D., Bayet, L., Emberson, L. L., Raizada, R. D. S., & Aslin, R. N. (2018). Decoding semantic representations from functional near-infrared spectroscopy signals. *Neurophotonics*, *5*(1), 011003. https://doi.org/10.1117/1.NPh.5.1.011003

List of Reviewed fNIRS Studies (2021)

Abujelala, M., Karthikeyan, R., Tyagi, O., Du, J., & Mehta, R. K. (2021). Brain activity-based metrics for assessing learning states in vr under stress among firefighters: An explorative machine learning approach in neuroergonomics. *Brain Sciences*, *11*(7), 885. https://doi.org/10.3390/brainsci11070885

Afzal Khan, M. N., & Hong, K.-S. (2021). Most favorable stimulation duration in the sensorimotor cortex for fNIRS-based BCI. *Biomedical Optics Express*, *12*(10), 5939–5954. https://doi.org/10.1364/BOE.434936

Akın, A. (2021). FNIRS-derived neurocognitive ratio as a biomarker for neuropsychiatric diseases. *Neurophotonics*, *8*(3), 035008. https://doi.org/10.1117/1.NPh.8.3.035008

Alexopoulos, J., Giordano, V., Janda, C., Benavides-Varela, S., Seidl, R., Doering, S., Berger, A., & Bartha-Doering, L. (2021). The duration of intrauterine development influences discrimination of speech prosody in infants. *Developmental Science*, *24*(5), e13110. https://doi.org/10.1111/desc.13110

Alhudhaif, A. (2021). An effective classification framework for brain-computer interface system design based on combining of fNIRS and EEG signals. *PeerJ Computer Science*, *7*, e537. https://doi.org/10.7717/peerj-cs.537

AL-Quraishi, M. S., Elamvazuthi, I., Tang, T. B., Al-Qurishi, M., Adil, S. H., & Ebrahim, M. (2021). Bimodal data fusion of simultaneous measurements of EEG and fNIRS during lower limb movements. *Brain Sciences*, *11*(6), 713. https://doi.org/10.3390/brainsci11060713

Alyan, E., Saad, N. M., & Kamel, N. (2021). Effects of workstation type on mental stress: FNIRS study. *Human Factors: The Journal of the Human Factors and Ergonomics Society*, *63*(7), 1230–1255. https://doi.org/10.1177/0018720820913173

Alyan, E., Saad, N. M., Kamel, N., & Rahman, M. A. (2021). Workplace design-related stress effects on prefrontal cortex connectivity and neurovascular coupling. *Applied Ergonomics*, *96*, 103497. https://doi.org/10.1016/j.apergo.2021.103497

Andreu-Perez, J., Emberson, L. L., Kiani, M., Filippetti, M. L., Hagras, H., & Rigato, S. (2021). Explainable artificial intelligence based analysis for interpreting infant fNIRS data in developmental cognitive neuroscience. *Communications Biology*, *4*(1), 1–13. https://doi.org/10.1038/s42003-021-02534-y

Arora, Y., Walia, P., Hayashibe, M., Muthalib, M., Chowdhury, S. R., Perrey, S., & Dutta, A. (2021). Grey-box modeling and hypothesis testing of functional near-infrared spectroscopy-based cerebrovascular reactivity to anodal high-definition tDCS in healthy humans. *PLOS Computational Biology*, *17*(10), e1009386. https://doi.org/10.1371/journal.pcbi.1009386

Arredondo, M. M., Aslin, R. N., & Werker, J. F. (2022). Bilingualism alters infants' cortical organization for attentional orienting mechanisms. *Developmental Science*, *25*(2), e13172. https://doi.org/10.1111/desc.13172

Asgher, U., Khan, M. J., Asif Nizami, M. H., Khalil, K., Ahmad, R., Ayaz, Y., & Naseer, N. (2021). Motor training using mental workload (mwl) with an assistive soft exoskeleton system: A functional near-infrared spectroscopy (fNIRS) study for brain–machine interface (BMI). *Frontiers in Neurorobotics*, *15*, 605751. https://doi.org/10.3389/fnbot.2021.605751

Azhari, A., Bizzego, A., & Esposito, G. (2021). Father-child dyads exhibit unique inter-subject synchronization during co-viewing of animation video stimuli. *Social Neuroscience*, *16*(5), 522–533. https://doi.org/10.1080/17470919.2021.1970016

Balconi, M., Fronda, G., & Bartolo, A. (2021). Affective, social, and informative gestures reproduction in human interaction: Hyperscanning and brain connectivity. *Journal of Motor Behavior*, *53*(3), 296–315. https://doi.org/10.1080/00222895.2020.1774490

Balters, S., Li, R., Espil, F. M., Piccirilli, A., Liu, N., Gundran, A., Carrion, V. G., Weems, C. F., Cohen, J. A., & Reiss, A. L. (2021). Functional near-infrared spectroscopy brain imaging predicts symptom severity in youth exposed to traumatic stress. *Journal of Psychiatric Research*, *144*, 494–502. https://doi.org/10.1016/j.jpsychires.2021.10.020

Barreto, C., Bruneri, G. de A., Brockington, G., Ayaz, H., & Sato, J. R. (2021). A new statistical approach for fNIRS hyperscanning to predict brain activity of preschoolers' using teacher's. *Frontiers in Human Neuroscience*, *15*, 181. https://doi.org/10.3389/fnhum.2021.622146

Barth, B., Mayer-Carius, K., Strehl, U., Wyckoff, S. N., Haeussinger, F. B., Fallgatter, A. J., & Ehlis, A.-C. (2021). A randomized-controlled neurofeedback trial in adult attention-deficit/hyperactivity disorder. *Scientific Reports*, *11*(1), 1–17. https://doi.org/10.1038/s41598-021-95928-1

Bayet, L., Perdue, K. L., Behrendt, H. F., Richards, J. E., Westerlund, A., Cataldo, J. K., & Nelson, C. A. (2021). Neural responses to happy, fearful and angry faces of varying identities in 5- and 7-month-old infants. *Developmental Cognitive Neuroscience*, *47*, 100882. https://doi.org/10.1016/j.dcn.2020.100882

Belli, V. de, Orcioli-Silva, D., Beretta, V. S., Vitório, R., Zampier, V. C., Nóbrega-Sousa, P., Conceição, N. R. da, & Gobbi, L. T. B. (2021). Prefrontal cortical activity during preferred and fast walking in young and older adults: An fNIRS study. *Neuroscience*, *473*, 81–89. https://doi.org/10.1016/j.neuroscience.2021.08.019

Bello, U. M., Chan, C. C. H., & Winser, S. J. (2021). Task complexity and image clarity facilitate motor and visuo-motor activities in mirror therapy in post-stroke patients. *Frontiers in Neurology*, *12*, 1678. https://doi.org/10.3389/fneur.2021.722846

Belluscio, V., Casti, G., Ferrari, M., Quaresima, V., Sappia, M. S., Horschig, J. M., & Vannozzi, G. (2021). Modifications in prefrontal cortex oxygenation in linear and curvilinear dual task walking: A combined fNIRS and IMUs Study. *Sensors*, *21*(18), 6159. https://doi.org/10.3390/s21186159

Bergen-Cico, D., Grant, T., Hirshfield, L., Razza, R., Costa, M. R., & Kilaru, P. (2021). Using fNIRS to examine neural mechanisms of change associated with mindfulness-based interventions for stress and trauma: Results of a pilot study for women. *Mindfulness*, *12*(9), 2295–2310. https://doi.org/10.1007/s12671-021-01705-6

Bertachini, A. L. L., Januario, G. C., Novi, S. L., Mesquita, R. C., Silva, M. A. R., Andrade, G. M. Q., de Resende, L. M., & de Miranda, D. M. (2021). Hearing brain evaluated using near-infrared spectroscopy in congenital toxoplasmosis. *Scientific Reports*, *11*(1), 1–11. https://doi.org/10.1038/s41598-021-89481-0

Best, T., Clarke, C., Nuzum, N., & Teo, W.-P. (2021). Acute effects of combined Bacopa, American ginseng and whole coffee fruit on working memory and cerebral haemodynamic response of the prefrontal cortex: A double-blind, placebo-controlled

study. *Nutritional Neuroscience*, *24*(11), 873–884. https://doi.org/10.1080/1028415X.2019.1690288

Bettis, A. H., Siciliano, R. E., Rogers, B. P., Ichinose, M., & Compas, B. E. (2021). Neural correlates of distraction and reappraisal in the family context: Associations with symptoms of anxiety and depression in youth. *Child Neuropsychology*, *27*(5), 573–586. https://doi.org/10.1080/09297049.2020.1870675

Bicciato, G., Keller, E., Wolf, M., Brandi, G., Schulthess, S., Friedl, S. G., Willms, J. F., & Narula, G. (2021). Increase in low-frequency oscillations in fNIRS as cerebral response to auditory stimulation with familiar music. *Brain Sciences*, *12*(1), 42. https://doi.org/10.3390/brainsci12010042

Billing, A. D. N., Cooper, R. J., & Scott, S. K. (2021). Pre-SMA activation and the perception of contagiousness and authenticity in laughter sounds. *Cortex*, *143*, 57–68. https://doi.org/10.1016/j.cortex.2021.06.010

Biondi, M., Hirshkowitz, A., Stotler, J., & Wilcox, T. (2021). Cortical activation to social and mechanical stimuli in the infant brain. *Frontiers in Systems Neuroscience*, *15*. https://doi.org/10.3389/fnsys.2021.510030

Blanco, B., Molnar, M., Carreiras, M., Collins-Jones, L. H., Vidal, E., Cooper, R. J., & Caballero-Gaudes, C. (2021). Group-level cortical functional connectivity patterns using fNIRS: Assessing the effect of bilingualism in young infants. *Neurophotonics*, *8*(2), 025011. https://doi.org/10.1117/1.NPh.8.2.025011

Blum, L., Rosenbaum, D., Röben, B., Dehnen, K., Maetzler, W., Suenkel, U., Fallgatter, A. J., Ehlis, A.-C., & Metzger, F. G. (2021). Age-related deterioration of performance and

increase of cortex activity comparing time- versus item-controlled fNIRS measurement. *Scientific Reports*, *11*(1), 1–13. https://doi.org/10.1038/s41598-021-85762-w

Borrell, J. A., Copeland, C., Lukaszek, J. L., Fraser, K., & Zuniga, J. M. (2021). Use-dependent prosthesis training strengthens contralateral hemodynamic brain responses in a young adult with upper limb reduction deficiency: A case report. *Frontiers in Neuroscience*, *15*. https://doi.org/10.3389/fnins.2021.693138

Cai, Z., Uji, M., Aydin, Ü., Pellegrino, G., Spilkin, A., Delaire, É., Abdallah, C., Lina, J., & Grova, C. (2021). Evaluation of a personalized functional near infra-red optical tomography workflow using maximum entropy on the mean. *Human Brain Mapping*, *42*(15), 4823–4843. https://doi.org/10.1002/hbm.25566

Cañigueral, R., Zhang, X., Noah, J. A., Tachtsidis, I., Hamilton, A. F. de C., & Hirsch, J. (2021). Facial and neural mechanisms during interactive disclosure of biographical information. *NeuroImage*, *226*, 117572. https://doi.org/10.1016/j.neuroimage.2020.117572

Carius, D., Kenville, R., Maudrich, D., Riechel, J., Lenz, H., & Ragert, P. (2021). Cortical processing during table tennis—An fNIRS study in experts and novices. *European Journal of Sport Science*, 1–11. https://doi.org/10.1080/17461391.2021.1953155

Chan, A. S., Lee, T., Hamblin, M. R., & Cheung, M. (2021a). Photobiomodulation enhances memory processing in older adults with mild cognitive impairment: A functional near-infrared spectroscopy study. *Journal of Alzheimer's Disease*, *83*(4), 1471–1480. https://doi.org/10.3233/JAD-201600

Chan, A. S., Lee, T., Hamblin, M. R., & Cheung, M. (2021b). Photoneuromodulation makes a difficult cognitive task less arduous. *Scientific Reports*, *11*(1), 1–9. https://doi.org/10.1038/s41598-021-93228-2

Chang, P.-W., Lu, C.-F., Chang, S.-T., & Tsai, P.-Y. (2022). Functional Near-infrared spectroscopy as a target navigator for rTMS modulation in patients with hemiplegia: A randomized control study. *Neurology and Therapy*, *11*(1), 103–121. https://doi.org/10.1007/s40120-021-00300-0

Chao, J., Zheng, S., Wu, H., Wang, D., Zhang, X., Peng, H., & Hu, B. (2021). FNIRS evidence for distinguishing patients with major depression and healthy controls. *IEEE Transactions on Neural Systems and Rehabilitation Engineering*, *29*, 2211–2221. https://doi.org/10.1109/TNSRE.2021.3115266

Chen, C.-H., Shyu, K.-K., Lu, C.-K., Jao, C.-W., & Lee, P.-L. (2021). Classification of prefrontal cortex activity based on functional near-infrared spectroscopy data upon olfactory stimulation. *Brain Sciences*, *11*(6), 701. https://doi.org/10.3390/brainsci11060701

Chen, T., Zhao, C., Pan, X., Qu, J., Wei, J., Li, C., Liang, Y., & Zhang, X. (2021). Decoding different working memory states during an operation span task from prefrontal fNIRS signals. *Biomedical Optics Express*, *12*(6), 3495–3511. https://doi.org/10.1364/BOE.426731

Cheng, X., Zhu, Y., Hu, Y., Zhou, X., Pan, Y., & Hu, Y. (2022). Integration of social status and trust through interpersonal brain synchronization. *NeuroImage*, *246*, 118777. https://doi.org/10.1016/j.neuroimage.2021.118777

Chou, P.-H., Yao, Y.-H., Zheng, R.-X., Liou, Y.-L., Liu, T.-T., Lane, H.-Y., Yang, A. C., & Wang, S.-C. (2021). Deep neural network to differentiate brain activity between patients with first-episode schizophrenia and healthy individuals: A multi-channel near infrared spectroscopy study. *Frontiers in Psychiatry*, *12*, 655292. https://doi.org/10.3389/fpsyt.2021.655292

Clark, D. J., Chatterjee, S. A., Skinner, J. W., Lysne, P. E., Sumonthee, C., Wu, S. S., Cohen, R. A., Rose, D. K., & Woods, A. J. (2021). Combining frontal tDCS with walking rehabilitation to enhance mobility and executive function: A pilot clinical trial. *Neuromodulation: Journal of the International Neuromodulation Society*, *24*(5), 950. https://doi.org/10.1111/ner.13250

Clark, D. J., Rose, D. K., Butera, K. A., Hoisington, B., DeMark, L., Chatterjee, S. A., Hawkins, K. A., Otzel, D. M., Skinner, J. W., Christou, E. A., Wu, S. S., & Fox, E. J. (2021). Rehabilitation with accurate adaptability walking tasks or steady state walking: A randomized clinical trial in adults post-stroke. *Clinical Rehabilitation*, *35*(8), 1196–1206. https://doi.org/10.1177/02692155211001682

Coelho, D. B., Bazán, P. R., Zimeo Morais, G. A., Balardin, J. B., Batista, A. X., de Oliveira, C. E. N., Los Angeles, E., Bernardo, C., Sato, J. R., & de Lima-Pardini, A. C. (2021). Frontal hemodynamic response during step initiation under cognitive conflict in older and young healthy people. *The Journals of Gerontology: Series A*, *76*(2), 216–223. https://doi.org/10.1093/gerona/glaa125

Collett, J., Fleming, M. K., Meester, D., Al-Yahya, E., Wade, D. T., Dennis, A., Salvan, P., Meaney, A., Cockburn, J., Dawes, J., Johansen-Berg, H., & Dawes, H. (2021). Dual-task walking and automaticity after Stroke: Insights from a secondary analysis and imaging sub-study of a randomised controlled trial. *Clinical Rehabilitation*, *35*(11), 1599–1610. https://doi.org/10.1177/02692155211017360

Collins-Jones, L. H., Cooper, R. J., Bulgarelli, C., Blasi, A., Katus, L., McCann, S., Mason, L., Mbye, E., Touray, E., Ceesay, M., Moore, S. E., Lloyd-Fox, S., & Elwell, C. E. (2021). Longitudinal infant fNIRS channel-space analyses are robust to variability parameters at

the group-level: An image reconstruction investigation. *NeuroImage*, *237*, 118068. https://doi.org/10.1016/j.neuroimage.2021.118068

Conceição, N. R., Gobbi, L. T. B., Nóbrega-Sousa, P., Orcioli-Silva, D., Beretta, V. S., Lirani-Silva, E., Okano, A. H., & Vitório, R. (2021). Aerobic exercise combined with transcranial direct current stimulation over the prefrontal cortex in parkinson disease: Effects on cortical activity, gait, and cognition. *Neurorehabilitation and Neural Repair, 35*(8), 717–728. https://doi.org/10.1177/15459683211019344

Cong, L., Miyaguchi, H., & Ishizuki, C. (2021). Comparison of activation in the prefrontal cortex of native speakers of mandarin by ability of japanese as a second language using a novel speaking task. *Healthcare*, *9*(4), 412. https://doi.org/10.3390/healthcare9040412

Cooney, C., Folli, R., & Coyle, D. H. (2021). A bimodal deep learning architecture for EEG-fNIRS decoding of overt and imagined speech. *IEEE Transactions on Biomedical Engineering*, 1–1. https://doi.org/10.1109/TBME.2021.3132861

Copeland, C., Mukherjee, M., Wang, Y., Fraser, K., & Zuniga, J. M. (2021). Changes in sensorimotor cortical activation in children using prostheses and prosthetic simulators. *Brain Sciences*, *11*(8), 991. https://doi.org/10.3390/brainsci11080991

Csipo, T., Lipecz, A., Mukli, P., Bahadli, D., Abdulhussein, O., Owens, C. D., Tarantini, S., Hand, R. A., Yabluchanska, V., Kellawan, J. M., Sorond, F., James, J. A., Csiszar, A., Ungvari, Z. I., & Yabluchanskiy, A. (2021). Increased cognitive workload evokes greater neurovascular coupling responses in healthy young adults. *PLOS ONE*, *16*(5), e0250043. https://doi.org/10.1371/journal.pone.0250043

Csipo, T., Lipecz, A., Owens, C., Mukli, P., Perry, J. W., Tarantini, S., Balasubramanian, P., Nyúl-Tóth, Á., Yabluchanska, V., Sorond, F. A., Kellawan, J. M., Purebl, G., Sonntag,

W. E., Csiszar, A., Ungvari, Z., & Yabluchanskiy, A. (2021). Sleep deprivation impairs cognitive performance, alters task-associated cerebral blood flow and decreases cortical neurovascular coupling-related hemodynamic responses. *Scientific Reports*, *11*(1), 1–13. https://doi.org/10.1038/s41598-021-00188-8

Curzel, F., Brigadoi, S., & Cutini, S. (2021). fNIRS & e-drum: An ecological approach to monitor hemodynamic and behavioural effects of rhythmic auditory cueing training. *Brain and Cognition*, *151*, 105753. https://doi.org/10.1016/j.bandc.2021.105753

Da, C., Jj, L., Metting, Z., Se, R., Jm, S., Jwj, E., & van der Naalt, J. (2021). The feasibility of fNIRS as a diagnostic tool for pediatric TBI: A pilot study. *European Journal of Paediatric Neurology*, *30*, 22–24. https://doi.org/10.1016/j.ejpn.2020.12.008

Dagan, M., Herman, T., Bernad-Elazari, H., Gazit, E., Maidan, I., Giladi, N., Mirelman, A., Manor, B., & Hausdorff, J. M. (2021). Dopaminergic therapy and prefrontal activation during walking in individuals with Parkinson's disease: Does the levodopa overdose hypothesis extend to gait? *Journal of Neurology*, *268*(2), 658–668. https://doi.org/10.1007/s00415-020-10089-x

Dahan, A., Dubnov, Y. A., Popkov, A. Y., Gutman, I., & Probolovski, H. G. (2021). Brief report: Classification of autistic traits according to brain activity recoded by fNIRS using ε-complexity coefficients. *Journal of Autism and Developmental Disorders*, *51*(9), 3380–3390. https://doi.org/10.1007/s10803-020-04793-w

de Rond, V., Orcioli-Silva, D., Dijkstra, B. W., Orban de Xivry, J.-J., Pantall, A., & Nieuwboer, A. (2021). Compromised brain activity with age during a game-like dynamic balance task: Single- vs. dual-task performance. *Frontiers in Aging Neuroscience*, *13*. https://doi.org/10.3389/fnagi.2021.657308

Defenderfer, J., Forbes, S., Wijeakumar, S., Hedrick, M., Plyler, P., & Buss, A. T. (2021). Frontotemporal activation differs between perception of simulated cochlear implant speech and speech in background noise: An image-based fNIRS study. *NeuroImage*, *240*, 118385. https://doi.org/10.1016/j.neuroimage.2021.118385

Deligani, R. J., Borgheai, S. B., McLinden, J., & Shahriari, Y. (2021). Multimodal fusion of EEG-fNIRS: A mutual information-based hybrid classification framework. *Biomedical Optics Express*, *12*(3), 1635–1650. https://doi.org/10.1364/BOE.413666

Derbie, A. Y., Chau, B., Lam, B., Fang, Y., Ting, K.-H., Wong, C. Y. H., Tao, J., Chen, L., & Chan, C. C. H. (2021). Cortical hemodynamic response associated with spatial coding: A near-infrared spectroscopy study. *Brain Topography*, *34*(2), 207–220. https://doi.org/10.1007/s10548-021-00821-9

Dieffenbach, M. C., Gillespie, G. S. R., Burns, S. M., McCulloh, I. A., Ames, D. L., Dagher, M. M., Falk, E. B., & Lieberman, M. D. (2021). Neural reference groups: A synchrony-based classification approach for predicting attitudes using fNIRS. *Social Cognitive and Affective Neuroscience*, *16*(1–2), 117–128. https://doi.org/10.1093/scan/nsaa115

Ding, G., Mohr, K. A. J., Orellana, C. I., Hancock, A. S., Juth, S., Wada, R., & Gillam, R. B. (2021). Use of functional near infrared spectroscopy to assess syntactic processing by monolingual and bilingual adults and children. *Frontiers in Human Neuroscience*, *15*(8). https://doi.org/10.3389/fnhum.2021.621025

Ding, K., Li, C., Jia, H., Zhang, M., & Yu, D. (2021). Is left-behind a real reason for children's social cognition deficit? An fNIRS study on the effect of social interaction on left-behind preschooler's prefrontal activation. *PLOS ONE*, *16*(9), e0254010. https://doi.org/10.1371/journal.pone.0254010

Ding, K., Li, C., Li, Y., Wang, H., & Yu, D. (2021). The effect of socioeconomic disparities on prefrontal activation in initiating joint attention: A Functional near-infrared spectroscopy evidence from two socioeconomic status groups. *Frontiers in Human Neuroscience, 15*. https://doi.org/10.3389/fnhum.2021.741872

Ding, K., Li, C., Wang, J., & Yu, D. (2021). Negative affective processing is associated with cognitive control in early childhood: An fNIRS study. *IEEE*, 3423–3426. https://doi.org/10.1109/EMBC46164.2021.9629783

Ding, K., Wang, H., Li, C., Liu, F., & Yu, D. (2021). Decreased right prefrontal synchronization strength and asymmetry during joint attention in the left-behind children: A functional near-infrared spectroscopy study. *Frontiers in Physiology*, *12*, 759788. https://doi.org/10.3389/fphys.2021.759788

Ding, P., Wang, F., Li, S., Zhang, W., Li, H., Chen, Z., Zhao, L., Gong, A., & Fu, Y. (2021). Monitoring and evaluation of emotion regulation by aerobic exercise and motor imagery based on functional near-infrared spectroscopy. *Frontiers in Computational Neuroscience*, *15*. https://doi.org/10.3389/fncom.2021.759360

Dong, S.-Y., Choi, J., Park, Y., Baik, S. Y., Jung, M., Kim, Y., & Lee, S.-H. (2021). Prefrontal functional connectivity during the verbal fluency task in patients with major depressive disorder: A functional near-infrared spectroscopy study. *Frontiers in Psychiatry*, *12*. https://doi.org/10.3389/fpsyt.2021.659814

Duan, L., Ai, H., Yang, L., Xu, L., & Xu, P. (2021). Gender differences in transnational brand purchase decision toward mixed culture and original culture advertisements: An fNIRS study. *Frontiers in Psychology*, *12*. https://doi.org/10.3389/fpsyg.2021.654360

Dybvik, H., & Steinert, M. (2021). Real-world fNIRS brain activity measurements during ashtanga vinyasa yoga. *Brain Sciences*, *11*(6), 742. https://doi.org/10.3390/brainsci11060742

Erdoğan, S. B., Yükselen, G., Yegül, M. M., Usanmaz, R., Kıran, E., Derman, O., & Akın, A. (2021). Identification of impulsive adolescents with a functional near infrared spectroscopy (fNIRS) based decision support system. *Journal of Neural Engineering*, *18*(5), 056043. https://doi.org/10.1088/1741-2552/ac23bb

Fan, S., Blanco-Davis, E., Zhang, J., Bury, A., Warren, J., Yang, Z., Yan, X., Wang, J., & Fairclough, S. (2021). The role of the prefrontal cortex and functional connectivity during maritime operations: An fNIRS study. *Brain and Behavior*, *11*(1), e01910. https://doi.org/10.1002/brb3.1910

Fatakdawala, I., Ayaz, H., Safati, A. B., Sakib, M. N., & Hall, P. A. (2021). Effects of prefrontal theta burst stimulation on neuronal activity and subsequent eating behavior: An interleaved rTMS and fNIRS study. *Social Cognitive and Affective Neuroscience*, *16*(9), 1021. https://doi.org/10.1093/scan/nsab023

Feng, K., Law, S., Ravindran, N., Chen, G., Ma, X., Bo, X., Zhang, X.-Q., Shen, C., Li, J., Wang, Y., Liu, X., Sun, J., Hu, S., & Liu, P. (2021). Differentiating between bipolar and unipolar depression using prefrontal activation patterns: Promising results from functional near infrared spectroscopy (fNIRS) findings. *Journal of Affective Disorders*, *281*, 476–484. https://doi.org/10.1016/j.jad.2020.12.048

Feng, Y. X., Kiguchi, M., Ung, W. C., Dass, S. C., Mohd Hani, A. F., Tang, T. B., & Ho, E. T. W. (2021). Working memory performance under a negative affect is more susceptible to

higher cognitive workloads with different neural haemodynamic correlates. *Brain Sciences*, *11*(7), 935. https://doi.org/10.3390/brainsci11070935

Fu, Y., Chen, R., Gong, A., Qian, Q., Ding, N., Zhang, W., Su, L., & Zhao, L. (2021). Recognition of flexion and extension imagery involving the right and left arms based on deep belief network and functional near-infrared spectroscopy. *Journal of Healthcare Engineering*, 1–11. https://doi.org/10.1155/2021/5533565

Fujihara, H., Megumi, A., & Yasumura, A. (2021). The acute effect of moderate-intensity exercise on inhibitory control and activation of prefrontal cortex in younger and older adults. *Experimental Brain Research*, *239*(6), 1765–1778. https://doi.org/10.1007/s00221-021-06086-9

Fujii, M., Tanigo, K., Yamamoto, H., Kikugawa, K., Shirakawa, M., Ohgushi, M., & Chin, T. (2021). A case of dysgraphia after cerebellar infarction where functional NIRS guided the task aimed at activating the hypoperfused region. *Case Reports in Neurological Medicine*. https://doi.org/10.1155/2021/6612541

Fukuda, K., Wakamatsu, Y., & Fujii, M. (2021). Application of depth selectivity filter to brain function measurement by fNIRS. *IEEE*, 3787–3790. https://doi.org/10.1109/EMBC46164.2021.9629723

Galati, A., Schoppa, R., & Lu, A. (2021). Exploring the sensemaking process through interactions and fnirs in immersive visualization. *IEEE Transactions on Visualization and Computer Graphics*, *27*(5), 2714–2724. https://doi.org/10.1109/TVCG.2021.3067693

Galli, A., Brigadoi, S., Giorgi, G., Sparacino, G., & Narduzzi, C. (2021). Accurate hemodynamic response estimation by removal of stimulus-evoked superficial response in fNIRS

signals. *Journal of Neural Engineering*, *18*(3), 036019. https://doi.org/10.1088/1741-2552/abdb3a

Galoyan, T., Betts, K., Abramian, H., Reddy, P., Izzetoglu, K., & Shewokis, P. A. (2021). Examining mental workload in a spatial navigation transfer game via functional near infrared spectroscopy. *Brain Sciences*, *11*(1), 45. https://doi.org/10.3390/brainsci11010045

Gamliel, H. N., Nevat, M., Probolovski, H. Z. G., Karklinsky, M., Han, S., & Shamay-Tsoory, S. G. (2021). Inter-group conflict affects inter-brain synchrony during synchronized movements. *NeuroImage*, *245*, 118661. https://doi.org/10.1016/j.neuroimage.2021.118661

Gao, Y., Cavuoto, L., Dutta, A., Kruger, U., Yan, P., Nemani, A., Norfleet, J. E., Makled, B. A., Silvestri, J., Schwaitzberg, S., Intes, X., & De, S. (2021). Decreasing the Surgical errors by neurostimulation of primary motor cortex and the associated brain activation via neuroimaging. *Frontiers in Neuroscience*, *15*, 265. https://doi.org/10.3389/fnins.2021.651192

Gemignani, J., & Gervain, J. (2021). Comparing different pre-processing routines for infant fNIRS data. *Developmental Cognitive Neuroscience*, *48*, 100943. https://doi.org/10.1016/j.dcn.2021.100943

Gilmore, N., Yücel, M. A., Li, X., Boas, D. A., & Kiran, S. (2021). Investigating language and domain-general processing in neurotypicals and individuals with aphasia—A functional near-infrared spectroscopy pilot study. *Frontiers in Human Neuroscience*, *15*, 543. https://doi.org/10.3389/fnhum.2021.728151

Giordano, V., Alexopoulos, J., Spagna, A., Benavides-Varela, S., Peganc, K., Kothgassner, O. D., Klebermass-Schrehof, K., Olischar, M., Berger, A., & Bartha-Doering, L. (2021). Accent discrimination abilities during the first days of life: An fNIRS study. *Brain and Language*, *223*, 105039. https://doi.org/10.1016/j.bandl.2021.105039

Giorjiani, G. M., Biazoli, C. E., & Caetano, M. S. (2021). Differences in perceived durations between plausible biological and non-biological stimuli. *Experimental Brain Research*, *239*(1), 161–173. https://doi.org/10.1007/s00221-020-05904-w

Goenarjo, R., Dupuy, O., Fraser, S., Berryman, N., Perrochon, A., & Bosquet, L. (2021). Cardiorespiratory fitness and prefrontal cortex oxygenation during Stroop task in older males. *Physiology & Behavior*, *242*, 113621. https://doi.org/10.1016/j.physbeh.2021.113621

Goodman, S. P. J., & Marino, F. E. (2021). Thirst perception exacerbates objective mental fatigue. *Neuropsychologia*, *150*, 107686. https://doi.org/10.1016/j.neuropsychologia.2020.107686

Grässler, B., Herold, F., Dordevic, M., Gujar, T. A., Darius, S., Böckelmann, I., Müller, N. G., & Hökelmann, A. (2021). Multimodal measurement approach to identify individuals with mild cognitive impairment: Study protocol for a cross-sectional trial. *BMJ Open*, *11*(5), e046879. https://doi.org/10.1136/bmjopen-2020-046879

Gu, X., Yang, B., Gao, S., Yan, L. F., Xu, D., & Wang, W. (2021). Prefrontal fNIRS-based clinical data analysis of brain functions in individuals abusing different types of drugs. *Journal of Biomedical Semantics*, *12*(1), 1–14. https://doi.org/10.1186/s13326-021-00256-y

Guérin, S. M. R., Vincent, M. A., Karageorghis, C. I., & Delevoye-Turrell, Y. N. (2021). Effects of motor tempo on frontal brain activity: An fNIRS study. *NeuroImage*, *230*, 117597. https://doi.org/10.1016/j.neuroimage.2020.117597

Haberstumpf, S., Seidel, A., Lauer, M., Polak, T., Deckert, J., & Herrmann, M. J. (2022). Reduced parietal activation in participants with mild cognitive impairments during visual-spatial processing measured with functional near-infrared spectroscopy. *Journal of Psychiatric Research*, *146*, 31–42. https://doi.org/10.1016/j.jpsychires.2021.12.021

Hashmi, S., Vanderwert, R. E., Paine, A. L., & Gerson, S. A. (2022). Doll play prompts social thinking and social talking: Representations of internal state language in the brain. *Developmental Science*, *25*(2), e13163. https://doi.org/10.1111/desc.13163

He, Y., Hu, Y., Yang, Y., Li, D., & Hu, Y. (2021). Optical mapping of brain activity underlying directionality and its modulation by expertise in mandarin/english interpreting. *Frontiers in Human Neuroscience*, *15*. https://doi.org/10.3389/fnhum.2021.649578

Heiland, E. G., Tarassova, O., Fernström, M., English, C., Ekblom, Ö., & Ekblom, M. M. (2021). Frequent, short physical activity breaks reduce prefrontal cortex activation but preserve working memory in middle-aged adults: ABBaH study. *Frontiers in Human Neuroscience*, *15*, 533. https://doi.org/10.3389/fnhum.2021.719509

Herold, F., Behrendt, T., Törpel, A., Hamacher, D., Müller, N. G., & Schega, L. (2021). Cortical hemodynamics as a function of handgrip strength and cognitive performance: A cross-sectional fNIRS study in younger adults. *BMC Neuroscience*, *22*(1), 1–16. https://doi.org/10.1186/s12868-021-00615-6

Hirsch, J., Tiede, M., Zhang, X., Noah, J. A., Salama-Manteau, A., & Biriotti, M. (2021). Interpersonal agreement and disagreement during face-to-face dialogue: An fNIRS

investigation. *Frontiers in Human Neuroscience*, *14*, 601. https://doi.org/10.3389/fnhum.2020.606397

Hitomi, T., Gerrits, R., & Hartsuiker, R. J. (2021). Using functional near-infrared spectroscopy to study word production in the brain: A picture-word interference study. *Journal of Neurolinguistics*, *57*, 100957. https://doi.org/10.1016/j.jneuroling.2020.100957

Ho, R. C., Sharma, V. K., Tan, B. Y. Q., Ng, A. Y. Y., Lui, Y.-S., Husain, S. F., Ho, C. S., Tran, B. X., Pham, Q.-H., McIntyre, R. S., & Chan, A. C. Y. (2021). Comparison of brain activation patterns during olfactory stimuli between recovered covid-19 patients and healthy controls: A functional near-infrared spectroscopy (fNIRS) study. *Brain Sciences*, *11*(8), 968. https://doi.org/10.3390/brainsci11080968

Hoang, I., Ranchet, M., Cheminon, M., Derollepot, R., Devos, H., Perrey, S., Luauté, J., Danaila, T., & Paire-Ficout, L. (2022). An intensive exercise-based training program reduces prefrontal activity during usual walking in patients with Parkinson's disease. *Clinical Parkinsonism & Related Disorders*, *6*, 100128. https://doi.org/10.1016/j.prdoa.2021.100128

Hofmann, A., Rosenbaum, D., Int-Veen, I., Ehlis, A.-C., Brockmann, K., Dehnen, K., von Thaler, A.-K., Berg, D., Fallgatter, A. J., & Metzger, F. G. (2021). Abnormally reduced frontal cortex activity during Trail-Making-Test in prodromal parkinson's disease–a fNIRS study. *Neurobiology of Aging*, *105*, 148–158. https://doi.org/10.1016/j.neurobiolaging.2021.04.014

Holtzer, R., Ross, D., O'Brien, C., Izzetoglu, M., & Wagshul, M. E. (2021). Cognitive reserve moderates the efficiency of prefrontal cortex activation patterns of gait in older adults. *The Journals of Gerontology: Series A*. https://doi.org/10.1093/gerona/glab288

Hou, X., Xiao, X., Gong, Y., Li, Z., Chen, A., & Zhu, C. (2021). Functional near-infrared spectroscopy neurofeedback enhances human spatial memory. *Frontiers in Human Neuroscience*, *15*. https://doi.org/10.3389/fnhum.2021.681193

Hoyniak, C. P., Quiñones-Camacho, L. E., Camacho, M. C., Chin, J. H., Williams, E. M., Wakschlag, L. S., & Perlman, S. B. (2021). Adversity is linked with decreased parent-child behavioral and neural synchrony. *Developmental Cognitive Neuroscience*, *48*, 100937. https://doi.org/10.1016/j.dcn.2021.100937

Hu, M., Zeng, N., Gu, Z., Zheng, Y., Xu, K., Xue, L., Leng, L., Lu, X., Shen, Y., & Huang, J. (2021). Short-term high-intensity interval exercise promotes motor cortex plasticity and executive function in sedentary females. *Frontiers in Human Neuroscience*, *15*, 209. https://doi.org/10.3389/fnhum.2021.620958

Hu, S., Li, X., Law, S., Shen, C., Yao, G., Zhang, X., Li, J., Chen, G., Xu, B., Liu, X., Ma, X., Feng, K., & Liu, P. (2021). Prefrontal cortex alterations in major depressive disorder, generalized anxiety disorder and their comorbidity during a verbal fluency task assessed by multi-channel near-infrared spectroscopy. *Psychiatry Research*, *306*, 114229. https://doi.org/10.1016/j.psychres.2021.114229

Huhn, A. S., Brooner, R. K., Sweeney, M. M., Antoine, D., Hammond, A. S., Ayaz, H., & Dunn, K. E. (2021). The association of prefrontal cortex response during a natural reward cue-reactivity paradigm, anhedonia, and demoralization in persons maintained on methadone. *Addictive Behaviors*, *113*, 106673. https://doi.org/10.1016/j.addbeh.2020.106673

Husain, S. F., McIntyre, R. S., Tang, T.-B., Abd Latif, M. H., Tran, B. X., Linh, V. G., Thao, T. P. N., Ho, C. S., & Ho, R. C. (2021). Functional near-infrared spectroscopy during the

verbal fluency task of English-Speaking adults with mood disorders: A preliminary study. *Journal of Clinical Neuroscience*, *94*, 94–101. https://doi.org/10.1016/j.jocn.2021.10.009

Husain, S. F., Tang, T.-B., Tam, W. W., Tran, B. X., Ho, C. S., & Ho, R. C. (2021). Cortical haemodynamic response during the verbal fluency task in patients with bipolar disorder and borderline personality disorder: A preliminary functional near-infrared spectroscopy study. *BMC Psychiatry*, *21*(1), 1–10. https://doi.org/10.1186/s12888-021-03195-1

Immink, M. A., Pointon, M., Wright, D. L., & Marino, F. E. (2021). Prefrontal Cortex activation during motor sequence learning under interleaved and repetitive practice: A two-channel near-infrared spectroscopy study. *Frontiers in Human Neuroscience*, *15*, 229. https://doi.org/10.3389/fnhum.2021.644968

Izzetoglu, K., Aksoy, M. E., Agrali, A., Kitapcioglu, D., Gungor, M., & Simsek, A. (2021). Studying brain activation during skill acquisition via robot-assisted surgery training. *Brain Sciences*, *11*(7), 937. https://doi.org/10.3390/brainsci11070937

Jackson, E. S., Wijeakumar, S., Beal, D. S., Brown, B., Zebrowski, P. M., & Spencer, J. P. (2021). Speech planning and execution in children who stutter: Preliminary findings from a fNIRS investigation. *Journal of Clinical Neuroscience*, *91*, 32–42. https://doi.org/10.1016/j.jocn.2021.06.018

Jalalvandi, M., Riyahi Alam, N., Sharini, H., Hashemi, H., & Nadimi, M. (2021). Brain cortical activation during imagining of the wrist movement using functional near infrared spectroscopy (fNIRS). *Journal of Biomedical Physics and Engineering*, *11*(5), 583. https://doi.org/10.31661/jbpe.v0i0.1051

Jang, S., Choi, J., Oh, J., Yeom, J., Hong, N., Lee, N., Kwon, J. H., Hong, J., Kim, J., & Kim, E. (2021). Use of virtual reality working memory task and functional near-infrared

spectroscopy to assess brain hemodynamic responses to methylphenidate in ADHD children. *Frontiers in Psychiatry*, *11*, 1644. https://doi.org/10.3389/fpsyt.2020.564618

Jasińska, K. K., Shuai, L., Lau, A. N. L., Frost, S., Landi, N., & Pugh, K. R. (2021). Functional connectivity in the developing language network in 4-year-old children predicts future reading ability. *Developmental Science*, *24*(2), e13041. https://doi.org/10.1111/desc.13041

Jia, G., Liu, G., & Niu, H. (2022). Hemispheric lateralization of visuospatial attention is independent of language production on right-handers: Evidence from functional near-infrared spectroscopy. *Frontiers in Neurology*, *12*, 2582. https://doi.org/10.3389/fneur.2021.784821

Jian, C., Deng, L., Liu, H., Yan, T., Wang, X., & Song, R. (2021). Modulating and restoring inter-muscular coordination in stroke patients using two-dimensional myoelectric computer interface: A cross-sectional and longitudinal study. *Journal of Neural Engineering*, *18*(3), 036005. https://doi.org/10.1088/1741-2552/abc29a

Jian, C., Liu, H., Deng, L., Wang, X., Yan, T., & Song, R. (2021). Stroke-induced alteration in multi-layer information transmission of cortico-motor system during elbow isometric contraction modulated by myoelectric-controlled interfaces. *Journal of Neural Engineering*, *18*(4), 0460e1. https://doi.org/10.1088/1741-2552/ac18ae

Jiang, D., Liu, Z., & Sun, G. (2021). The effect of yoga meditation practice on young adults' inhibitory control: An fNIRS study. *Frontiers in Human Neuroscience*, *15*, 539. https://doi.org/10.3389/fnhum.2021.725233

Jiang, X., Zhou, C., Ao, N., Gu, W., Li, J., & Chen, Y. (2021). Scarcity mindset neuro network decoding with reward: A tree-based model and functional near-infrared spectroscopy study. *Frontiers in Human Neuroscience*, *15*. https://doi.org/10.3389/fnhum.2021.736415

Joo, S. Y., Cho, Y. S., Lee, K. J., Lee, S. Y., & Seo, C. H. (2021). Frontal lobe oxyhemoglobin levels in patients with lower extremity burns assessed using a functional near-Infrared spectroscopy device during usual walking: A pilot study. *Computer Methods in Biomechanics and Biomedical Engineering*, *24*(2), 115–121. https://doi.org/10.1080/10255842.2020.1812583

Kaimal, G., Carroll-Haskins, K., Topoglu, Y., Ramakrishnan, A., Arslanbek, A., & Ayaz, H. (2021). Exploratory fNIRS assessment of differences in activation in virtual reality visual self-expression including with a fragrance stimulus. *Art Therapy*, *39*(3), 128–137. https://doi.org/10.1080/07421656.2021.1957341

Karunakaran, K. D., Ji, K., Chen, D. Y., Chiaravalloti, N. D., Niu, H., Alvarez, T. L., & Biswal, B. B. (2021). Relationship between age and cerebral hemodynamic response to breath holding: A functional near-infrared spectroscopy study. *Brain Topography*, *34*(2), 154–166. https://doi.org/10.1007/s10548-021-00818-4

Kassab, A., Hinnoutondji Toffa, D., Robert, M., Lesage, F., Peng, K., & Khoa Nguyen, D. (2021). Hemodynamic changes associated with common EEG patterns in critically ill patients: Pilot results from continuous EEG-fNIRS study. *NeuroImage: Clinical*, *32*, 102880. https://doi.org/10.1016/j.nicl.2021.102880

Kelley, M. S., Noah, J. A., Zhang, X., Scassellati, B., & Hirsch, J. (2021). Comparison of human social brain activity during eye-contact with another human and a humanoid robot. *Frontiers in Robotics and AI*, *7*, 209. https://doi.org/10.3389/frobt.2020.599581

Khaksari, K., Smith, E. G., Miguel, H. O., Zeytinoglu, S., Fox, N., & Gandjbakhche, A. H. (2022). An fNIRS study of brain lateralization during observation and execution of a fine motor task. *Frontiers in Human Neuroscience*, *15*. https://doi.org/10.3389/fnhum.2021.798870

Khan, H., Noori, F. M., Yazidi, A., Uddin, M. Z., Khan, M. N. A., & Mirtaheri, P. (2021). Classification of individual finger movements from right hand using fNIRS signals. *Sensors*, *21*(23), 7943. https://doi.org/10.3390/s21237943

Kia, K., Hwang, J., Kim, I.-S., Ishak, H., & Kim, J. H. (2021). The effects of target size and error rate on the cognitive demand and stress during augmented reality interactions. *Applied Ergonomics*, *97*, 103502. https://doi.org/10.1016/j.apergo.2021.103502

Kim, E., Yu, J.-W., Kim, B., Lim, S.-H., Lee, S.-H., Kim, K., Son, G., Jeon, H.-A., Moon, C., Sakong, J., & Choi, J.-W. (2021). Refined prefrontal working memory network as a neuromarker for Alzheimer's disease. *Biomedical Optics Express*, *12*(11), 7199. https://doi.org/10.1364/BOE.438926

Kim, H. (2021). Cerebral hemodynamics predicts the cortical area and coding scheme in the human brain for force generation by wrist muscles. *Behavioural Brain Research*, *396*, 112865. https://doi.org/10.1016/j.bbr.2020.112865

Kir, Y., Sayar-Akaslan, D., Agtas-Ertan, E., Kusman, A., Baskak, N., Baran, Z., Munir, K., & Baskak, B. (2021). Cortical activity during social acceptance and rejection task in social anxiety disorder: A controlled functional near infrared spectroscopy study. *Progress in Neuro-Psychopharmacology and Biological Psychiatry*, *104*, 110012. https://doi.org/10.1016/j.pnpbp.2020.110012

Kobayashi, M., Kanazawa, S., Yamaguchi, M. K., & O'Toole, A. J. (2021). Cortical processing of dynamic bodies in the superior occipito-temporal regions of the infants' brain: Difference from dynamic faces and inversion effect. *NeuroImage*, *244*, 118598. https://doi.org/10.1016/j.neuroimage.2021.118598

Kobayashi, S., Iwama, Y., Nishimaru, H., Matsumoto, J., Setogawa, T., Ono, T., & Nishijo, H. (2021). Examination of the prefrontal cortex hemodynamic responses to the fist-edge-palm task in naïve subjects using functional near-infrared spectroscopy. *Frontiers in Human Neuroscience*, *15*, 40. https://doi.org/10.3389/fnhum.2021.617626

König, N., Steber, S., Borowski, A., Bliem, H., & Rossi, S. (2021). Neural processing of cognitive control in an emotionally neutral context in anxiety patients. *Brain Sciences*, *11*(5), 543. https://doi.org/10.3390/brainsci11050543

Kotegawa, K., & Teramoto, W. (2022). Association of executive function capacity with gait motor imagery ability and PFC activity: An fNIRS study. *Neuroscience Letters*, *766*, 136350. https://doi.org/10.1016/j.neulet.2021.136350

Koyanagi, M., Yamada, M., Higashi, T., Mitsunaga, W., Moriuchi, T., & Tsujihata, M. (2021). The usefulness of functional near-infrared spectroscopy for the assessment of post-stroke depression. *Frontiers in Human Neuroscience*, *15*. https://doi.org/10.3389/fnhum.2021.680847

Krol, K. M., Namaky, N., Monakhov, M. V., Lai, P. S., Ebstein, R., & Grossmann, T. (2021). Genetic variation in the oxytocin system and its link to social motivation in human infants. *Psychoneuroendocrinology*, *131*, 105290. https://doi.org/10.1016/j.psyneuen.2021.105290

Kronovsek, T., Hermand, E., Berthoz, A., Castilla, A., Gallou-Guyot, M., Daviet, J.-C., & Perrochon, A. (2021). Age-related decline in visuo-spatial working memory is reflected by dorsolateral prefrontal activation and cognitive capabilities. *Behavioural Brain Research*, *398*, 112981. https://doi.org/10.1016/j.bbr.2020.112981

Kruppa, J. A., Reindl, V., Gerloff, C., Oberwelland Weiss, E., Prinz, J., Herpertz-Dahlmann, B., Konrad, K., & Schulte-Rüther, M. (2021). Brain and motor synchrony in children and adolescents with ASD—a fNIRS hyperscanning study. *Social Cognitive and Affective Neuroscience*, *16*(1–2), 103–116. https://doi.org/10.1093/scan/nsaa092

Kuai, S.-G., Liang, Q., He, Y.-Y., & Wu, H.-N. (2021). Higher anxiety rating does not mean poor speech performance: Dissociation of the neural mechanisms of anticipation and delivery of public speaking. *Brain Imaging and Behavior*, *15*(4), 1934–1943. https://doi.org/10.1007/s11682-020-00387-3

Kumar, V., Nichenmetla, S., Chhabra, H., Sreeraj, V. S., Rao, N. P., Kesavan, M., Varambally, S., Venkatasubramanian, G., & Gangadhar, B. N. (2021). Prefrontal cortex activation during working memory task in schizophrenia: A fNIRS study. *Asian Journal of Psychiatry*, *56*, 102507. https://doi.org/10.1016/j.ajp.2020.102507

Lamberti, N., Manfredini, F., Baroni, A., Crepaldi, A., Lavezzi, S., Basaglia, N., & Straudi, S. (2021). Motor cortical activation assessment in progressive multiple sclerosis patients enrolled in gait rehabilitation: A secondary analysis of the RAGTIME trial assisted by functional near-infrared spectroscopy. *Diagnostics*, *11*(6), 1068. https://doi.org/10.3390/diagnostics11061068

Lang, X., Wen, D., Li, Q., Yin, Q., Wang, M., & Xu, Y. (2021). FNIRS evaluation of frontal and temporal cortex activation by verbal fluency task and high-level cognition task for

detecting anxiety and depression. *Frontiers in Psychiatry*, *12*, 973. https://doi.org/10.3389/fpsyt.2021.690121

Laurent, S., Paire-Ficout, L., Boucheix, J.-M., Argon, S., & Hidalgo-Muñoz, A. (2021). Cortical activity linked to clocking in deaf adults: FNIRS insights with static and animated stimuli presentation. *Brain Sciences*, *11*(2), 196. https://doi.org/10.3390/brainsci11020196

Lawrence, R. J., Wiggins, I. M., Hodgson, J. C., & Hartley, D. E. H. (2021). Evaluating cortical responses to speech in children: A functional near-infrared spectroscopy (fNIRS) study. *Hearing Research*, *401*, 108155. https://doi.org/10.1016/j.heares.2020.108155

Lee, H., Choi, J., Jung, D., Hur, J.-W., & Cho, C.-H. (2021). The effects of virtual reality treatment on prefrontal cortex activity in patients with social anxiety disorder: Participatory and interactive virtual reality treatment study. *Journal of Medical Internet Research*, *23*(12), e31844. https://doi.org/10.2196/31844

Lei, Z., Bi, R., Mo, L., Yu, W., & Zhang, D. (2021). The brain mechanism of explicit and implicit processing of emotional prosodies: An fNIRS study. *Acta Psychologica Sinica*, *53*(1), 15–25. https://doi.org/10.3724/SP.J.1041.2021.00015

Li, E., Xiao, F., Zou, T., & Guo, J. (2021). Positive emotion of self-referential contexts could facilitate adult's novel word learning: An fNIRS study. *Brain and Language*, *221*, 104994. https://doi.org/10.1016/j.bandl.2021.104994

Li, H., Gong, A., Zhao, L., Zhang, W., Wang, F., & Fu, Y. (2021). Decoding of walking imagery and idle state using sparse representation based on fNIRS. *Computational Intelligence and Neuroscience*, 1–10. https://doi.org/10.1155/2021/6614112

Li, H., Wu, D., Yang, J., Luo, J., Xie, S., & Chang, C. (2021). Tablet use affects preschoolers' executive function: FNIRS evidence from the dimensional change card sort task. *Brain Sciences*, *11*(5), 567. https://doi.org/10.3390/brainsci11050567

Li, J., Mu, J., Shen, C., Yao, G., Feng, K., Zhang, X., & Liu, P. (2021). Abnormal cortical activation patterns among chinese-speaking schizophrenia patients during category and letter verbal fluency tasks revealed by multi-channel functional near-infrared spectroscopy. *Frontiers in Psychiatry*, *12*. https://doi.org/10.3389/fpsyt.2021.790732

Li, J., Yan, W.-J., Wu, Y., Tian, X.-X., & Zhang, Y.-W. (2022). Synaptosomal-associated protein 25 gene polymorphisms affect treatment efficiency of methylphenidate in children with attention-deficit hyperactivity disorder: An fNIRS study. *Frontiers in Behavioral Neuroscience*, *15*. https://doi.org/10.3389/fnbeh.2021.793643

Li, M., Yang, Y., Zhang, Y., Gao, Y., Jing, R., Dang, Y., Chen, X., He, J., & Si, J. (2021). Detecting residual awareness in patients with prolonged disorders of consciousness: An fNIRS study. *Frontiers in Neurology*, *12*. https://doi.org/10.3389/fneur.2021.618055

Li, R., Mayseless, N., Balters, S., & Reiss, A. L. (2021). Dynamic inter-brain synchrony in real-life inter-personal cooperation: A functional near-infrared spectroscopy hyperscanning study. *NeuroImage*, *238*, 118263. https://doi.org/10.1016/j.neuroimage.2021.118263

Li, W., Wang, F., Mayer, R. E., & Liu, T. (2021). Animated pedagogical agents enhance learning outcomes and brain activity during learning. *Journal of Computer Assisted Learning*, *38*(3), 621–637. https://doi.org/10.1111/jcal.12634

Li, X., Lipschutz, R., Hernandez, S. M., Biekman, B., Shen, S., Montgomery, D. A., Perlman, S. B., Pollonini, L., & Bick, J. (2021). Links between socioeconomic disadvantage, neural

function, and working memory in early childhood. *Developmental Psychobiology*, *63*(6), e22181. https://doi.org/10.1002/dev.22181

Li, Y., Chen, M., Zhang, R., & Li, X. (2021). Experiencing happiness together facilitates dyadic coordination through the enhanced interpersonal neural synchronization. *Social Cognitive and Affective Neuroscience*. https://doi.org/10.1093/scan/nsab114

Li, Y., Chen, R., Turel, O., Feng, T., Zhu, C.-Z., & He, Q. (2021). Dyad sex composition effect on inter-brain synchronization in face-to-face cooperation. *Brain Imaging and Behavior*, *15*(3), 1667–1675. https://doi.org/10.1007/s11682-020-00361-z

Li, Z., Li, J., Hong, B., Nolte, G., Engel, A. K., & Zhang, D. (2021). Speaker–listener neural coupling reveals an adaptive mechanism for speech comprehension in a noisy environment. *Cerebral Cortex*, *31*(10), 4719–4729. https://doi.org/10.1093/cercor/bhab118

Liang, Y., Fu, G., Yu, R., Bi, Y., & Ding, X. P. (2021). The role of reward system in dishonest behavior: A functional near-infrared spectroscopy study. *Brain Topography*, *34*(1), 64–77. https://doi.org/10.1007/s10548-020-00804-2

Liao, Y.-Y., Chen, I.-H., Hsu, W.-C., Tseng, H.-Y., & Wang, R.-Y. (2021). Effect of exergaming versus combined exercise on cognitive function and brain activation in frail older adults: A randomised controlled trial. *Annals of Physical and Rehabilitation Medicine*, *64*(5), 101492. https://doi.org/10.1016/j.rehab.2021.101492

Lin, X. A., Wang, C., Zhou, J., Sai, L., & Fu, G. (2021). Neural correlates of spontaneous deception in a non-competitive interpersonal scenario: A functional near-infrared spectroscopy (fNIRS) study. *Brain and Cognition*, *150*, 105704. https://doi.org/10.1016/j.bandc.2021.105704

Liu, L., Geng, Y., Cui, Y., Zhou, Y., Sun, G., Peng, C., Zhang, R., Ma, Y., Liu, Y., Sun, C., Hou, X., & Chen, J. (2021). Significance of the ability to differentiate emotional prosodies for the early diagnosis and prognostic prediction of mild hypoxic-ischemic encephalopathy in neonates. *International Journal of Developmental Neuroscience*, *81*(1), 51–59. https://doi.org/10.1002/jdn.10074

Liu, R., Reimer, B., Song, S., Mehler, B., & Solovey, E. (2021). Unsupervised fNIRS feature extraction with CAE and ESN autoencoder for driver cognitive load classification. *Journal of Neural Engineering*, *18*(3), 036002. https://doi.org/10.1088/1741-2552/abd2ca

Liu, T., Duan, L., Dai, R., Pelowski, M., & Zhu, C. (2021). Team-work, Team-brain: Exploring synchrony and team interdependence in a nine-person drumming task via multiparticipant hyperscanning and inter-brain network topology with fNIRS. *NeuroImage*, *237*, 118147. https://doi.org/10.1016/j.neuroimage.2021.118147

Long, Y., Zheng, L., Zhao, H., Zhou, S., Zhai, Y., & Lu, C. (2021). Interpersonal neural synchronization during interpersonal touch underlies affiliative pair bonding between romantic couples. *Cerebral Cortex*, *31*(3), 1647–1659. https://doi.org/10.1093/cercor/bhaa316

Lou, X.-Q., Liu, X., Liu, C.-H., Lin, H.-J., Liu, H., & Ling, J. (2021). [Therapeutic effect of electric-balance stimulation with scalp acupuncture for motor aphasia after cerebral infarction]. *Chinese Acupuncture & Moxibustion*, *41*(11), 1211–1215. https://doi.org/10.13703/j.0255-2930.20210302-k0005

Lu, H., Gong, Y., Huang, P., Zhang, Y., Guo, Z., Zhu, X., & You, X. (2021). Effect of repeated anodal hd-tdcs on executive functions: Evidence from a pilot and single-blinded fNIRS

study. *Frontiers in Human Neuroscience*, *14*, 609. https://doi.org/10.3389/fnhum.2020.583730

Lu, K., Qiao, X., Yun, Q., & Hao, N. (2021). Educational diversity and group creativity: Evidence from fNIRS hyperscanning. *NeuroImage*, *243*, 118564. https://doi.org/10.1016/j.neuroimage.2021.118564

Lyu, B., Pham, T., Blaney, G., Haga, Z., Sassaroli, A., Fantini, S., & Aeron, S. (2021). Domain adaptation for robust workload level alignment between sessions and subjects using fNIRS. *Journal of Biomedical Optics*, *26*(2). https://doi.org/10.1117/1.JBO.26.2.022908

Ma, T., Wang, S., Xia, Y., Zhu, X., Evans, J., Sun, Y., & He, S. (2021). CNN-based classification of fNIRS signals in motor imagery BCI system. *Journal of Neural Engineering*, *18*(5), 056019. https://doi.org/10.1088/1741-2552/abf187

Maidan, I., Hacham, R., Galperin, I., Giladi, N., Holtzer, R., Hausdorff, J. M., & Mirelman, A. (2022). Neural variability in the prefrontal cortex as a reflection of neural flexibility and stability in patients with parkinson disease. *Neurology*, *98*(8), e839–e847. https://doi.org/10.1212/WNL.0000000000013217

Maier, M. J., Rosenbaum, D., Brüne, M., Fallgatter, A. J., & Ehlis, A. (2021). The impact of TMS-enhanced cognitive control on forgiveness processes. *Brain and Behavior, 11*(5), e02131. https://doi.org/10.1002/brb3.2131

Maier, M. J., Schiel, J. E., Rosenbaum, D., Hautzinger, M., Fallgatter, A. J., & Ehlis, A.-C. (2021). To regulate or not to regulate: Emotion regulation in participants with low and high impulsivity. *Frontiers in Behavioral Neuroscience*, *15*, 645052. https://doi.org/10.3389/fnbeh.2021.645052

Manci, E., Deniz, O. C., Guducu, C., Gunay, E., & Bediz, C. S. (2021). Hemodynamic changes in athletes' brains: Is there any adaptation? *General Physiology and Biophysics*, *40*(05), 387–396. https://doi.org/10.4149/gpb_2021027

Mao, D., Wunderlich, J., Savkovic, B., Jeffreys, E., Nicholls, N., Lee, O. W., Eager, M., & McKay, C. M. (2021). Speech token detection and discrimination in individual infants using functional near-infrared spectroscopy. *Scientific Reports*, *11*(1), 24006. https://doi.org/10.1038/s41598-021-03595-z

Marks, R. A., Eggleston, R. L., Sun, X., Yu, C.-L., Zhang, K., Nickerson, N., Hu, X.-S., & Kovelman, I. (2021). The neurocognitive basis of morphological processing in typical and impaired readers. *Annals of Dyslexia*, 1–23. https://doi.org/10.1007/s11881-021-00239-9

Maruya, K., Arai, T., & Fujita, H. (2021). Brain activity in the prefrontal cortex during cognitive tasks and dual tasks in community-dwelling elderly people with pre-frailty: A pilot study for early detection of cognitive decline. *Healthcare*, *9*(10), 1250. https://doi.org/10.3390/healthcare9101250

Matarasso, A. K., Rieke, J. D., White, K., Yusufali, M. M., & Daly, J. J. (2021). Combined real-time fMRI and real time fNIRS brain computer interface (BCI): Training of volitional wrist extension after stroke, a case series pilot study. *PLOS ONE*, *16*(5), e0250431. https://doi.org/10.1371/journal.pone.0250431

Mateus, V., Osório, A., Miguel, H. O., Cruz, S., & Sampaio, A. (2021). Maternal sensitivity and infant neural response to touch: An fNIRS study. *Social Cognitive and Affective Neuroscience*, *16*(12), 1256–1263. https://doi.org/10.1093/scan/nsab069

Max, S. M., Schroeder, P. A., Blechert, J., Giel, K. E., Ehlis, A.-C., & Plewnia, C. (2021). Mind the food: Behavioural characteristics and imaging signatures of the specific handling of food objects. *Brain Structure and Function*, *226*(4), 1169–1183. https://doi.org/10.1007/s00429-021-02232-9

Mayseless, N., & Reiss, A. L. (2021). The neurodevelopmental basis of humor appreciation: A fNIRS study of young children. *PLOS ONE*, *16*(12), e0259422. https://doi.org/10.1371/journal.pone.0259422

McKay, C. A., Shing, Y. L., Rafetseder, E., & Wijeakumar, S. (2021). Home assessment of visual working memory in pre-schoolers reveals associations between behaviour, brain activation and parent reports of life stress. *Developmental Science*, *24*(4). https://doi.org/10.1111/desc.13094

McKay, C., Wijeakumar, S., Rafetseder, E., & Shing, Y. L. (2021). Disentangling age and schooling effects on inhibitory control development: An fNIRS investigation. *Developmental Science*, e13205. https://doi.org/10.1111/desc.13205

Mehta, R. K., & Nuamah, J. (2021). Relationship between acute physical fatigue and cognitive function during orthostatic challenge in men and women: A neuroergonomics investigation. *Human Factors: The Journal of the Human Factors and Ergonomics Society*, *63*(8), 1437–1448. https://doi.org/10.1177/0018720820936794

Meidenbauer, K. L., Choe, K. W., Cardenas-Iniguez, C., Huppert, T. J., & Berman, M. G. (2021). Load-dependent relationships between frontal fNIRS activity and performance: A data-driven PLS approach. *NeuroImage*, *230*, 117795. https://doi.org/10.1016/j.neuroimage.2021.117795

Meng, X., & Moriguchi, Y. (2021). Neural basis for egalitarian sharing in five-to six-year-old children. *Neuropsychologia*, *154*, 107787. https://doi.org/10.1016/j.neuropsychologia.2021.107787

Midha, S., Maior, H. A., Wilson, M. L., & Sharples, S. (2021). Measuring mental workload variations in office work tasks using fNIRS. *International Journal of Human-Computer Studies*, *147*, 102580. https://doi.org/10.1016/j.ijhcs.2020.102580

Miguel, H. O., Condy, E. E., Nguyen, T., Zeytinoglu, S., Blick, E., Bress, K., Khaksari, K., Dashtestani, H., Millerhagen, J., Shahmohammadi, S., Fox, N. A., & Gandjbakhche, A. (2021). Cerebral hemodynamic response during a live action-observation and action-execution task: A fNIRS study. *PLOS ONE*, *16*(8), e0253788. https://doi.org/10.1371/journal.pone.0253788

Mihara, M., Fujimoto, H., Hattori, N., Otomune, H., Kajiyama, Y., Konaka, K., Watanabe, Y., Hiramatsu, Y., Sunada, Y., Miyai, I., & Mochizuki, H. (2021). Effect of neurofeedback facilitation on poststroke gait and balance recovery: A randomized controlled trial. *Neurology*, *96*(21), e2587–e2598. https://doi.org/10.1212/WNL.0000000000011989

Montefinese, M., Pinti, P., Ambrosini, E., Tachtsidis, I., & Vinson, D. (2021). Inferior parietal lobule is sensitive to different semantic similarity relations for concrete and abstract words. *Psychophysiology*, *58*(3), e13750. https://doi.org/10.1111/psyp.13750

Moriguchi, Y. (2022). Relationship between cool and hot executive function in young children: A near-infrared spectroscopy study. *Developmental Science*, *25*(2), e13165. https://doi.org/10.1111/desc.13165

Moslehi, A. H., & Davies, T. C. (2021). EEG Electrode Selection for a Two-Class Motor Imagery Task in a BCI Using fNIRS Prior Data. *IEEE*, 6627–6630. https://doi.org/10.1109/EMBC46164.2021.9630786

Mukli, P., Csipo, T., Lipecz, A., Stylianou, O., Racz, F. S., Owens, C. D., Perry, J. W., Tarantini, S., Sorond, F. A., Kellawan, J. M., Purebl, G., Yang, Y., Sonntag, W. E., Csiszar, A., Ungvari, Z. I., & Yabluchanskiy, A. (2021). Sleep deprivation alters task-related changes in functional connectivity of the frontal cortex: A near-infrared spectroscopy study. *Brain and Behavior*, *11*(8), e02135. https://doi.org/10.1002/brb3.2135

Mülazimoğlu, E., Çakır, M. P., & Acartürk, C. (2021). The role of visual features in text-based CAPTCHAs: An fNIRS study for usable security. *Computational Intelligence and Neuroscience*, *2021*, 1–24. https://doi.org/10.1155/2021/8842420

Muñoz-Caracuel, M., Muñoz, V., Ruíz-Martínez, F. J., Di Domenico, D., Brigadoi, S., & Gómez, C. M. (2021). Multivariate analysis of the systemic response to auditory stimulation: An integrative approach. *Experimental Physiology*, *106*(4), 1072–1098. https://doi.org/10.1113/EP089125

Mushtaq, F., Wiggins, I. M., Kitterick, P. T., Anderson, C. A., & Hartley, D. E. H. (2021). Investigating cortical responses to noise-vocoded speech in children with normal hearing using functional near-infrared spectroscopy (fNIRS). *Journal of the Association for Research in Otolaryngology*, *22*(6), 703–717. https://doi.org/10.1007/s10162-021-00817-z

Nagels-Coune, L., Riecke, L., Benitez-Andonegui, A., Klinkhammer, S., Goebel, R., De Weerd, P., Lührs, M., & Sorger, B. (2021). See, hear, or feel – to speak: A Versatile multiple-choice functional near-infrared spectroscopy-brain-computer interface feasible with

visual, auditory, or tactile instructions. *Frontiers in Human Neuroscience*, *15*. https://doi.org/10.3389/fnhum.2021.784522

Nakamura, S., Yomota, S., Ito, H., Akinaga, N., Hori, A., Chinomi, K., Suzuki, H., Uchida, K., & Asada, T. (2021). A novel cognitive function scale using functional near-infrared spectroscopy for evaluating cognitive dysfunction. *Journal of Alzheimer's Disease*, *81*(4), 1579–1588. https://doi.org/10.3233/JAD-210072

Nakamura, T., Sasayama, D., Hagiwara, T., Kito, H., & Washizuka, S. (2021). Reduced functional connectivity in the prefrontal cortex of elderly catatonia patients: A longitudinal study using functional near-infrared spectroscopy. *Neuroscience Research*, *170*, 322–329. https://doi.org/10.1016/j.neures.2020.10.004

Narita, N., Kamiya, K., Iwaki, S., Ishii, T., Endo, H., Shimosaka, M., Uchida, T., Kantake, I., & Shibutani, K. (2021). Activation of prefrontal cortex in process of oral and finger shape discrimination: FNIRS study. *Frontiers in Neuroscience*, *15*, 6. https://doi.org/10.3389/fnins.2021.588593

Nguyen, T., Condy, E. E., Park, S., Friedman, B. H., & Gandjbakhche, A. (2021). Comparison of functional connectivity in the prefrontal cortex during a simple and an emotional go/no-go task in female versus male groups: An fNIRS study. *Brain Sciences*, *11*(7), 909. https://doi.org/10.3390/brainsci11070909

Nguyen, T., Hoehl, S., & Vrtička, P. (2021). A guide to parent-child fNIRS hyperscanning data processing and analysis. *Sensors*, *21*(12), 4075. https://doi.org/10.3390/s21124075

Nguyen, T., Miguel, H. O., Condy, E. E., Park, S., & Gandjbakhche, A. (2021). Using functional connectivity to examine the correlation between mirror neuron network and autistic traits

in a typically developing sample: A fNIRS study. *Brain Sciences*, *11*(3), 397. https://doi.org/10.3390/brainsci11030397

Nguyen, T., Schleihauf, H., Kayhan, E., Matthes, D., Vrtička, P., & Hoehl, S. (2021). Neural synchrony in mother–child conversation: Exploring the role of conversation patterns. *Social Cognitive and Affective Neuroscience*, *16*(1–2), 93–102. https://doi.org/10.1093/scan/nsaa079

Nguyen, T., Schleihauf, H., Kungl, M., Kayhan, E., Hoehl, S., & Vrtička, P. (2021). Interpersonal neural synchrony during father–child problem solving: An fNIRS hyperscanning study. *Child Development*, *92*(4), e565–e580. https://doi.org/10.1111/cdev.13510

Ni, L., Zhao, M., Hu, Z., Yang, K., Zhao, X., Niu, H., & Lin, H. (2021). Neural mechanism of shentai tea polyphenols on cognitive improvements for individuals with subjective cognitive decline: A functional near-infrared spectroscopy study. *Journal of Alzheimer's Disease*, *82*(3), 1137–1145. https://doi.org/10.3233/JAD-210469

Nijmeijer, S. E., van Tol, M.-J., Aleman, A., & Keijzer, M. (2021). Foreign language learning as cognitive training to prevent old age disorders? Protocol of a randomized controlled trial of language training vs. musical training and social interaction in elderly with subjective cognitive decline. *Frontiers in Aging Neuroscience*, *13*, 195. https://doi.org/10.3389/fnagi.2021.550180

Nishiyori, R., Harris, M. K., Baur, K., & Meehan, S. K. (2021). Changes in cortical hemodynamics with the emergence of skilled motor ability in infants: An fNIRS study. *Brain Research*, *1772*, 147666. https://doi.org/10.1016/j.brainres.2021.147666

Ohtani, T., Matsuo, K., Sutoh, C., Oshima, F., Hirano, Y., Wakabayashi, A., & Shimizu, E. (2021). Reduced brain activation in response to social cognition tasks in autism spectrum disorder with and without depression. *Neuropsychiatric Disease and Treatment*, *17*, 3015–3024. https://doi.org/10.2147/NDT.S327608

Ohtani, T., Wakabayashi, A., Sutoh, C., Oshima, F., Hirano, Y., & Shimizu, E. (2021). Ventrolateral prefrontal hemodynamic responses in autism spectrum disorder with and without depression. *PLOS ONE*, *16*(8), e0256780. https://doi.org/10.1371/journal.pone.0256780

Oku, A. Y. A., & Sato, J. R. (2021). Predicting student performance using machine learning in fNIRS data. *Frontiers in Human Neuroscience*, *15*. https://doi.org/10.3389/fnhum.2021.622224

Olszewska-Guizzo, A., Mukoyama, A., Naganawa, S., Dan, I., Husain, S. F., Ho, C. S., & Ho, R. (2021). Hemodynamic response to three types of urban spaces before and after lockdown during the COVID-19 pandemic. *International Journal of Environmental Research and Public Health*, *18*(11), 6118. https://doi.org/10.3390/ijerph18116118

Ong, S. K., Husain, S. F., Wee, H. N., Ching, J., Kovalik, J.-P., Cheng, M. S., Schwarz, H., Tang, T. B., & Ho, C. S. (2021). Integration of the cortical haemodynamic response measured by functional near-infrared spectroscopy and amino acid analysis to aid in the diagnosis of major depressive disorder. *Diagnostics*, *11*(11), 1978. https://doi.org/10.3390/diagnostics11111978

Ono, Y., Zhang, X., Noah, J. A., Dravida, S., & Hirsch, J. (2022). Bidirectional connectivity between broca's area and wernicke's area during interactive verbal communication. *Brain Connectivity*, *12*(3), 210–222. https://doi.org/10.1089/brain.2020.0790

Orcioli-Silva, D., Islam, A., Baker, M. R., Gobbi, L. T. B., Rochester, L., & Pantall, A. (2021). Bi-anodal transcranial direct current stimulation combined with treadmill walking decreases motor cortical activity in young and older adults. *Frontiers in Aging Neuroscience*, *13*. https://doi.org/10.3389/fnagi.2021.739998

Orcioli-Silva, D., Vitório, R., Beretta, V. S., da Conceição, N. R., Nóbrega-Sousa, P., Oliveira, A. S., & Gobbi, L. T. B. (2021). Is cortical activation during walking different between parkinson's disease motor subtypes? *The Journals of Gerontology: Series A*, *76*(4), 561–567. https://doi.org/10.1093/gerona/glaa174

Orcioli-Silva, D., Vitório, R., Nóbrega-Sousa, P., Beretta, V. S., Conceição, N. R. da, Oliveira, A. S., Pereira, M. P., & Gobbi, L. T. B. (2021). Cortical activity underlying gait improvements achieved with dopaminergic medication during usual walking and obstacle avoidance in parkinson disease. *Neurorehabilitation and Neural Repair*, *35*(5), 406–418. https://doi.org/10.1177/15459683211000736

Ortega, P., & Faisal, A. A. (2021). Deep learning multimodal fNIRS and EEG signals for bimanual grip force decoding. *Journal of Neural Engineering*, *18*(4), 0460e6. https://doi.org/10.1088/1741-2552/ac1ab3

Öztürk, Ö., Algun, Z. C., Bombacı, H., & Erdoğan, S. B. (2021). Changes in prefrontal cortex activation with exercise in knee osteoarthritis patients with chronic pain: An fNIRS study. *Journal of Clinical Neuroscience*, *90*, 144–151. https://doi.org/10.1016/j.jocn.2021.05.055

Pakray, H., Seng, E., Izzetoglu, M., & Holtzer, R. (2021). The effects of perceived pain in the past month on prefrontal cortex activation patterns assessed during cognitive and motor

performances in older adults. *Pain Medicine*, *22*(2), 303–314. https://doi.org/10.1093/pm/pnaa404

Pan, Y., Guyon, C., Borragán, G., Hu, Y., & Peigneux, P. (2021). Interpersonal brain synchronization with instructor compensates for learner's sleep deprivation in interactive learning. *Biochemical Pharmacology*, *191*, 114111. https://doi.org/10.1016/j.bcp.2020.114111

Panico, F., De Marco, S., Sagliano, L., D'Olimpio, F., Grossi, D., & Trojano, L. (2021). Brain hemodynamic response in Examiner–Examinee dyads during spatial short-term memory task: An fNIRS study. *Experimental Brain Research*, *239*(5), 1607–1616. https://doi.org/10.1007/s00221-021-06073-0

Papasideris, M., Ayaz, H., & Hall, P. A. (2021). Medial prefrontal brain activity correlates with emerging symptoms of anxiety and depression in late adolescence: A fNIRS study. *Developmental Psychobiology*, *63*(7), e22199. https://doi.org/10.1002/dev.22199

Papasideris, M., Ayaz, H., Safati, A. B., Morita, P. P., & Hall, P. A. (2021). Examining the relationships among adolescent health behaviours, prefrontal function, and academic achievement using fNIRS. *Developmental Cognitive Neuroscience*, *50*, 100983. https://doi.org/10.1016/j.dcn.2021.100983

Park, S., Reinl, M., & Schott, N. (2021). Effects of acute exercise at different intensities on fine motor-cognitive dual-task performance while walking: A functional near-infrared spectroscopy study. *European Journal of Neuroscience*, *54*(12), 8225–8248. https://doi.org/10.1111/ejn.15241

Pecukonis, M., Perdue, K. L., Wong, J., Tager-Flusberg, H., & Nelson, C. A. (2021). Exploring the relation between brain response to speech at 6-months and language outcomes at 24-

months in infants at high and low risk for autism spectrum disorder: A preliminary functional near-infrared spectroscopy study. *Developmental Cognitive Neuroscience*, *47*, 100897. https://doi.org/10.1016/j.dcn.2020.100897

Pelicioni, P. H. S., Lord, S. R., Okubo, Y., & Menant, J. C. (2022). Cortical activation during gait adaptability in people with Parkinson's disease. *Gait & Posture*, *91*, 247–253. https://doi.org/10.1016/j.gaitpost.2021.10.038

Pelicioni, P. H. S., Lord, S. R., Sturnieks, D. L., Halmy, B., & Menant, J. C. (2021). Cognitive and motor cortical activity during cognitively demanding stepping tasks in older people at low and high risk of falling. *Frontiers in Medicine*, *8*, 1016. https://doi.org/10.3389/fmed.2021.554231

Perpetuini, D., Cardone, D., Filippini, C., Chiarelli, A. M., & Merla, A. (2021). A motion artifact correction procedure for fNIRS signals based on wavelet transform and infrared thermography video tracking. *Sensors*, *21*(15), 5117. https://doi.org/10.3390/s21155117

Petersen, J., Ong, C. W., Hancock, A. S., Gillam, R. B., Levin, M. E., & Twohig, M. P. (2021). An examination of the relationship between perfectionism and neurological functioning. *Journal of Cognitive Psychotherapy*, *35*(3), 195–211. https://doi.org/10.1891/JCPSY-D-20-00037

Piau, C., Mahmoudzadeh, M., Kibleur, A., Polosan, M., David, O., & Wallois, F. (2021). Cortical hemodynamic mechanisms of reversal learning using high-resolution functional near-infrared spectroscopy: A pilot study. *Neurophysiologie Clinique*, *51*(5), 409–424. https://doi.org/10.1016/j.neucli.2021.08.001

Piazza, E. A., Cohen, A., Trach, J., & Lew-Williams, C. (2021). Neural synchrony predicts children's learning of novel words. *Cognition*, *214*, 104752. https://doi.org/10.1016/j.cognition.2021.104752

Pinti, P., Devoto, A., Greenhalgh, I., Tachtsidis, I., Burgess, P. W., & de C Hamilton, A. F. (2021). The role of anterior prefrontal cortex (area 10) in face-to-face deception measured with fNIRS. *Social Cognitive and Affective Neuroscience*, *16*(1–2), 129–142. https://doi.org/10.1093/scan/nsaa086

Prôa, R., Balardin, J., de Faria, D. D., Paulo, A. M., Sato, J. R., Baltazar, C. A., Borges, V., Azevedo Silva, S. M. C., Ferraz, H. B., & de Carvalho Aguiar, P. (2021). Motor cortex activation during writing in focal upper-limb dystonia: An fNIRS study. *Neurorehabilitation and Neural Repair*, *35*(8), 729–737. https://doi.org/10.1177/15459683211019341

Qi, L., Yin, Y., Bu, L., Tang, Z., Tang, L., & Dong, G. (2021). Acute VR competitive cycling exercise enhanced cortical activations and brain functional network efficiency in MA-dependent individuals. *Neuroscience Letters*, *757*, 135969. https://doi.org/10.1016/j.neulet.2021.135969

Qing, K., Huang, R., & Hong, K.-S. (2021). Decoding three different preference levels of consumers using convolutional neural network: A functional near-infrared spectroscopy study. *Frontiers in Human Neuroscience*, *14,* 583. https://doi.org/10.3389/fnhum.2020.597864

Quiñones-Camacho, L. E., Fishburn, F. A., Belardi, K., Williams, D. L., Huppert, T. J., & Perlman, S. B. (2021). Dysfunction in interpersonal neural synchronization as a

mechanism for social impairment in autism spectrum disorder. *Autism Research*, *14*(8), 1585–1596. https://doi.org/10.1002/aur.2513

Quiñones-Camacho, L. E., Hoyniak, C. P., Wakschlag, L. S., & Perlman, S. B. (2021). Getting in synch: Unpacking the role of parent–child synchrony in the development of internalizing and externalizing behaviors. *Development and Psychopathology*, 1–13. https://doi.org/10.1017/S0954579421000468

Rahman, T. T., Polskaia, N., St-Amant, G., Salzman, T., Vallejo, D. T., Lajoie, Y., & Fraser, S. A. (2021). An fNIRS investigation of discrete and continuous cognitive demands during dual-task walking in young adults. *Frontiers in Human Neuroscience*, *15*. https://doi.org/10.3389/fnhum.2021.711054

Re, R., Messenio, D., Marano, G., Spinelli, L., Pirovano, I., Contini, D., Colombo, R., Boracchi, P., Biganzoli, E., Cubeddu, R., & Torricelli, A. (2021). Monitoring the haemodynamic response to visual stimulation in glaucoma patients. *Scientific Reports*, *11*(1), 1–11. https://doi.org/10.1038/s41598-021-92857-x

Reddy, P., Izzetoglu, M., Shewokis, P. A., Sangobowale, M., Diaz-Arrastia, R., & Izzetoglu, K. (2021). Evaluation of fNIRS signal components elicited by cognitive and hypercapnic stimuli. *Scientific Reports*, *11*(1), 1–15. https://doi.org/10.1038/s41598-021-02076-7

Ren, H., Zou, L., Wang, L., Lu, C., Yuan, Y., Dai, C., & Chen, W. (2021). Evaluation of the short-term music therapy on brain functions of preterm infants using functional near-infrared spectroscopy. *Frontiers in Neurology*, *12*, 1634. https://doi.org/10.3389/fneur.2021.649340

Richardson, H., Taylor, J., Kane-Grade, F., Powell, L., Bosquet Enlow, M., & Nelson, C. A. (2021). Preferential responses to faces in superior temporal and medial prefrontal cortex

in three-year-old children. *Developmental Cognitive Neuroscience*, *50*, 100984. https://doi.org/10.1016/j.dcn.2021.100984

Richter, M., Vignotto, M., Mock, J., Obrig, H., & Rossi, S. (2021). Different word-learning contexts alter phonotactic rule learning in 6-month-olds. *Language, Cognition and Neuroscience*, *36*(9), 1135–1158. https://doi.org/10.1080/23273798.2021.1921815

Rocco, G., Lebrun, J., Meste, O., & Magnie-Mauro, M.-N. (2021). A chiral fNIRS Spotlight on cerebellar activation in a finger tapping task. *IEEE*, 1018–1021. https://doi.org/10.1109/EMBC46164.2021.9629565

Rosenbaum, D., Int-Veen, I., Laicher, H., Torka, F., Kroczek, A., Rubel, J., Lawyer, G., Bürger, Z., Bihlmaier, I., Storchak, H., Velten-Schurian, K., Dresler, T., Täglich, R., Schopp, B., Nürk, H.-C., Derntl, B., Nieratschker, V., Fallgatter, A. J., & Ehlis, A.-C. (2021). Insights from a laboratory and naturalistic investigation on stress, rumination and frontal brain functioning in MDD: An fNIRS study. *Neurobiology of Stress*, *15*, 100344. https://doi.org/10.1016/j.ynstr.2021.100344

Ross, D., Wagshul, M. E., Izzetoglu, M., & Holtzer, R. (2021). Prefrontal cortex activation during dual-task walking in older adults is moderated by thickness of several cortical regions. *GeroScience*, *43*(4), 1959–1974. https://doi.org/10.1007/s11357-021-00379-1

Rotgans, J. I. (2021). Learning to diagnose X-rays: A neuroscientific study of practice-related activation changes in the prefrontal cortex. *Diagnosis*. https://doi.org/10.1515/dx-2021-0104

Rovetti, J., Goy, H., Nurgitz, R., & Russo, F. A. (2021). Comparing verbal working memory load in auditory and visual modalities using functional near-infrared spectroscopy. *Behavioural Brain Research*, *402*, 113102. https://doi.org/10.1016/j.bbr.2020.113102

Rybář, M., Poli, R., & Daly, I. (2021). Decoding of semantic categories of imagined concepts of animals and tools in fNIRS. *Journal of Neural Engineering*, *18*(4), 046035. https://doi.org/10.1088/1741-2552/abf2e5

Saikia, M. J., Besio, W. G., & Mankodiya, K. (2021). The Validation of a portable functional NIRS system for assessing mental workload. *Sensors*, *21*(11), 3810. https://doi.org/10.3390/s21113810

Sakai, K., Tanabe, J., Goto, K., Kumai, K., & Ikeda, Y. (2022). Comparison of functional connectivity during visual-motor illusion, observation, and motor execution. *Journal of Motor Behavior*, *54*(3), 354–362. https://doi.org/10.1080/00222895.2021.1976717

Sakuma, S., Inamoto, K., Yamaguchi, Y., Takagi, S., & Higuchi, N. (2021). Changes in prefrontal cerebral hemodynamics during intermittent pain stimulation to gingiva: Preliminary study using functional near infrared spectroscopy. *Journal of Dental Sciences*, *16*(3), 980–986. https://doi.org/10.1016/j.jds.2020.09.013

Salzman, T., Aboualmagd, A., Badawi, H., Tobón-Vallejo, D., Kim, H., Dahroug, L., Laamarti, F., El Saddik, A., & Fraser, S. (2021). Prefrontal cortex involvement during dual-task stair climbing in healthy older adults: An fNIRS study. *Brain Sciences*, *11*(1), 71. https://doi.org/10.3390/brainsci11010071

Salzman, T., Tobón Vallejo, D., Polskaia, N., Michaud, L., St-Amant, G., Lajoie, Y., & Fraser, S. (2021). Hemodynamic and behavioral changes in older adults during cognitively demanding dual tasks. *Brain and Behavior*, *11*(3), e02021. https://doi.org/10.1002/brb3.2021

Sato, J. R., Junior, C. E. B., de Araújo, E. L. M., de Souza Rodrigues, J., & Andrade, S. M. (2021). A guide for the use of fNIRS in microcephaly associated to congenital Zika virus infection. *Scientific Reports*, *11*(1), 1–13. https://doi.org/10.1038/s41598-021-97450-w

Sawamura, D., Sakuraba, S., Yoshida, K., Hasegawa, N., Suzuki, Y., Yoshida, S., Honke, T., & Sakai, S. (2021). Chopstick operation training with the left non-dominant hand. *Translational Neuroscience*, *12*(1), 385–395. https://doi.org/10.1515/tnsci-2020-0189

Sayar-Akaslan, D., Baskak, B., Kir, Y., Kusman, A., Yalcinkaya, B., Çakmak, I. B., & Munir, K. (2021). Cortical activity measured by functional near infrared spectroscopy during a theory of mind task in subjects with schizophrenia, bipolar disorder and healthy controls. *Journal of Affective Disorders*, *282*, 329–339. https://doi.org/10.1016/j.jad.2020.12.094

Schommartz, I., Dix, A., Passow, S., & Li, S.-C. (2021). Functional effects of bilateral dorsolateral prefrontal cortex modulation during sequential decision-making: A functional near-infrared spectroscopy study with offline transcranial direct current stimulation. *Frontiers in Human Neuroscience*, *14*, 619. https://doi.org/10.3389/fnhum.2020.605190

Schumacher, F. K., Schumacher, L. V., Amtage, F., Horn, A., Egger, K., Piroth, T., Weiller, C., Schelter, B. O., Coenen, V. A., & Kaller, C. P. (2021). The rostro-caudal gradient in the prefrontal cortex and its modulation by subthalamic deep brain stimulation in Parkinson's disease. *Scientific Reports*, *11*(1), 1–13. https://doi.org/10.1038/s41598-021-81535-7

Segar, R., Chhabra, H., Sreeraj, V. S., Parlikar, R., Kumar, V., Ganesan, V., & Kesavan, M. (2021). FNIRS study of prefrontal activation during emotion recognition–A Potential endophenotype for bipolar I disorder? *Journal of Affective Disorders*, *282*, 869–875. https://doi.org/10.1016/j.jad.2020.12.153

Shader, M. J., Luke, R., Gouailhardou, N., & McKay, C. M. (2021). The use of broad vs restricted regions of interest in functional near-infrared spectroscopy for measuring cortical activation to auditory-only and visual-only speech. *Hearing Research*, *406*, 108256. https://doi.org/10.1016/j.heares.2021.108256

Shenoy, S., Khandekar, P., & Sathe, A. (2021). Young and middle-aged adults differ in neural correlate of sustained attention: A fNIRS study. *Current Aging Science*, *14*(3), 201–213. https://doi.org/10.2174/1874609814666210716111022

Shi, P., Li, A., & Yu, H. (2021). Response of the cerebral cortex to resistance and non-resistance exercise under different trajectories: A functional near-infrared spectroscopy study. *Frontiers in Neuroscience*, *15*. https://doi.org/10.3389/fnins.2021.685920

Shinozuka, K., Niioka, K., Tokuda, T., Kyutoku, Y., Okuno, K., Takahashi, T., & Dan, I. (2021). Language familiarity and proficiency leads to differential cortical processing during translation between distantly related languages. *Frontiers in Human Neuroscience*, *15*, 26. https://doi.org/10.3389/fnhum.2021.593108

Si, X., Li, S., Xiang, S., Yu, J., & Ming, D. (2021). Imagined speech increases the hemodynamic response and functional connectivity of the dorsal motor cortex. *Journal of Neural Engineering*, *18*(5), 056048. https://doi.org/10.1088/1741-2552/ac25d9

Si, X., Xiang, S., Zhang, L., Li, S., Zhang, K., & Ming, D. (2021). Acupuncture with deqi modulates the hemodynamic response and functional connectivity of the prefrontal-motor cortical network. *Frontiers in Neuroscience*, *15*, 803. https://doi.org/10.3389/fnins.2021.693623

Skau, S., Jonsdottir, I. H., Sjörs Dahlman, A., Johansson, B., & Kuhn, H. G. (2021). Exhaustion disorder and altered brain activity in frontal cortex detected with fNIRS. *Stress*, *24*(1), 64–75. https://doi.org/10.1080/10253890.2020.1777972

St George, R. J., Hinder, M. R., Puri, R., Walker, E., & Callisaya, M. L. (2021). Functional near-infrared spectroscopy reveals the compensatory potential of pre-frontal cortical activity for standing balance in young and older adults. *Neuroscience*, *452*, 208–218. https://doi.org/10.1016/j.neuroscience.2020.10.027

Steber, S., & Rossi, S. (2021). The challenge of learning a new language in adulthood: Evidence from a multi-methodological neuroscientific approach. *PLOS ONE*, *16*(2), e0246421. https://doi.org/10.1371/journal.pone.0246421

Stojan, R., & Voelcker-Rehage, C. (2021). Neurophysiological correlates of age differences in driving behavior during concurrent subtask performance. *NeuroImage*, *225*, 117492. https://doi.org/10.1016/j.neuroimage.2020.117492

Storchak, H., Hudak, J., Dresler, T., Haeussinger, F. B., Fallgatter, A. J., & Ehlis, A.-C. (2021). Monitoring processes and their neuronal correlates as the basis of auditory verbal hallucinations in a non-clinical sample. *Frontiers in Psychiatry*, *12*, 1678. https://doi.org/10.3389/fpsyt.2021.644052

Struckmann, W., Persson, J., Gingnell, M., Weigl, W., Wass, C., & Bodén, R. (2021). Unchanged cognitive performance and concurrent prefrontal blood oxygenation after accelerated intermittent theta-burst stimulation in depression: A sham-controlled study. *Frontiers in Psychiatry*, *12*, 1061. https://doi.org/10.3389/fpsyt.2021.659571

Struckmann, W., Persson, J., Weigl, W., Gingnell, M., & Bodén, R. (2021). Modulation of the prefrontal blood oxygenation response to intermittent theta-burst stimulation in

depression: A sham-controlled study with functional near-infrared spectroscopy. *The World Journal of Biological Psychiatry*, *22*(4), 247–256. https://doi.org/10.1080/15622975.2020.1785007

Su, W.-C., Culotta, M., Tsuzuki, D., & Bhat, A. (2021). Movement kinematics and cortical activation in children with and without autism spectrum disorder during sway synchrony tasks: An fNIRS study. *Scientific Reports*, *11*(1), 1–13. https://doi.org/10.1038/s41598-021-94519-4

Sun, B., Xiao, W., Lin, S., Shao, Y., Li, W., & Zhang, W. (2021). Cooperation with partners of differing social experience: An fNIRS-based hyperscanning study. *Brain and Cognition*, *154*, 105803. https://doi.org/10.1016/j.bandc.2021.105803

Takahashi, R., Fujita, K., Kobayashi, Y., Ogawa, T., Teranishi, M., & Kawamura, M. (2021). Effect of muscle fatigue on brain activity in healthy individuals. *Brain Research*, *1764*, 147469. https://doi.org/10.1016/j.brainres.2021.147469

Talamonti, D., Vincent, T., Fraser, S., Nigam, A., Lesage, F., & Bherer, L. (2021). The benefits of physical activity in individuals with cardiovascular risk factors: A longitudinal investigation using fNIRS and dual-task walking. *Journal of Clinical Medicine*, *10*(4), 579. https://doi.org/10.3390/jcm10040579

Tan, H.-X., Wei, Q.-C., Chen, Y., Xie, Y.-J., Guo, Q.-F., He, L., & Gao, Q. (2021). The immediate effects of intermittent theta burst stimulation of the cerebellar vermis on cerebral cortical excitability during a balance task in healthy individuals: A pilot study. *Frontiers in Human Neuroscience*, *15*, 689. https://doi.org/10.3389/fnhum.2021.748241

Tang, T. B., Chong, J. S., Kiguchi, M., Funane, T., & Lu, C.-K. (2021). Detection of emotional sensitivity using fNIRS based dynamic functional connectivity. *IEEE Transactions on

Neural Systems and Rehabilitation Engineering, *29*, 894–904. https://doi.org/10.1109/TNSRE.2021.3078460

Teo, W., Rantalainen, T., Nuzum, N., Valente, L., & Macpherson, H. (2021). Altered prefrontal cortex responses in older adults with subjective memory complaints and dementia during dual-task gait: An fNIRS study. *European Journal of Neuroscience*, *53*(4), 1324–1333. https://doi.org/10.1111/ejn.14989

Tian, X., Liu, Y., Guo, Z., Cai, J., Tang, J., Chen, F., & Zhang, H. (2021). Cerebral representation of sound localization using functional near-infrared spectroscopy. *Frontiers in Neuroscience*, *15*, 1648. https://doi.org/10.3389/fnins.2021.739706

Tinga, A. M., Clim, M.-A., de Back, T. T., & Louwerse, M. M. (2021). Measures of prefrontal functional near-infrared spectroscopy in visuomotor learning. *Experimental Brain Research*, *239*(4), 1061–1072. https://doi.org/10.1007/s00221-021-06039-2

Trambaiolli, L. R., Tossato, J., Cravo, A. M., Biazoli, C. E., & Sato, J. R. (2021). Subject-independent decoding of affective states using functional near-infrared spectroscopy. *PLOS ONE*, *16*(1), e0244840. https://doi.org/10.1371/journal.pone.0244840

Tung, H., Lin, W.-H., Lan, T.-H., Hsieh, P. F., Chiang, M.-C., Lin, Y.-Y., & Peng, S.-J. (2021). Network reorganization during verbal fluency task in fronto-temporal epilepsy: A functional near-infrared spectroscopy study. *Journal of Psychiatric Research*, *138*, 541–549. https://doi.org/10.1016/j.jpsychires.2021.05.012

Tyagi, O., Hopko, S., Kang, J., Shi, Y., Du, J., & Mehta, R. K. (2021). Modeling brain dynamics during virtual reality-based emergency response learning under stress. *Human Factors: The Journal of the Human Factors and Ergonomics Society*. https://doi.org/10.1177/00187208211054894

Veit, R., Schag, K., Schopf, E., Borutta, M., Kreutzer, J., Ehlis, A.-C., Zipfel, S., Giel, K. E., Preissl, H., & Kullmann, S. (2021). Diminished prefrontal cortex activation in patients with binge eating disorder associates with trait impulsivity and improves after impulsivity-focused treatment based on a randomized controlled IMPULS trial. *NeuroImage: Clinical*, *30*, 102679. https://doi.org/10.1016/j.nicl.2021.102679

Vitorio, R., Stuart, S., Giritharan, A., Quinn, J., Nutt, J. G., & Mancini, M. (2021). Changes in prefrontal cortical activity and turning in response to dopaminergic and cholinergic therapy in Parkinson's disease: A randomized cross-over trial. *Parkinsonism & Related Disorders*, *86*, 10–14. https://doi.org/10.1016/j.parkreldis.2021.03.014

Wagner, J. C., Zinos, A., Chen, W.-L., Conant, L., Malloy, M., Heffernan, J., Quirk, B., Sugar, J., Prost, R., Whelan, J. B., Beardsley, S. A., & Whelan, H. T. (2021). Comparison of whole-head functional near-infrared spectroscopy with functional magnetic resonance imaging and potential application in pediatric neurology. *Pediatric Neurology*, *122*, 68–75. https://doi.org/10.1016/j.pediatrneurol.2021.06.003

Wang, F., Jiang, Z., Li, X., Bu, L., & Ji, Y. (2021). Functional brain network analysis of knowledge transfer while engineering problem-solving. *Frontiers in Human Neuroscience*, *15*. https://doi.org/10.3389/fnhum.2021.713692

Wang, J., Grant, T., Velipasalar Gursoy, S., Geng, B., & Hirshfield, L. (2021). Taking a deeper look at the brain: Predicting visual perceptual and working memory load from high-density fNIRS data. *IEEE Journal of Biomedical and Health Informatics*. https://doi.org/10.1109/JBHI.2021.3133871

Wang, J., Sakata, C., & Moriguchi, Y. (2021). The neurobehavioral relationship between executive function and creativity during early childhood. *Developmental Psychobiology*, *63*(7), e22191. https://doi.org/10.1002/dev.22191

Wang, R., Hao, Y., Yu, Q., Chen, M., Humar, I., & Fortino, G. (2021). Depression analysis and recognition based on functional near-infrared spectroscopy. *IEEE Journal of Biomedical and Health Informatics*, *25*(12), 4289–4299. https://doi.org/10.1109/JBHI.2021.3076762

Wang, Y., Liu, L., Zhang, Y., Wei, C., Xin, T., He, Q., Hou, X., & Liu, Y. (2021). The neural processing of vocal emotion after hearing reconstruction in prelingual deaf children: A Functional near-infrared spectroscopy brain imaging study. *Frontiers in Neuroscience*, *15*, 945. https://doi.org/10.3389/fnins.2021.705741

Wanniarachchi, H., Lang, Y., Wang, X., Pruitt, T., Nerur, S., Chen, K.-Y., & Liu, H. (2021). Alterations of cerebral hemodynamics and network properties induced by newsvendor problem in the human prefrontal cortex. *Frontiers in Human Neuroscience*, *14*, 598. https://doi.org/10.3389/fnhum.2020.598502

Wei, Y., Chen, Q., Curtin, A., Tu, L., Tang, X., Tang, Y., Xu, L., Qian, Z., Zhou, J., Zhu, C., Zhang, T., & Wang, J. (2021). Functional near-infrared spectroscopy (fNIRS) as a tool to assist the diagnosis of major psychiatric disorders in a Chinese population. *European Archives of Psychiatry and Clinical Neuroscience*, *271*(4), 745–757. https://doi.org/10.1007/s00406-020-01125-y

Weibley, H., Di Filippo, M., Liu, X., Lazenby, L., Goscha, J., Ferreira, A., Muscalu, L., & Rader, N. (2021). FNIRS monitoring of infant prefrontal cortex during crawling and an executive functioning task. *Frontiers in Behavioral Neuroscience*, *15*, 180. https://doi.org/10.3389/fnbeh.2021.675366

Wen, D., Lang, X., Zhang, H., Li, Q., Yin, Q., Chen, Y., & Xu, Y. (2021). Task and non-task brain activation differences for assessment of depression and anxiety by fNIRS. *Frontiers in Psychiatry*, *12*. https://doi.org/10.3389/fpsyt.2021.758092

White, B. E., & Langdon, C. (2021). The cortical organization of listening effort: New insight from functional near-infrared spectroscopy. *NeuroImage*, *240*, 118324. https://doi.org/10.1016/j.neuroimage.2021.118324

Wickramaratne, S. D., & Mahmud, Md. S. (2021). Conditional-GAN based data augmentation for deep learning task classifier improvement using fNIRS data. *Frontiers in Big Data*, *4*. https://doi.org/10.3389/fdata.2021.659146

Wu, S., Cai, S., Xiong, G., Dong, Z., Guo, H., Han, J., & Ye, T. (2021). The only-child effect in the neural and behavioral signatures of trust revealed by fNIRS hyperscanning. *Brain and Cognition*, *149*, 105692. https://doi.org/10.1016/j.bandc.2021.105692

Xiang, Y., Li, Y., Shu, C., Liu, Z., Wang, H., & Wang, G. (2021). Prefrontal cortex activation during verbal fluency task and tower of london task in schizophrenia and major depressive disorder. *Frontiers in Psychiatry*, *12*, 1638. https://doi.org/10.3389/fpsyt.2021.709875

Xu, L., Sun, Z., Xie, J., Yu, J., Li, J., & Wang, J. (2021). Identification of autism spectrum disorder based on short-term spontaneous hemodynamic fluctuations using deep learning in a multi-layer neural network. *Clinical Neurophysiology*, *132*(2), 457–468. https://doi.org/10.1016/j.clinph.2020.11.037

Yaghmour, A., Rafiul Amin, Md., & Faghih, R. T. (2021). Decoding a music-modulated cognitive arousal state using electrodermal activity and functional near-infrared

spectroscopy measurements. *IEEE*, 1055–1060. https://doi.org/10.1109/EMBC46164.2021.9630879

Yamashita, R., Chen, C., Matsubara, T., Hagiwara, K., Inamura, M., Aga, K., Hirotsu, M., Seki, T., Takao, A., Nakagawa, E., Kobayashi, A., Fujii, Y., Hirata, K., Ikei, H., Miyazaki, Y., & Nakagawa, S. (2021). The mood-improving effect of viewing images of nature and its neural substrate. *International Journal of Environmental Research and Public Health*, *18*(10), 5500. https://doi.org/10.3390/ijerph18105500

Yan, W., Ji, W., Su, C., Yu, Y., Yu, X., & Chen, L. (2021). Anger experience and anger expression through drawing in schizophrenia: An fNIRS study. *Frontiers in Psychology*, *12*, 721148. https://doi.org/10.3389/fpsyg.2021.721148

Yang, Q., Song, X., Dong, M., Li, J., & Proctor, R. W. (2021). The Underlying neural mechanisms of interpersonal situations on collaborative ability: A hyperscanning study using functional near-infrared spectroscopy. *Social Neuroscience*, *16*(5), 549–563. https://doi.org/10.1080/17470919.2021.1965017

Yang, X., Liu, X., Zeng, Y., Wu, R., Zhao, W., Xin, F., Yao, S., Kendrick, K. M., Ebstein, R. P., & Becker, B. (2021). Secondary rewards acquire enhanced incentive motivation via increasing anticipatory activity of the lateral orbitofrontal cortex. *Brain Structure and Function*, *226*(7), 2339–2355. https://doi.org/10.1007/s00429-021-02333-5

Yang, Y., Li, Y., Wang, X., Liu, N., Jiang, K., Zhang, S., & Qiu, J. (2021). Cognitive inhibition mediates the relationship between ESL listening proficiency and English spoken word segmentation in Chinese learners: A functional near-infrared spectroscopy (fNIRS) study. *Journal of Neurolinguistics*, *59*, 100987. https://doi.org/10.1016/j.jneuroling.2021.100987

Yeung, M. K., Lee, T. L., & Chan, A. S. (2021a). Negative mood is associated with decreased prefrontal cortex functioning during working memory in young adults. *Psychophysiology*, *58*(6), e13802. https://doi.org/10.1111/psyp.13802

Yeung, M. K., Lee, T. L., & Chan, A. S. (2021b). Depressive and anxiety symptoms are related to decreased lateral prefrontal cortex functioning during cognitive control in older people. *Biological Psychology*, *166*, 108224. https://doi.org/10.1016/j.biopsycho.2021.108224

Yeung, M. K., Lee, T. L., Han, Y. M. Y., & Chan, A. S. (2021). Prefrontal activation and pupil dilation during n-back task performance: A combined fNIRS and pupillometry study. *Neuropsychologia*, *159*, 107954. https://doi.org/10.1016/j.neuropsychologia.2021.107954

Yoo, S.-H., Santosa, H., Kim, C.-S., & Hong, K.-S. (2021). Decoding multiple sound-categories in the auditory cortex by neural networks: An fNIRS study. *Frontiers in Human Neuroscience*, *15*, 211. https://doi.org/10.3389/fnhum.2021.636191

Yu, L., Long, Q., Tang, Y., Yin, S., Chen, Z., Zhu, C., & Chen, A. (2021). Improving emotion regulation through real-time neurofeedback training on the right dorsolateral prefrontal cortex: Evidence from behavioral and brain network analyses. *Frontiers in Human Neuroscience*, *15*, 135. https://doi.org/10.3389/fnhum.2021.620342

Yu, M., & Liu, Y. (2021). Differences in executive function of the attention network between athletes from interceptive and strategic sports. *Journal of Motor Behavior*, *53*(4), 419–430. https://doi.org/10.1080/00222895.2020.1790486

Yu, N., Liang, S., Lu, J., Shu, Z., Li, H., Yu, Y., Wu, J., & Han, J. (2021). Quantified assessment of deep brain stimulation on Parkinson’s patients with task fNIRS measurements and functional connectivity analysis: A pilot study. *Chinese Neurosurgical Journal*, *7*(1), 171–181. https://doi.org/10.1186/s41016-021-00251-3

Yu, Q., Cheval, B., Becker, B., Herold, F., Chan, C. C. H., Delevoye-Turrell, Y. N., Guérin, S. M. R., Loprinzi, P., Mueller, N., & Zou, L. (2021). Episodic memory encoding and retrieval in face-name paired paradigm: An fNIRS study. *Brain Sciences*, *11*(7), 951. https://doi.org/10.3390/brainsci11070951

Yuan, D., Zhang, R., Liu, J., Feng, D., Hu, Y., Li, X., Wang, Y., & Zhou, X. (2022). Interpersonal neural synchronization could predict the outcome of mate choice. *Neuropsychologia*, *165*, 108112. https://doi.org/10.1016/j.neuropsychologia.2021.108112

Yuan, I., Nelson, O., Barr, G. A., Zhang, B., Topjian, A. A., DiMaggio, T. J., Lang, S., Christ, L. A., Izzetoglu, K., Greco, C. C., Kurth, C. D., & Ganesh, A. (2022). Functional near-infrared spectroscopy to assess pain in neonatal circumcisions. *Pediatric Anesthesia*, *32*(3), 404–412. https://doi.org/10.1111/pan.14326

Yuan, J., Zheng, Z., Cao, Y., Chen, J., Li, Y., & Lei, Y. (2021). Low-frequency magnetic stimulation of shenmen acupoint reduces blood oxygen levels in the prefrontal cortex of healthy subjects: A near-infrared brain functional imaging study. *Chinese Journal of Integrative Medicine*, *27*(8), 585–588. https://doi.org/10.1007/s11655-021-3291-z

Zhai, T., Ash-Rafzadeh, A., Hu, X., Kim, J., San Juan, J. D., Filipiak, C., Guo, K., Islam, M. N., Kovelman, I., & Basura, G. J. (2021). Tinnitus and auditory cortex; Using adapted functional near-infrared spectroscopy to expand brain imaging in humans. *Laryngoscope Investigative Otolaryngology*, *6*(1), 137–144. https://doi.org/10.1002/lio2.510

Zhang, F., Cheong, D., Khan, A. F., Chen, Y., Ding, L., & Yuan, H. (2021). Correcting physiological noise in whole-head functional near-infrared spectroscopy. *Journal of Neuroscience Methods*, *360*, 109262. https://doi.org/10.1016/j.jneumeth.2021.109262

Zhang, J., Zhang, J., Ren, H., Liu, Q., Du, Z., Wu, L., Sai, L., Yuan, Z., Mo, S., & Lin, X. (2021). A look into the power of fnirs signals by using the welch power spectral estimate for deception detection. *Frontiers in Human Neuroscience*, *14*, 578. https://doi.org/10.3389/fnhum.2020.606238

Zhang, M., Jia, H., & Wang, G. (2021). Interbrain synchrony of team collaborative decision-making: An fNIRS hyperscanning study. *Frontiers in Human Neuroscience*, *15*, 393. https://doi.org/10.3389/fnhum.2021.702959

Zhang, N., Yuan, X., Li, Q., Wang, Z., Gu, X., Zang, J., Ge, R., Liu, H., Fan, Z., & Bu, L. (2021). The effects of age on brain cortical activation and functional connectivity during video game-based finger-to-thumb opposition movement: A functional near-infrared spectroscopy study. *Neuroscience Letters*, *746*, 135668. https://doi.org/10.1016/j.neulet.2021.135668

Zhang, R., Zhou, X., Feng, D., Yuan, D., Li, S., Lu, C., & Li, X. (2021). Effects of acute psychosocial stress on interpersonal cooperation and competition in young women. *Brain and Cognition*, *151*, 105738. https://doi.org/10.1016/j.bandc.2021.105738

Zhang, T., Zhang, J., Huang, J., Zheng, Z., & Wang, P. (2021). Neural activation via acupuncture in patients with major depressive disorder: A functional near-infrared spectroscopy study. *Frontiers in Psychiatry*, *12*, 1907. https://doi.org/10.3389/fpsyt.2021.669533

Zhang, Y., Lin, X., Bi, A., Cao, N., Zhang, T., Wang, S., Wen, Y., & Bi, H. (2022). Changes in visual cortical function in moderately myopic patients: A functional near-infrared spectroscopy study. *Ophthalmic and Physiological Optics*, *42*(1), 36–47. https://doi.org/10.1111/opo.12921

Zhang, Y., Shi, W., Wang, H., Liu, M., & Tang, D. (2021). The impact of acute exercise on implicit cognitive reappraisal in association with left dorsolateral prefronta activation: A fNIRS study. *Behavioural Brain Research*, *406*, 113233. https://doi.org/10.1016/j.bbr.2021.113233

Zhao, C., Schiessl, I., Wan, M. W., Chronaki, G., & Abel, K. M. (2021). Development of the neural processing of vocal emotion during the first year of life. *Child Neuropsychology*, *27*(3), 333–350. https://doi.org/10.1080/09297049.2020.1853090

Zhao, H., Chen, J., & Lin, Y. (2021). Intelligent recognition of hospital image based on deep learning: The relationship between adaptive behavior and family function in children with ADHD. *Journal of Healthcare Engineering*, 1–11. https://doi.org/10.1155/2021/4874545

Zhao, H., Cheng, T., Zhai, Y., Long, Y., Wang, Z., & Lu, C. (2021). How mother–child interactions are associated with a child's compliance. *Cerebral Cortex*, *31*(9), 4398–4410. https://doi.org/10.1093/cercor/bhab094

Zhao, H., Li, Y., Wang, Y., Wang, X., Kan, Y., Yang, T., Hu, W., & Duan, H. (2021). Acute stress makes women's group decisions more rational: A functional near-infrared spectroscopy (fNIRS)–based hyperscanning study. *Journal of Neuroscience, Psychology, and Economics*, *14*(1), 20–35. https://doi.org/10.1037/npe0000138

Zhao, L., Kojima, H., Yasunaga, D., & Irie, K. (2021). Syntactic and semantic processing in japanese sentence reading: A research using functional near-infrared spectroscopy (fNIRS). *Journal of Psycholinguistic Research*, 1–17. https://doi.org/10.1007/s10936-021-09818-8

Zhao, L., Yasunaga, D., & Kojima, H. (2021). Similarities and differences between native and non-native speakers' processing of formulaic sequences: A functional near-infrared spectroscopy (fNIRS) study. *Journal of Psycholinguistic Research, 50*(2), 397–416. https://doi.org/10.1007/s10936-019-09655-w

Zhao, N., Zhu, Y., & Hu, Y. (2021). Inter-brain synchrony in open-ended collaborative learning: An fNIRS-hyperscanning study. *Journal of Visualized Experiments*, *173*. https://doi.org/10.3791/62777

Zhao, T., Hu, A., Su, R., Lyu, C., Wang, L., & Yan, N. (2022). Phonetic versus spatial processes during motor-oriented imitations of visuo-labial and visuo-lingual speech: A functional near-infrared spectroscopy study. *European Journal of Neuroscience*, *55*(1), 154–174. https://doi.org/10.1111/ejn.15550

Zhao, W., Hui, M., Zhang, X., & Li, L. (2021). The relationship between motor coordination and imitation: An fNIRS study. *Brain Sciences*, *11*(8), 1052. https://doi.org/10.3390/brainsci11081052

Zhao, Y., Xiao, X., Jiang, Y., Sun, P., Zhang, Z., Gong, Y., Li, Z., & Zhu, C. (2021). Transcranial brain atlas-based optimization for functional near-infrared spectroscopy optode arrangement: Theory, algorithm, and application. *Human Brain Mapping*, *42*(6), 1657–1669. https://doi.org/10.1002/hbm.25318

Zheng, J., Shi, P., Fan, M., Liang, S., Li, S., & Yu, H. (2021). Effects of passive and active training modes of upper-limb rehabilitation robot on cortical activation: A functional near-infrared spectroscopy study. *NeuroReport*, *32*(6), 479–488. https://doi.org/10.1097/WNR.0000000000001615

Zheng, Y., Tian, B., Zhang, Y., & Wang, D. (2021). Effect of force accuracy on hemodynamic response: An fNIRS study using fine visuomotor task. *Journal of Neural Engineering*, *18*(5), 056020. https://doi.org/10.1088/1741-2552/abf399

Zhou, X., Planalp, E. M., Heinrich, L., Pletcher, C., DiPiero, M., Alexander, A. L., Litovsky, R. Y., & Dean, D. C. (2022). Inhibitory control in children 4–10 years of age: Evidence from functional near-infrared spectroscopy task-based observations. *Frontiers in Human Neuroscience*, *15*. https://doi.org/10.3389/fnhum.2021.798358

Zhu, Y., Sun, F., Chiu, M. M., & Siu, A. Y.-S. (2021). Effects of high-intensity interval exercise and moderate-intensity continuous exercise on executive function of healthy young males. *Physiology & Behavior*, *239*, 113505. https://doi.org/10.1016/j.physbeh.2021.113505

Zhu, Y., Weston, E. B., Mehta, R. K., & Marras, W. S. (2021). Neural and biomechanical tradeoffs associated with human-exoskeleton interactions. *Applied Ergonomics*, *96*, 103494. https://doi.org/10.1016/j.apergo.2021.103494

Zohdi, H., Scholkmann, F., & Wolf, U. (2021). Long-Term blue light exposure changes frontal and occipital cerebral hemodynamics: Not all subjects react the same. In *Oxygen Transport to Tissue XLII* (pp. 217–222). Springer International Publishing. https://doi.org/10.1007/978-3-030-48238-1_34

Zuniga, J. M., Pierce, J. E., Copeland, C., Cortes-Reyes, C., Salazar, D., Wang, Y., Arun, K. M., & Huppert, T. (2021). Brain lateralization in children with upper-limb reduction deficiency. *Journal of NeuroEngineering and Rehabilitation*, *18*(1), 1–14. https://doi.org/10.1186/s12984-020-00803-1
